# On Aromaticity of the Aromatic α-Amino Acids and Tuning of the NICS Indices to Find the Aromaticity Order

**DOI:** 10.1021/acs.jpca.2c00346

**Published:** 2022-05-26

**Authors:** Wojciech M. Dudek, Sławomir Ostrowski, Jan Cz. Dobrowolski

**Affiliations:** Institute of Nuclear Chemistry and Technology, 16 Dorodna Street, 03-195 Warsaw, Poland

## Abstract

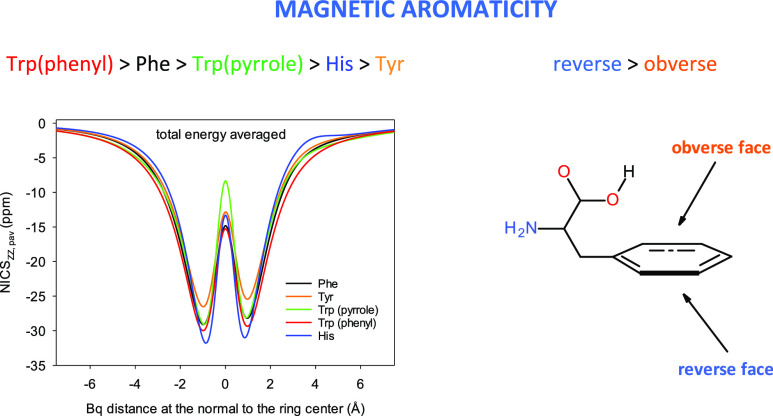

The NICS aromaticity indices of the rings in flexible phenylalanine (Phe), tryptophan (Trp), tyrosine (Tyr), and histidine (His) chiral molecules were analyzed. These molecules have several dozens of conformers, and their rings are slightly non-planar. Therefore, the population-averaged NICS_pav_ index was defined, and the NICS scans had to be performed with respect to planes found by the least-squares routine. A rule differentiating an obverse and a reverse ring face in aromatic amino acids was formulated. The NICS scan minima corresponding to the obverse and reverse face were unequal, which prompted us to use the term ring face aromaticity/ring face tropicity. It appeared that for Phe, Trp, Tyr, and His, the reverse face has always had higher ring face aromaticity/ring face tropicity than the obverse one. Despite the NICS modifications, uncertainty about the amino acid aromaticity order remained. This motivated us to use the integral INICS index newly proposed by Stanger as well. Then, the following sequence was obtained: Trp(phenyl) > Phe > Trp(pyrrole) > His > Tyr. The juxtaposition of the INICS indices of amino acids with that of some model rings revealed a fair transferability of the values. Finally, analysis of the substituent effect on INICS demonstrated that the aromaticity of Tyr is the lowest due to the strength of the OH group π-electron-donating effect able to perturb enough the ring charge distribution and its magnetic aromaticity. The NICS calculations were executed using the ARONICS program written within the project.

## 1 Introduction

The chapter on arenes by Roberts and Caserio begins as follows: “The so-called aromatic hydrocarbons, or arenes, are cyclic unsaturated compounds that have such strikingly different chemical properties from conjugated alkenes (polyenes) that it is convenient to consider them as a separate class of hydrocarbon.”^1^ It is thus clear that even if aromaticity is argued to be suspicious^2^ or ill-defined,^3^ this concept is in the center of organic chemistry^4^ and allows rationalization of the behavior of “cyclic unsaturated compounds.” Aromaticity requires an enumerative^4,5^ rather than a strict and simple definition which addresses the (1) energetic,^6^ (2) geometric,^7^ (3) magnetic,^8^ (4) electronic (electron density),^9^ (5) chemical reactivity,^6^ (6) graph theory,^10,11^ and other^4^ criteria. Each of the aromaticity conditions has been used to formulate the aromaticity index, allowing for a quantitative evaluation of this very aromaticity aspect.^4^ Different indices do not always strongly correlate with one another.

Regardless of the aromaticity aspect, the majority of studies have been focused on planar ring systems uninvolved in conformational equilibria.^4−11^ However, recently, problems with the evaluation of aromaticity in non-planar and/or non-rigid molecules have been raised.^12−14^ It seems that the more novel and the more useful a molecule is, the less planar and the more flexible the rings need to be analyzed for their aromaticity.^15−20^ Interestingly, one of the issues which, although signalized,^21^ has not been systematically studied is the variation of the aromaticity indices with the substituent conformation. This is because even for relatively simple molecules like biogenic amino acids, several hundreds of conformers have to be taken into account.^22^ Conformational complexity of non-rigid molecules has been an obstacle in the estimation of all aromaticity indices, except for conformationally invariant topological ones. Yet, when planning this study, we suspected that the NICS magnetic aromaticity index (nucleus-independent chemical shift)^23^ and the NICS scan, that is, variation of the NICS index probed at the normal to the ring at its center with the probe point distance from the center, might be especially sensitive to conformational changes.

The NICS index was originally established as NICS(0), that is, the opposite value of the absolute magnetic shielding calculated for the probe point at the ring center.^23^ The lower the NICS(0), the more magnetically aromatic the ring is. The NICS(0) values around −10.0, 0.0, and 10.0 ppm denote that the ring is, respectively, aromatic, non-aromatic, and antiaromatic.^23^ Soon, NICS(1), providing the analogous value at 1 Å over the ring center, was shown to yield more robust results.^24^ Then, the NICS(π),^25^ NICS_ZZ_,^26^ and NICSπ_ZZ_^27^ versions of the index were proven to filter the undesirable σ-orbital contribution better than NICS(1). Now, the NICS scan is preferred because it provides a new indication of diamagnetic and paramagnetic ring currents.^28^ However, recently, it has been suggested that the integrated value of NICSπ_ZZ_, ∫NICSπ_ZZ_, is also worth considering.^29^ Still, for the non-planar structures with magnetically inequivalent ring sides, the NICS(1) index is split into two NICS(1) and NICS(−1) indices,^13^ and the NICS scans are asymmetric.^30^ Therefore, now, it appears that a series of NICS values at the normal to the ring at both sides seems to be indispensable to properly evaluate the magnetic aromaticity of non-planar or asymmetrically surrounded rings.

Biogenic aromatic α-amino acids, for example,^31−34^ phenylalanine (Phe), tryptophan (Trp), tyrosine (Tyr), and histidine (His), are called so because they contain aromatic systems determining protein residue interactions with the environment. However, imidazole in the His residue chelates metal ions in metalloproteins, which is so important that His aromaticity is often omitted. From the other point of view, the rings in the aromatic α-amino acids have the “side-chains” with a remarkable conformational space. To our knowledge, the influence of the aromatic α-amino acid conformation on their aromaticity has not been studied yet.

For the studied amino acids, all neutral conformers stable in a vacuum were found at the adopted DFT level. The NICS_ZZ_ scans along the normal to the ring at its center, including NICS_ZZ_(1), NICS_ZZ_(−1), and other selected points corresponding to each of the ring sides, as well as INICS_ZZ_, that is, (an approximation of) the integral of the NICS_ZZ_ scan, ∫NICS_ZZ_, were calculated and analyzed owing to their variability, asymmetry against the ring plane, and contribution to the corresponding population-averaged NICS indices. Based on the above NICS indices, magnetic aromaticity of the biogenic aromatic α-amino acids and histidine in a vacuum was discussed.

## 2 Calculations

All DFT calculations were performed using the aug-cc-pVTZ^35,36^ basis set and the B3LYP^37−39^ functional with the inclusion of the D3 Grimme correction for dispersion forces for structure optimization.^40,41^ The large correlation consistent aug-cc-pVTZ basis set and Grimme’s correction for dispersion forces were used to reproduce the intramolecular interactions generated by the change of conformations as good as possible and thus to minimize the possible errors coming from an inadequate reproduction of these interactions. On the other hand, the most widely used B3LYP functional is sparingly parameterized with only three parameters, and, when supplemented by a dispersion correction for the equilibrium bond lengths and the equilibrium energies, it performs similarly to the modern Grimme’s ωB97XD one.^42^ The amino acid conformer generation and pre-optimization were performed at the semi-empirical level by using the Spartan’14 program^43^ and then the re-optimization of all structures was performed at the DFT level by using the Gaussian 09 suite of programs.^44^ All conformers were true minima, showing no imaginary harmonic frequencies. For an *n* single-bond structure, 3^*n*^ conformers were initially calculated with molecular mechanics. However, the subsequent DFT optimization yielded a much lower number of stable conformers, which were finally checked for structure repetition. The XYZ coordinates of all optimized structures are collected in the Supporting Information. The NICS values were calculated using the GIAO approach at the adopted level of theory.^45,46^

The conformer energetics and population according to the total and Gibbs free energies are listed in Tables S1–S7 of Supporting Information. Hereafter, we use populations estimated for 298.15 K calculated based on total energy differences between conformers and the Maxwell Boltzmann distribution equation. The NICS values averaged according to the Gibbs free energies differ only slightly from those obtained according to their total energies. The harmonic frequencies, necessary to obtain Gibbs free energies, require much longer computation times and larger computer memories and cannot be done in every laboratory for large systems. They are not always performed in the NICS-oriented aromaticity calculations. Therefore, to facilitate further comparison, in the main text, we show only values averaged according to the total energies, while those averaged according to the Gibbs free energies are given in the Supporting Information.

The *in-house ARONICS* computer program was written for the NICS index calculations using Gaussian. For the optimized molecule, ARONICS generates a Gaussian input file containing dummy atoms for calculations of the NMR shielding constants and for plotting the NICS scan. Positions of the probe points are calculated in the following way: first, the program automatically finds rings in the input structure. Then, for each ring found, it determines a plane by the least-squares fitting applied to the coordinates of the ring heavy atoms and the vector normal to this plane at the ring center. The probe points are placed along the normal straight line, and for different intervals, the step size can be varied. Here, the step was 0.1 Å for ⟨−5; 5⟩ and 0.3 Å for ⟨−9.8, −5.0⟩∪⟨5.0, 9.8⟩, where the interval limits denote distances from the ring center. Subsequently, NMR calculations for each ring can be run, after which ARONICS reads the output files and returns the NICS values at all probe points, the NICS scan plot, as well as the integral of the NICS scan. The program is available upon request.

Despite reservations about the credibility of the NICS_ZZ_ index, we use this very descriptor throughout the study. There are two reasons for that. First, the more non-planar the ring is, the less the σ–π separability is justified.^47^ This is because the π-molecular orbitals have the nodal plane within the ring plane. As the plane becomes more and more distorted, the π-orbitals become increasingly mixed with the σ-orbitals located in that distorted plane, and the σ–π separation is less and less feasible. We herein studied distorted rings in chiral systems; therefore, we decided to use a well-defined NICS_ZZ_ index instead of struggling with a σ–π separation problem that is hard to solve in non-planar aromatics. Second, we believed that for a non-observable, the simpler the molecular descriptor, the more useful it is. Therefore, here, we ground our reasoning only on the ZZ components of the magnetic shielding tensors, where *Z* is the direction of the normal to the ring, and we skipped additional analysis requiring extra assumptions.

## 3 Results and Discussion

### 3.1 NICS(1) Index vs the NICS(ext) Index Taken in an Extremum

The widely used NICS_ZZ_(1) aromaticity criterion is arbitrary^24^ and likely to be not the optimal one as the extrema of the NICS_ZZ_ scan often correspond to distances different from 1 Å.^28^ Although using the NICS(1) indices facilitates the comparison of aromaticity of different rings, the definition of the index based on the local extremum refers to a much more robust mathematical property of the NICS_ZZ_ function than the value determined somewhere at the function shoulder. Still, it should be cleared that the NICS_ZZ_ scan minimum in diatropic systems is a result of mutual compensation of the negative NICS_ZZ_ values and the positive contribution from the σ electrons.^48^ As the distance increases, the former slowly asymptotically approaches zero from the negative side, while the latter decays fast. Thus, principally, using NICS_ZZ_(min) as a quantitative measure of aromaticity is first of all justified for systems with constant contributions of the σ electrons. Although this can be accepted for a series of conformers of the same aromatic amino acid, for different rings in different amino acids, this is only an approximation justified by the fact that the different minima are at distances within a slim interval of ±0.1 Å. Also, the drawback of the descriptor defined in the extremum is a necessity to know the NICS function around the extrema. Moreover, the X-coordinates of the extrema vary with the computational level and the probing method.^49,50^ The NICS_ZZ_(ext(*i*)) index in the *i*-th extremum, ext(*i*), is defined as follows
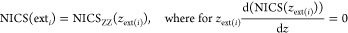
1

Notice that NICS_ZZ_(0) usually satisfies eq 1, and it often corresponds to the local maximum in aromatic compounds. For most of the planar and sparse non-planar rings, there are two identical NICS_ZZ_(min) minima corresponding to the two ring faces. On the contrary, for most non-planar rings, NICS_ZZ_(min_1_) differs from NICS_ZZ_(min_2_) to some extent.^13^ More than one minimum for a given ring face may also exist in complex structures like, for example, atom–ring complexes.

### 3.2 NICS Index of the Ring in a Flexible Molecule

Estimation of the NICS indices of rings in flexible chiral molecules raises some technical problems. Such molecules have conformers, and if we are looking for an overall NICS characteristic rather than the NICS value of an individual conformer, the conformer population should be taken into account.

**Scheme 1:**
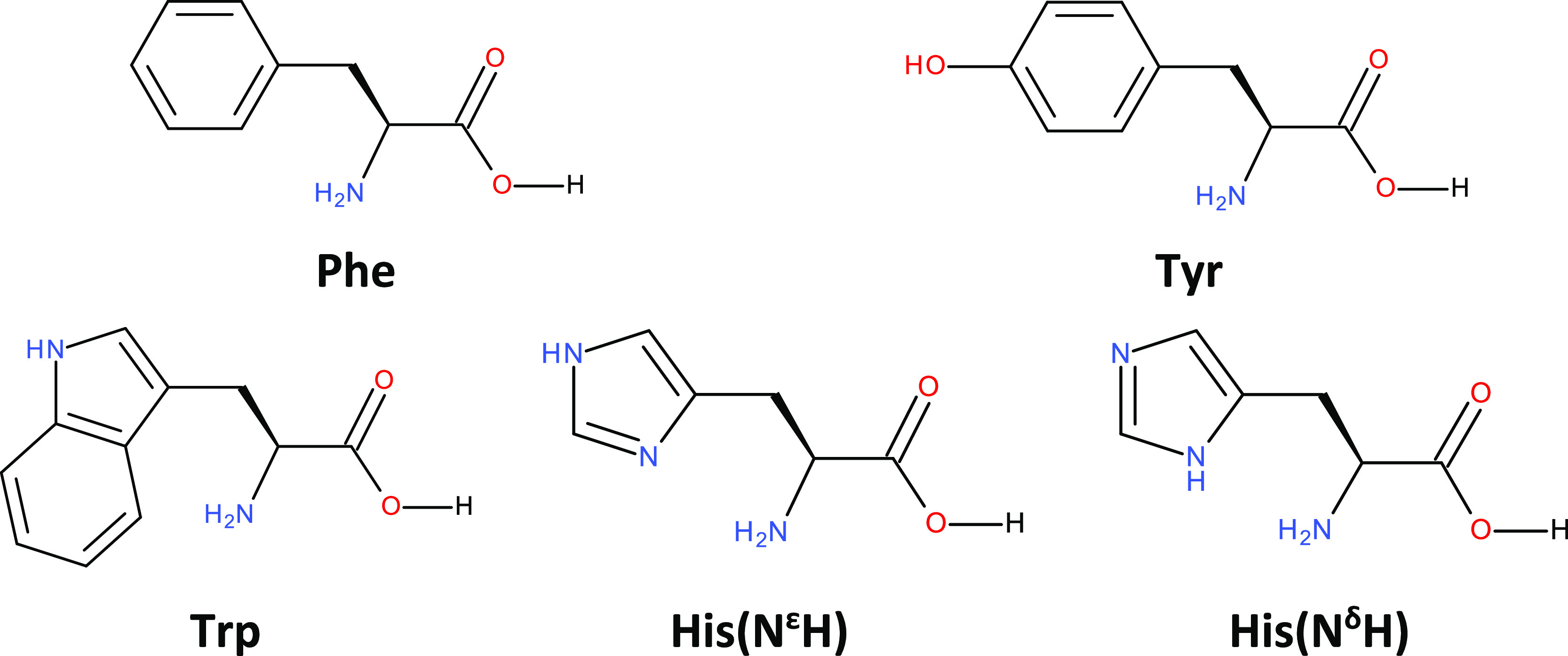
Chemical Structures of the Studied Amino Acids For His, two tautomeric forms are shown.

Here, the amino acids are considered in a vacuum, which although is not a physiological environment still not a very exotic one.^51−61^ In the diluted gas phase and low-temperature inert gas matrices, the amino acids remain in their native neutral form, and the presence of zwitterions or dimers can be neglected.^51,52,62−64^ The Phe, Trp, Tyr, and His amino acids studied here (Scheme 1) contain phenyl, indole (cumulated phenyl and pyrrole), *p*-hydroxyphenyl, and imidazole rings, respectively.

The number of conformers found for Phe, Trp, Tyr, and His at the B3LYP/D3/aug-cc-pVTZ level is 21, 37, 38, and 67, respectively (Tables S1–S7). In a vacuum, the number of conformers is determined by the possibility of both (i) rotations around single bonds and (ii) intramolecular attractive and repulsive interactions. This is why, for apparently similar molecules, different numbers of conformers can be found. However, for His, the number is larger because of the co-existence of the N^ε^H or N^δ^H tautomers.^61^ The molecules in a vacuum with moderate conformational freedom, like α-amino acids, have several dozens of conformers.^22^ Therefore, to evaluate their aromaticity, there is a need to introduce the population-averaged NICS indices, whose formula is straightforward and proper for any type of index describing a set of conformers
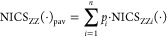
2where the pav subscript denotes the population-averaged value, *n* is the number of conformers, and *p*_*i*_ and NICS_ZZ*i*_(·) denote the population weight and the NICS_ZZ_ value of the *i*-th conformer, respectively. The NICS_ZZ_(·) index represents any of the NICS indices used for characterizing the rings in a series of conformers. For reasons listed in the Calculation Section, hereafter, only populations based on total energies and *T* = 298.15 K are considered. In a vacuum, the three most energetically feasible conformers account for over 70% of a given system (Tables S2–S6).

### 3.3 NICS Indices of Non-Planar Rings

The rings in chiral aromatics are often non-planar with two ring faces having different NICS indices,^13^ which indicates that one face is more aromatic than the other. The equivalence can occur if the symmetry of a chiral molecule is higher than *C*_1_, for example, the *C*_2_ symmetry of 1,4,5,8-tetrabromonaphthalene. Phe, Tyr, Trp, and His molecules have no symmetry elements other than identity; hence, their rings are slightly non-planar, and two faces of these rings are magnetically inequivalent. In ref (13), we proposed a convention in which −1 and 1 in NICS(−1) and NICS(1) indices denoted the points at the concave and the convex side of the molecule, respectively. For example, for corannulene, −1 corresponded to the inside and 1 to the outside of the bowl. If the convex and concave sides could not be defined, by −1 we denoted the point corresponding to lower NICS: NICS(−1) < NICS(1). Here, we used NICS(min_1_) and NICS(min_2_) as defined in eq 1.^13^ However, it is not always clear a priori how to differentiate the faces and whether in a series of conformers the same kind of face is always the more aromatic one.

**Scheme 2:**
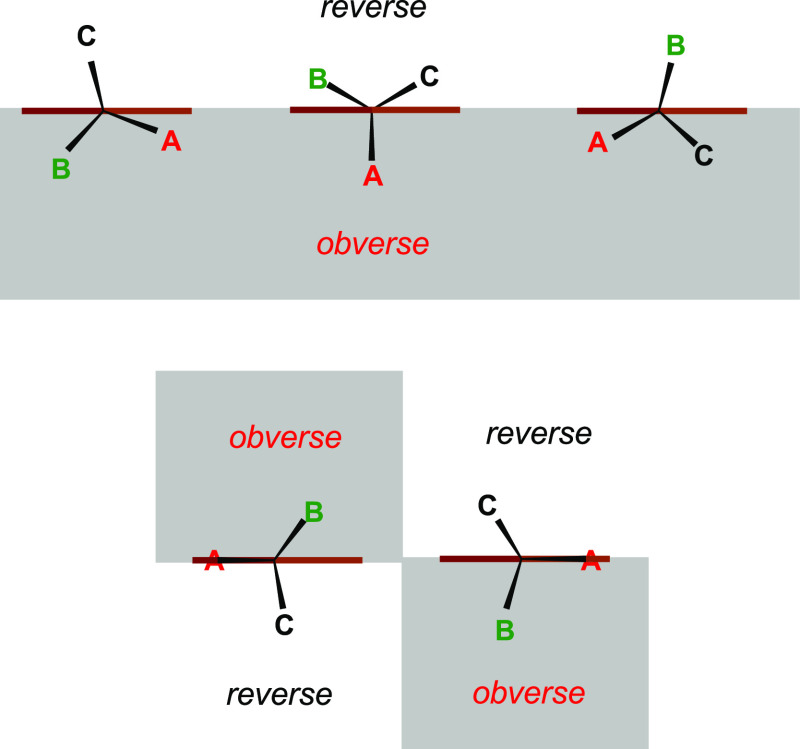
Convention for the Unequivocal Assignment of the Ring Face in the Aromatic Amino Acids Studied (and Monosubstituted Aromatic Rings, Where the Directly Attached C-Atom is Asymmetric) The brown segment represents the ring system in a plane perpendicular to the paper sheet. The C**ABC** asymmetric carbon atom substituents are directed to the viewer. **A**, **B**, and **C** denote groups listed according to the decreasing substituent CIP priority. Terms “obverse” and “reverse” are the proposed names of the ring sides. The rule for the **CA**_2_**B** and **CAB**_2_ substituents is clarified in the text.

We found that there is a simple ring face differentiation if a single asymmetric carbon atom is attached directly to the ring. Consider such a ring placed in the plane perpendicular to the sheet of paper so that its perpendicular projection to this sheet is close to the horizontal segment representing the ring’s plane projection (where the word “close” is used because of the ring’s non-planarity) (Scheme 2). Let the asymmetric carbon atom be directed to the viewer and A, B, and C denote the asymmetric atom substituents listed according to the decreasing Cahn–Ingold–Prelog (CIP) priority.^65^

Then, the highest priority substituent A can be over or below the plane (brown segment, Scheme 2). For the non-planar rings, we consider the plane for which the square root of the sum of squared distances of the ring heavy atoms is minimal. The ring face concordant with the position of the highest priority substituent will be called the obverse (obv) face, while the opposite one will be called the reverse (rev) one. If the highest priority substituent is placed exactly in the ring plane, the middle priority substituent determines the obverse, while the lowest priority one indicates the reverse side. Notice that for both enantiomers *R* and *S*, the obverse sides will be placed at the highest priority substituent side. Thus, in the reflection (mirror image transformation, Ref(·), the obverse side of the ***R***_i_ ring in the *S* enantiomer, obv_*S*_(***R***_i_), is transformed onto obv_*R*_(***R***_i_), that is, the obverse side of the ***R***_i_ ring in the *R* enantiomer, and rev_*S*_(***R***_i_) is transformed onto rev_*R*_(***R***_i_)
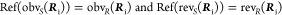
3

However, in the α-amino acids studied, the attached carbon atom is of the CA**BB** type, where **A** = C(NH_2_)COOH and **B** = H. In this case, the position of the **A** group determines the obverse face of the ring, unless it is not co-planar with the ring plane. Then, if **A** is not complex, the faces cannot be distinguished. Yet, if **A** is complex, the position and substituent priority of the groups attached to **A** have to be taken into account, and again, the location of the group with the highest priority determines the obverse face. It is necessary to add that for the C**AAB** substituents, the position of group **B** determines the reverse face unless it is not positioned in the ring plane. Again, if **B** is not complex, then the faces cannot be distinguished. The generalization of the used convention is not straightforward already for the disubstituted rings. However, the more meticulous discussion goes beyond this study.

### 3.4 NICS_ZZ_ Indices of the Rings in Phe, Tyr, Trp, and His

#### 3.4.1 NICS_ZZ_ Values at Extrema

**Figure 1:**
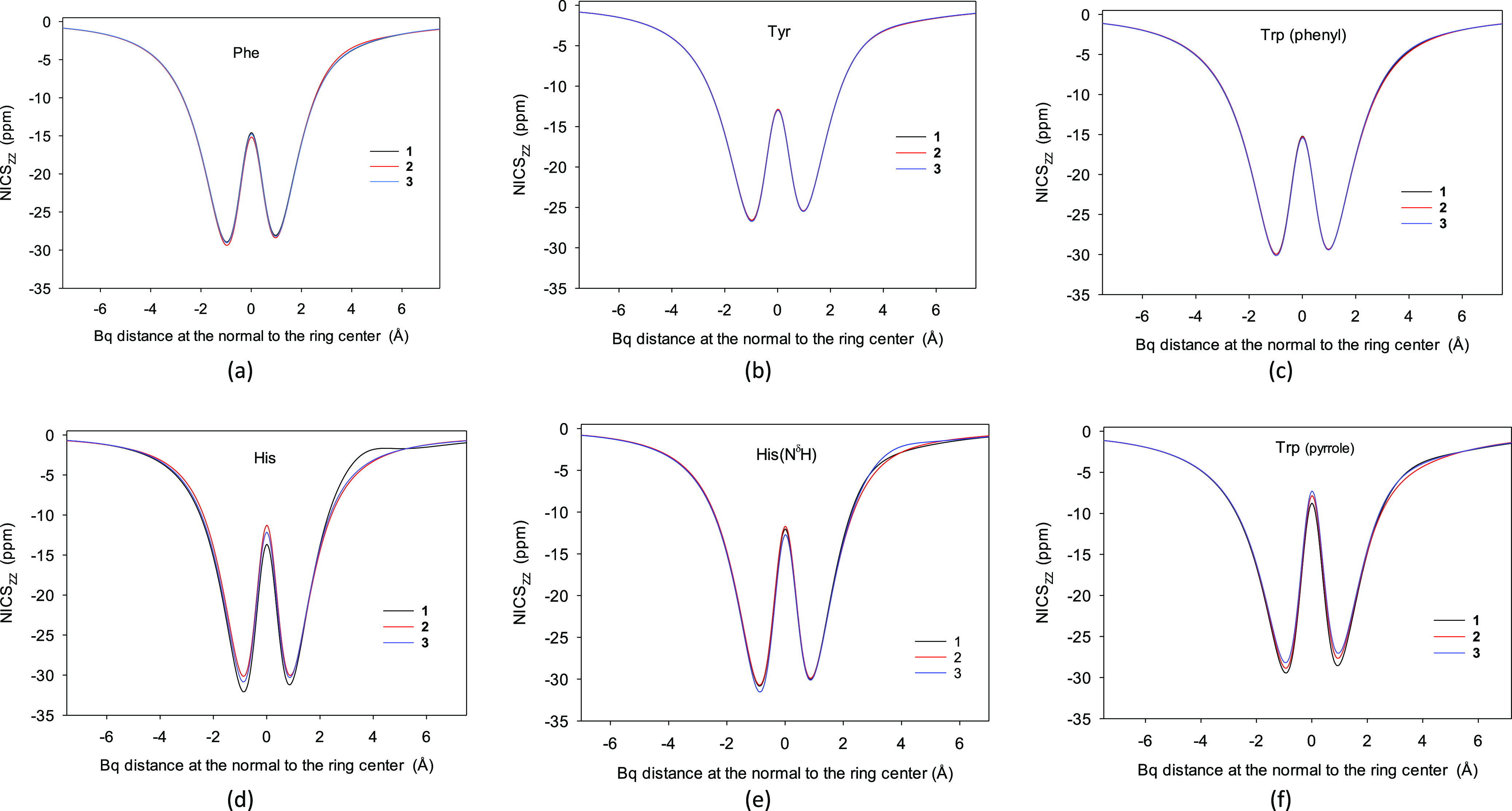
(a–f) NICS_ZZ_ scans for the aromatic rings in the three most populated Phe, Tyr, Trp, and His conformers in the gas phase calculated at the B3LYP/D3/aug-cc-pVTZ level. For all NICS_ZZ_ scans, see the Supporting Information. (e) His N^δ^H tautomer conformers are less abundant, and the scans of the most populated three N^ε^H conformers are also the most populated in the whole population of the two tautomer mixture. The reverse ring face corresponds to negative Bq distances.

For the Phe, Tyr, Trp, and His conformers, the estimated NICS_ZZ_ scans are slightly asymmetric about the 0*Y* axis, with one local minimum present at each side and the maximum at *X* = 0, while the maximal scan values are in ±∞(Figures 1, and S1–S7, Table 1). It appears that the global NICS_ZZ_ minimum is always on the reverse side of the Phe, Tyr, Trp, and His rings (the negative part of the OX axis, Figures 1 and S1–S7). Two minima were equally deep only very occasionally. Thus, for Phe, Tyr, Trp, and His
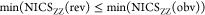
4

**Table 1:** Population-Averaged INICS_ZZ,pav_ and NICS_ZZ,pav_ Indices of Rings in the Aromatic Amino Acids and in the Selected Model Molecules^a^

	INICS_ZZ,pav_			NICS_ZZ,pav_		
molecule	(tot)	(rev)	(obv)	(minrev)	(minobv)	(0)
α-Amino Acids						
Trp(phenyl)	–155.1	–79.0	–76.2	–30.0	–29.4	–15.2
Phe	–145.0	–74.7	–70.3	–29.1	–28.2	–14.8
Trp(pyrrole)	–138.3	–71.6	–66.8	–29.1	–28.1	–8.3
His(imidazole)	–133.8	–69.0	–64.8	–31.8	–31.0	–13.3
Tyr	–128.7	–66.9	–62.4	–26.5	–25.4	–12.8
Six-Membered Rings						
benzene	–147.3	–73.6	–73.6	–29.9	–29.9	–16.1
toluene^c^	–141.9	–71.5	–70.4	–28.8	–28.4	–14.7
*p*-cresol(1)	–128.9	–64.4	–64.4	–25.8	–25.8	–12.5
*p*-cresol(2)^c^	–129.1	–64.8	–64.2	–26.0	–25.7	–12.5
3-Me-indole(1)	–157.0	–78.5	–78.5	–30.0	–30.0	–15.6
3-Me-indole(2)	–157.1	–78.6	–78.6	–30.0	–30.0	–15.6
Five-Membered Rings						
pyrrole	–142.1	–71.1	–71.1	–32.4^b^	–32.4^b^	–14.5
3-Me-indole(1)	–136.7	–68.4	–68.4	–27.8	–27.8	–7.3
3-Me-indole(2)	–136.8	–68.4	–68.4	–27.8	–27.8	–7.3
imidazole	–142.1	–71.1	–71.1	–32.4^b^	–32.4^b^	–14.5
4-Me-1H-imidazole	–136.7	–68.4	–68.4	–27.8	–27.8	–7.3
5-Me-1H-imidazole	–136.8	–68.4	–68.4	–27.8	–27.8	–7.3

a All structures are optimized at the B3LYP/D3/aug-cc-pVTZ level.

b Corresponds to ±0.9 Å.

c One CH bond of the Me group is in the plane perpendicular to the benzene plane, numbers in parentheses denote different conformers due to rotations of the Me group.

Hence, in a vacuum, the reverse ring face in the Phe, Tyr, Trp, and His molecules is always the one exhibiting higher ring face aromaticity/ring face tropicity, which may have implications when the interactions of peptides or proteins with an environment are considered. We introduce the alternative terms “ring face aromaticity” or “ring face tropicity” as a result of discussion with one of the reviewers of this paper. The controversy stems basically from adopting different positions in the philosophy of nature and, as such, is undecidable—see the Conclusions section. Tropicity (directionality) is the common term for dia- and para-tropicity.

The NICS_ZZ_ scans of the most stable three conformers (accounting for over 70% of the population) are not very different (Figure 1). The NICS_ZZ_(min_rev_) and NICS_ZZ_(min_obv_) values of the most stable three conformers in the Phe, Tyr, and Trp six-membered rings were found at ±1.0 Å, but for the His and Trp five-membered ones, they were often positioned at ±0.9 Å (Tables S1 and S8). Thus, the magnetic aromaticity of the aromatic α-amino acid molecules is relatively resistant to the conformation change.

**Figure 2:**
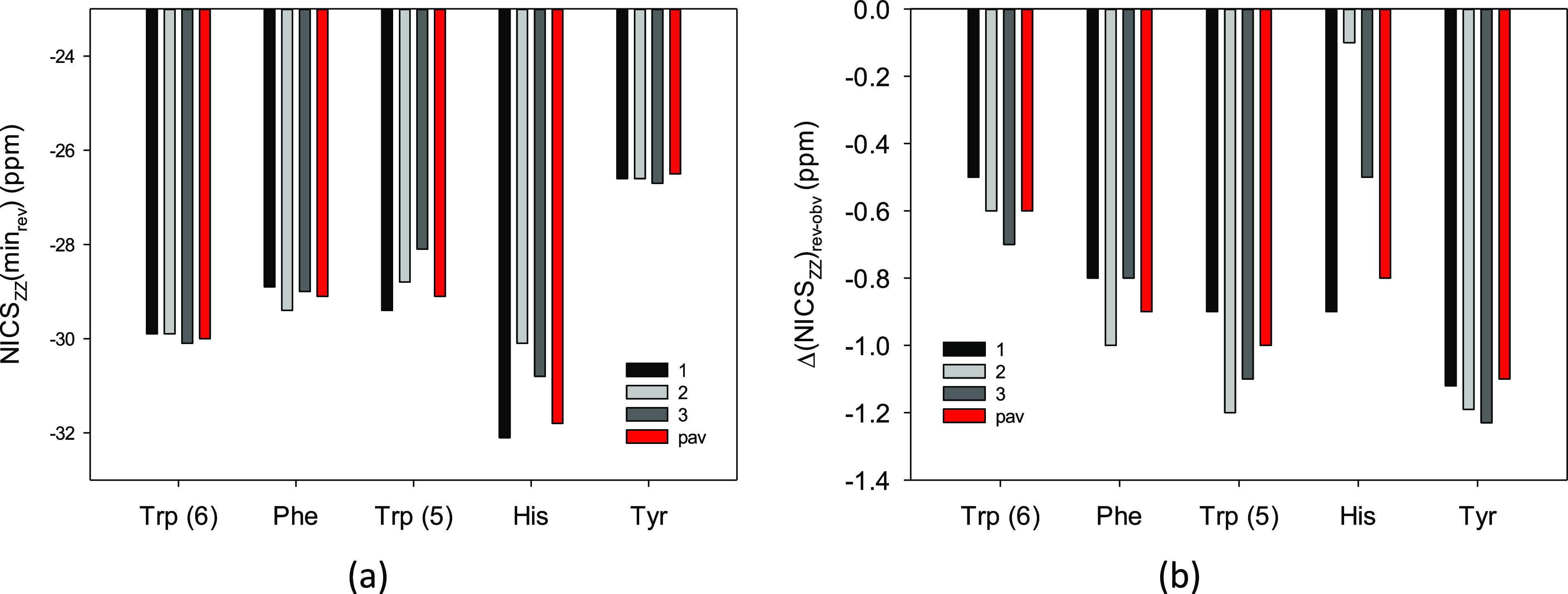
NICS_ZZ_(min_rev_) and Δ(NICS_ZZ_)_rev-obv_ aromaticity indices, (a,b), respectively, for rings in the three most populated conformers of the Phe, Tyr, Trp, and His aromatic amino acids calculated at the B3LYP/D3/aug-cc-pVTZ level, and the corresponding population-averaged NICS_ZZ_(min_rev_)_pav_ and Δ(NICS_ZZ_)_rev-obv, pav_ indices (in red).

Now, notice that NICS_ZZ_ indices of the most stable forms are not necessarily the most negative. Thus, the most stable conformer is not always the one with the highest aromaticity (Figure 2, Table S8). Indeed, for Trp(phenyl), Phe, Trp(pyrrole), His, and Tyr, the lowest NICS_ZZ_(min_rev_) was found for the third, second, first, first, and third conformers, respectively (Figure 2a). Still, the same conformers do not necessarily have the other indices as the lowest. For example, for Tyr, NICS_ZZ_(min_rev_) is the lowest for the third, while NICS_ZZ_(min_obv_) is the lowest for the first conformer (Figure 2b). This is a result of differences in the NICS_ZZ_ curve asymmetry due to the differences in the arrangements of the chiral substituents (Figures 1 and 2). As a result, it would not be proper to approximate the NICS value by, for example, one of the most stable conformers.

Second, the order of aromaticity based on NICS_ZZ_(min_rev_) for the most stable conformers is as follows: His > Trp(phenyl) > Trp(pyrrole) > Phe > Tyr. However, if we look at the second and third stable ones, positions of Phe and Trp(pyrrole) must be interchanged (Figure 2b). Nevertheless, the order given for the obverse faces by the NICS_ZZ_(min_obv_) indices is the same because NICS_ZZ_(min_rev_) – NICS_ZZ_(min_obv_) differences and Δ(NICS_ZZ_)_rev-obv_ are usually smaller than 1.5 ppm (Figure 2c, Table S8). Moreover, the sequence differences are found for the individual indices of the most stable, the second most stable, and the third most stable conformers.

#### 3.4.2 Population-Averaged NICS_ZZ_ Values

**Figure 3:**
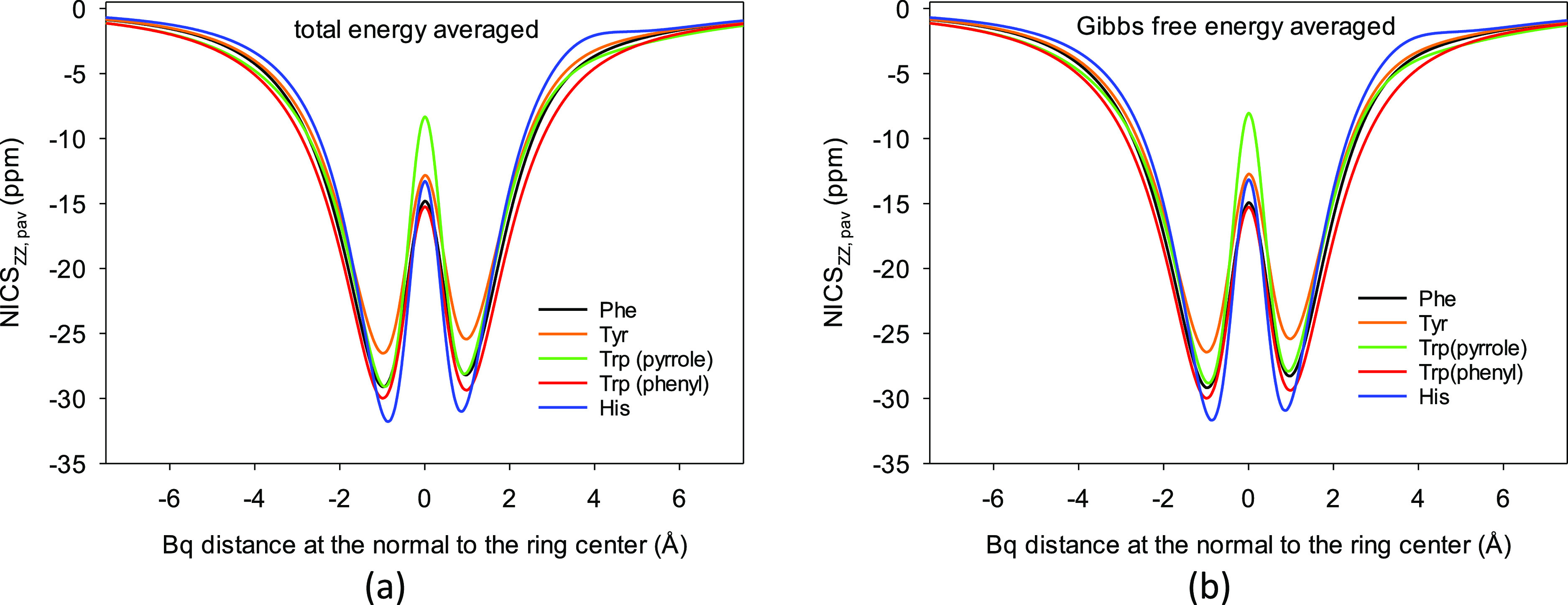
Population-averaged NICS_ZZ,pav_ scans for the Phe, Tyr, Trp, and His aromatic α-amino acids obtained using(a) total energies at 298.15 K and (b) Gibbs free energies at 298.15 K.

Let us check whether the use of population-averaged indices (Tables 1 and S8–S12), which are conformer-independent, yield a reliable order of the amino acids aromaticity. The order of aromaticity pointed out by the NICS_ZZ_(min_rev_)_pav_ index is as follows: His > Trp(phenyl) > Phe ≈ Trp(pyrrole) > His > Tyr, and it is similar as before (Figure 2a,b). Thus, the population-averaged indices yield the order of the aromatic α-amino acids similar to that provided by NICS_ZZ_ indices of the scan minima. On the other hand, the positions of Trp(5) and His in the given order are surprising: for NICS_ZZ_(min_rev_), the former is comparable to Phe, while the latter is the largest (*sic*!). Do we then obtain the proper order of the α-amino acid aromaticity based on the NICS_ZZ_ indices from the scan minima? The problem in answering this question lies in the difficulty in finding the reference points which could supply the correct order. It is worth noting though that the individual and the population-averaged scans (Figure 3) have their specific half widths, which are related to aromaticity but are not included in the single point values.

Therefore, we followed the Stanger idea of using the integral ∫NICS index,^29^ denoted here as INICS. INICS is independent of the measurement points and cumulates all information about aromaticity contained in the NICS scan along the normal to the ring center. Here, we ignore the fact that for (slightly) asymmetric rings, the maximal NICS value may be (slightly) off the ring center, and thus the global extrema can also be slightly off the normal to the ring.^29^ The integral INICS index is defined as follows
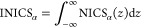
5where α describes the kind of the NICS index (zz, πzz, etc.), and *z* runs along the normal to the ring in the ring center.

Here, we approximate the INICS_α_(*z*) index by the  index which is a definite integral taken at the normal from −9.8 to 9.8 Å
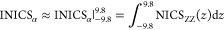
6

However, because of the scan asymmetry for the non-planar rings, we also consider integrations over the −9.8 to 0.0 Å and 0.0 to 9.8 Å intervals. For clarity, hereafter, we use notations INICS_ZZ_(tot), ININCS_ZZ_(rev), and INICS_ZZ_(obv) instead of  , , and , respectively, or the appropriate definite integral notations.

**Figure 4:**
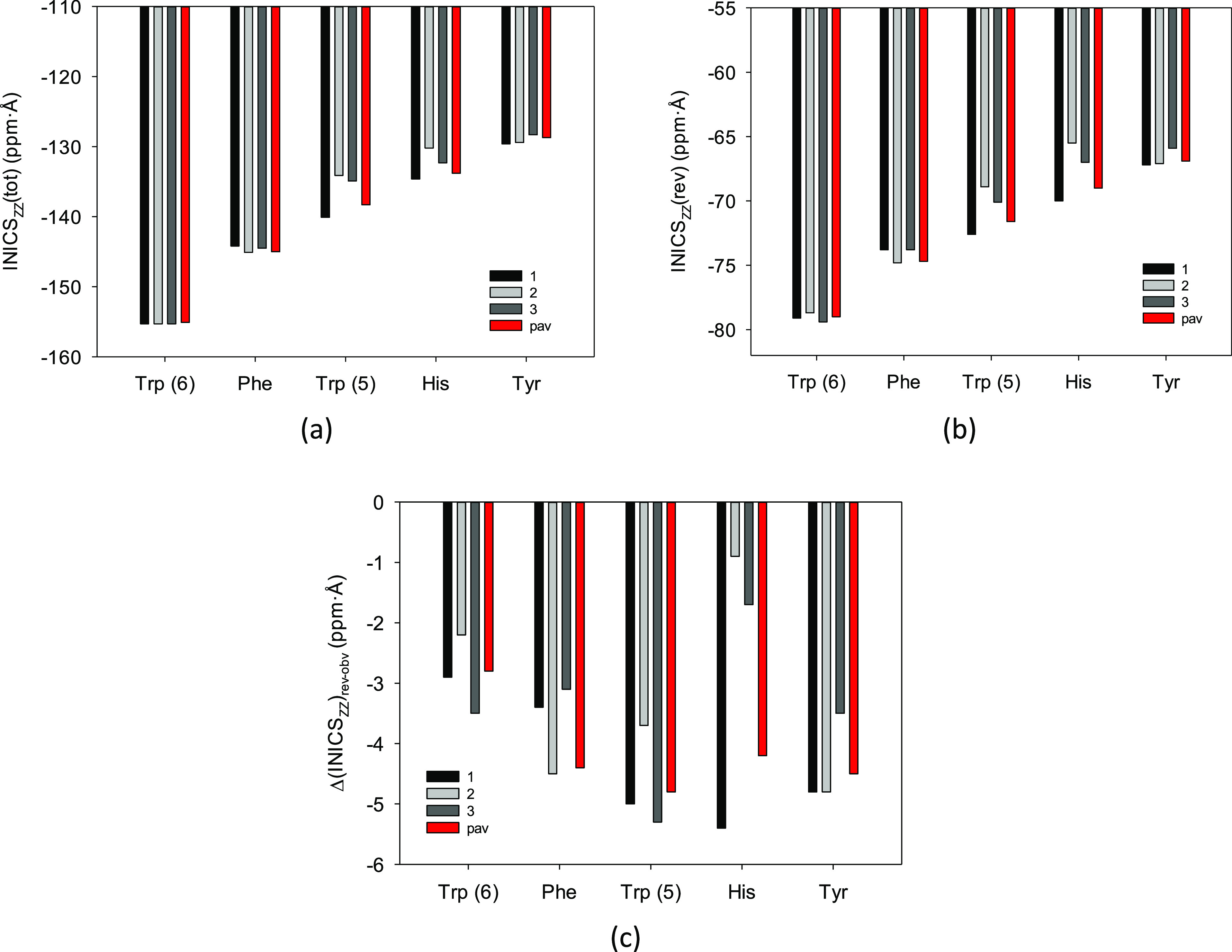
(a,c,e) INICS_ZZ_(tot), INICS_ZZ_(rev), and Δ(INICS_ZZ_)_rev-obv_ integral aromaticity indices of rings in the three most populated conformers of the Phe, Tyr, Trp, and His aromatic amino acids calculated at the B3LYP/D3/aug-cc-pVTZ level, and the corresponding population-averaged INICS_ZZ_(tot)_pav_, INICS_ZZ_(rev) _pav_, and Δ(INICS_ZZ_) _rev-obv, pav_ indices (in red).

The integral indices gathered in Table 1 (and Tables S13–S17) and illustrated in Figure 4 concordantly and unequivocally indicate that the magnetic aromaticity order of the natural α-amino acids is as follows
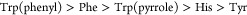
7

There are, however, few exceptions to the above rule for particular conformers. For example, the reverse face of the second His conformer is less aromatic than that of the second Tyr conformer (Figure 4). Nevertheless, the inequality (7) is satisfied by all three population-averaged integral indices. Still, are the aromaticity differences in this inequality meaningful?

**Figure 5:**
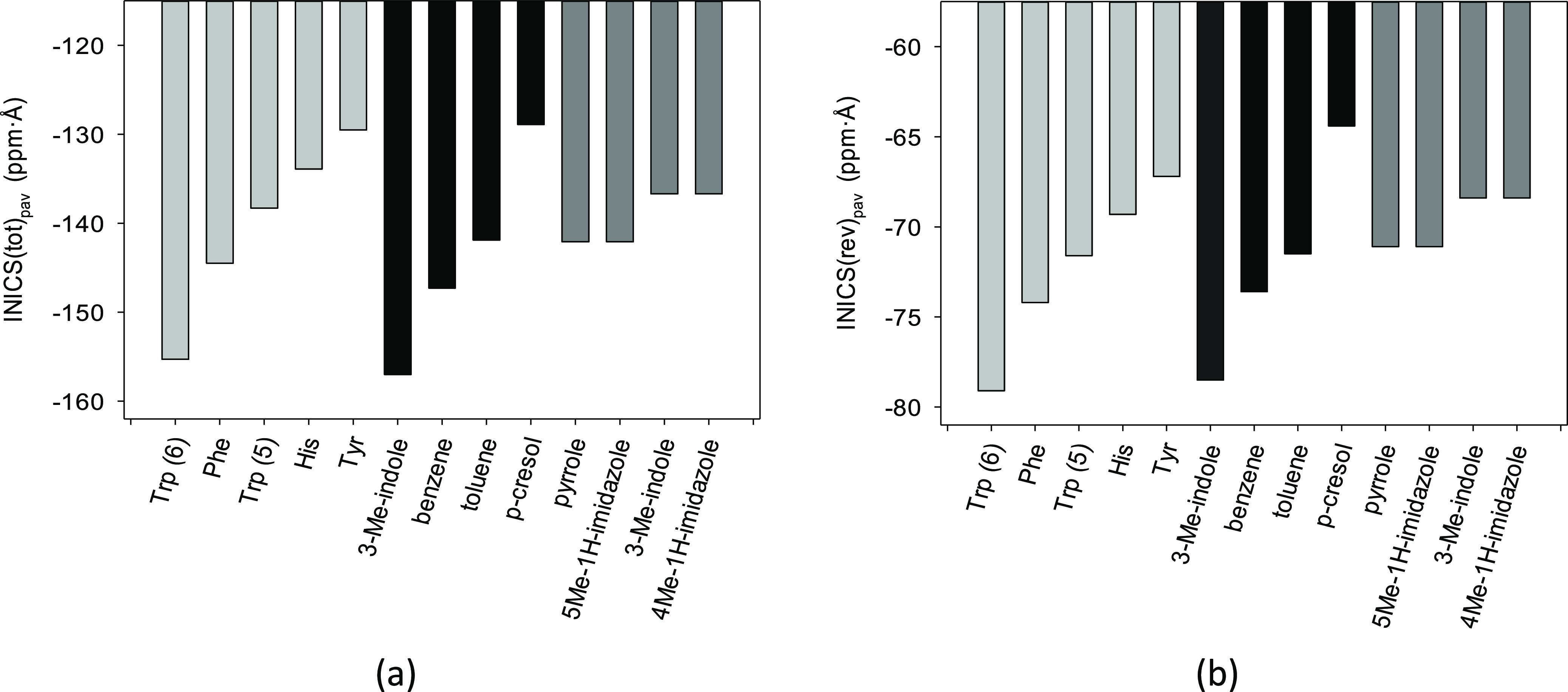
Population-averaged aromaticity indices of rings in the Phe, Tyr, Trp, and His aromatic amino acids (light gray) and selected six-membered (black) and five-membered rings (dark gray): (a) and (b) integral indices INICS_ZZ_(tot)_pav_ and INICS_ZZ_(rev)_pav_, respectively. Calculations were done at the B3LYP/D3/aug-cc-pVTZ level.

To answer this question, the population-averaged INICS_ZZ_ of α-amino acids were juxtaposed with those of model aromatic rings (Figure 5 and Table 1). Notice that the bar sizes of the model rings are fairly similar to the corresponding amino acids: the black bars to the six-membered rings in Trp(6), Phe, and Tyr, and the dark gray bars to the five-membered rings in Trp(5) and His (Figure 5). Observe also that the order of the bar sizes is preserved: the integral NICS_ZZ_ aromaticity of Trp(6) and methyl indole six-membered ring is the largest; aromaticity of Phe is a bit smaller than that of benzene but similar to that of toluene, and aromaticity of Tyr is as small as that of *p*-cresol. Same can be said about the five-membered rings: aromaticities of Trp(5) and the five-membered ring of methyl indole are similar and smaller than those of unsubstituted pyrrole. Also, the aromaticity of His is closer to that of methyl 1*H*-imidazole than that of unsubstituted imidazole. Analogous relations between the aromaticity of amino acids and model molecules are not observed for the NICS_ZZ_(max_rev_) index (Figure S8). Thus, the integral INICS index seems to be robust and indicative, and it provides meaningful aromatic inequality. The conclusion is that the NICS aromaticity of the amino acids can fairly be estimated from the corresponding aromatic cycles. However, in our opinion, this finding could not be formulated without an extensive study that considers all the amino acid conformations.

**Figure 6:**
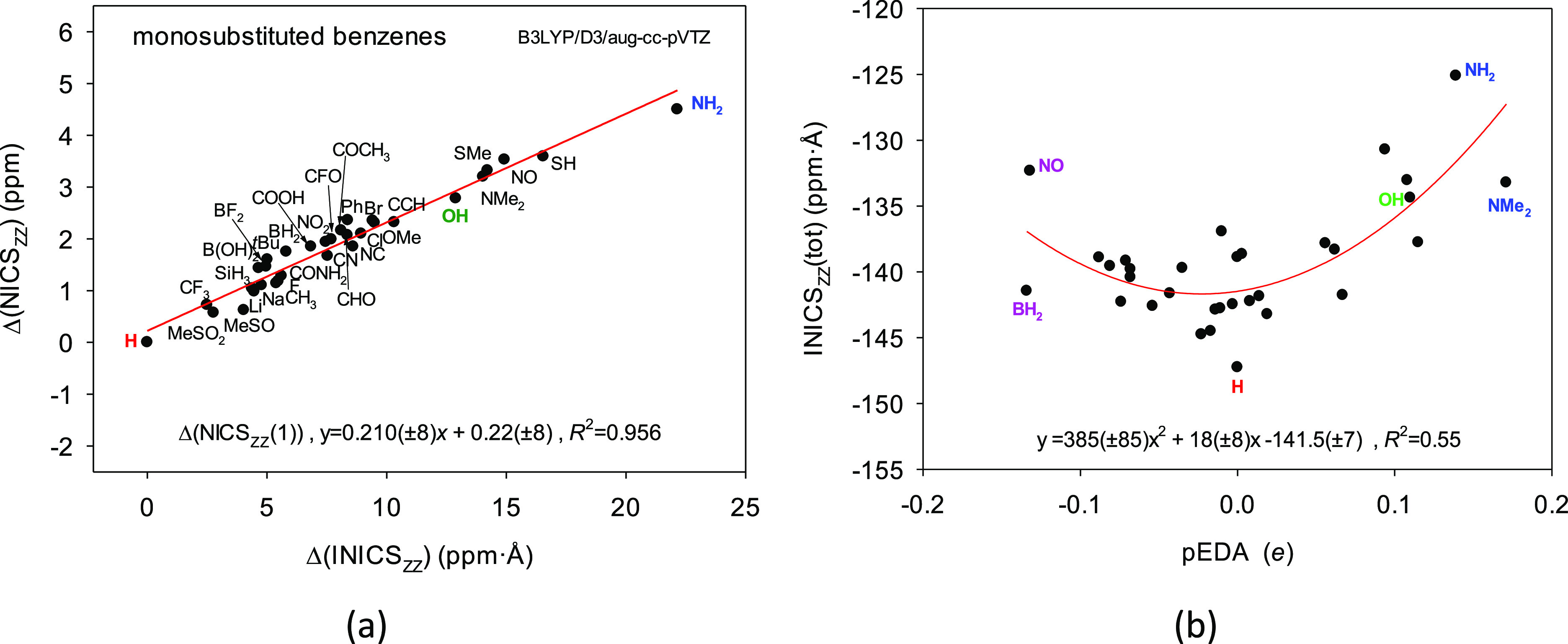
(a) Linear correlation between the differences in the integral aromaticity indices of aromatic rings in a series of benzenes monosubstituted with groups of different σ- and π-electron activities, Δ(INICS_ZZ_(tot)) = INICS_ZZ_(benzene) – INICS_ZZ_(substituted benzene) and the analogous difference for the NICS_ZZ_(1) index. (b) Weak quadratic correlation between the INICS_ZZ_(tot) index for monosubstituted benzenes and the pEDA index expressing the substituent effect on the ring’s π-electron system.^66^ Calculations were done at the B3LYP/D3/aug-cc-pVTZ level. Notice that the greater the value of the difference between indices, the smaller the NICS_ZZ_ aromaticity.

In the end, think about the reason why the aromaticity of Tyr is the smallest. To answer this question, we determined various NICS_ZZ_ indices for a series of benzenes monosubstituted with groups of different σ- and π-electron activities (Table S18, Figure S9).^66−69^ It appeared that the INICS_ZZ_ values linearly correlate with the NICS_ZZ_(1) ones. For clarity, we show the correlations between differences in the indices taken with respect to the unsubstituted benzene, which has the lowest index, that is, which is the most aromatic among monosubstituted benzenes (Figure 6a).

First, notice that the variability of the INICS_ZZ_ values covers the interval of ca. 25 ppm·Å, whereas for NICS_ZZ_(−1) and NICS_ZZ_(1) it is ca. 5 ppm (Figure 6a, Table S18). Thus, the integral index is 5 times more discriminating than the NICS_ZZ_ values in points. Second, observe that the least aromatic are those rings in monosubstituted benzenes which are substituted by the strong π-electron-donating groups like NH_2_, SH, SMe, NMe_2_, and OH (Figure 6). This indicates that supplying the free electron pair(s) to the ring increases the C(ipso)–X bond order, it acquires the double bond character and induces the bond alteration in the ring, which consequently decreases the ring aromaticity. However, it can be seen that between the substituents that significantly decrease the INICS_ZZ_ aromaticity, there are also NO, CN, CCH, and BH_2_ groups (Figure 6a). To better understand why it is so, notice the weak quadratic trend in correlation with the pEDA descriptor,^66−69^ expressing the substituent effect on the ring’s π-electron system: the negative pEDA values denote the amount of electron charge withdrawn from the ring, while the positive ones denote the amount of charge donated to the ring. The weak quadratic trend, explaining ca 55% of the INICS_ZZ_ changes (*R*^2^ = 0.55), means that both π-electron-donating and π-electron-withdrawing substituents decrease the substituted ring aromaticity. This can be interpreted as follows: strongly acting substituents strongly disturb the symmetry of the π-electron system in the ring and introduce bond alternation to the ring. Thus, they destroy the ring aromaticity. This is in line with the Stanger studies on the influence of strong π-donors and π-acceptors on the magnetic aromaticity.^48^ He found that for mono-, di (meta)-, and tri (meta)-substituted benzene with either OH (π-donor) or BH_2_ (π-acceptor) groups, the ring currents are weaker than in benzene, and the more substituted the system is, the weaker the ring current, whether it is a strong π-donor or strong π-acceptor. Finally, let us stress that OH being one of the strongest π-electron-donating substituents significantly decreases the aromaticity of the benzene ring and therefore the aromaticity of Tyr is much smaller than that of Phe or Trp(6).

## 4 Conclusions

The changeability of the NICS indices of the non-planar rings with the side-chain conformation was analyzed for the following biogenic aromatic α-amino acid molecules: phenylalanine (Phe), tryptophan (Trp), tyrosine (Tyr), and histidine (His). The B3LYP/D3/aug-cc-pVTZ calculations indicated that there are 21, 37, 38, and 67 conformers in a vacuum for Phe, Trp, Tyr, and His, respectively. To find the indices for a significantly non-rigid compound, the population-averaged index, NICS_pav_, was introduced.

The rings in the studied molecules are slightly distorted. To examine the NICS scan for a non-planar ring, it is necessary to find the ring-intersecting plane using the least-squares routine and plot the scan along the normal to such a plane at the ring center. It was also useful to introduce a rule discriminating the obverse and reverse faces of the monosubstituted rings. It came into view that for every Phe, Trp, Tyr, and His conformer, the reverse face has had higher ring face aromaticity/ring face tropicity than the obverse one. The NICS plots were asymmetric, and the minima were not always located at 1 and −1 Å above or under the ring. Therefore, the values in minima, NICS_ZZ_(min_obv_) and NICS_ZZ_(min_rev_), were used instead of the NICS(−1) and NICS(1) indices.

The NICS_ZZ_(min_obv_), NICS_ZZ_(min_rev_), and the corresponding NICS_pav_ indices exhibited only a slight variation of aromaticity with conformation, which means that the magnetic aromaticity of the aromatic α-amino acid molecules is relatively resistant to the conformation change. However, despite NICS modifications, a robust ordering of the Phe, Trp, Tyr, and His amino acids was not fully congruent.

Therefore, we introduced, after Stanger, an integral index, INICS, which appeared to be the most robust and indicative. The order pointed out by the INICS_ZZ_ and NICS_ZZ_(min_rev_)_pav_ indices was very similar: Trp(phenyl) > Phe > Trp(pyrrole) > His > Tyr. Calculation of newly defined indices was made possible, thanks to the ARONICS program written within this project.

To explain why the aromaticity of Tyr is the lowest, we determined various NICS_ZZ_ indices for a series of benzenes monosubstituted by groups with different σ- and π-electron activities. It turned out that the INICS_ZZ_ values linearly correlate with the NICS_ZZ_(1) values (in this case, the NICS minima were always at 1 Å above the ring). Interestingly, the variation of the integral index is 5 times larger than the NICS_ZZ_ values in points, thus it exhibits a much larger potential for aromaticity discrimination. Finally, values of INICS and the other indices demonstrated that the aromaticity of Tyr is the lowest due to the strength of the OH group π-electron-donating effect able to perturb enough the ring charge distribution to significantly change its magnetic aromaticity.

In the end, since it is known that the magnetic aspect of aromaticity differs from the electronic, geometric, and energetic ones,^70^ it is interesting to know the comparison between the relative aromaticity of the four aromatic amino acids based on NICS with indices of different types. For a relatively large set of conformers of the studied compounds, it is difficult to gather many possible aromaticity indices; therefore, following one of the reviewers’ suggestions, we calculated HOMA and population-averaged HOMA_pav_ indices. We found that the HOMA_pav_ indices of the aromatic amino acids were different than the INICS_pav_ ones (Tables S24 and S25). This can be interpreted in terms of the differences between the responses of geometric and magnetic aromaticity to conformational changes. However, it is very likely that a way of parametrization used to evaluate the HOMA values of heterocyclic rings additionally artificially perturbed the values and thus the comparison.

Here, let us also address the reviewer’s question about the relevance of small aromaticity variations between aromatic amino acids to biochemistry. As a result of this study, we get to know that the conformer equilibria in amino acids only weakly affect the magnetic aromaticity. Moreover, differences in the magnetic aromaticity of the aromatic amino acids are not large. However, the aromatic amino acids play a complex role in the protein structure, which inter alia includes the stacking interactions stabilizing the folded protein structures. Therefore, they are present to a large extent in the cores of globular proteins. Besides, at protein ends, they actively participate in the interprotein and protein–DNA interactions as well as in the protein–ligand interactions on the protein surface. Although the interaction of a single aromatic amino acid residue can be small in a protein consisting of several hundreds of amino acids, these small effects are (probably non-additively) magnified. This is similar to the extra stabilization of the crystal structure by many, apparently unimportant, C–H hydrogen bond-like interactions. In conclusion, even small aromaticity differences between amino acids are probably very important, but this importance is very hard to estimate, and this problem needs further studies.

Finally, let us comment on the alternative use of the “ring face aromaticity” and “ring face tropicity” terms which is a result of a controversy between the authors of this paper and one of the reviewers. The reviewer refers to “the essence of aromaticity” and argues as follows: “In the authors’ approach, it is implicated that dia-/paratropicity is equal to aromaticity/antiaromaticity. This is not the case. There are systems that show significant diatropic ring current and are non-aromatic (e.g., cyclopentadiene). There are systems with 4nπ electrons which show diatropic ring current (e.g., phenylalanyl cation, and so forth) Tropicities are indices for aromaticity, not aromaticity. This was formulated as diatropicity being a necessary but not sufficient condition to define aromaticity (see, e.g., ref (71)). The relation between tropicity and (anti)aromaticity is also discussed in ref (8).” We agree with the reviewer that in the deformed aromatic rings, dia- and paratropic ring currents mix up. This is a consequence of mixing the π and σ orbitals which are more inseparable the more deformed the ring is. Thus, it is impossible to fully determine dia- and paratropic ring currents in such rings. However, in our opinion, there is no such thing as “the essence of aromaticity”, and the aromaticity, as non-observable, is nothing but what is provided by a given parameter. Hence, if we use parameter α, we have α-aromaticity, if we use a parameter β, we have β-aromaticity, and if there are some parameters γ_1_, γ_2_, ..., γ_*n*_ of the γ family of parameters, we will call the aromaticity estimated by these parameters as γ_1_-aromaticity, γ_2_-aromaticity, ..., γ_*n*_-aromaticity. To be consistent, basically, we should use the term “NICS_ZZ_-face aromaticity”, which we find too convoluted. From the philosophical point of view, the reviewer’s and our philosophical positions can be assigned to different philosophical systems, which are realism and empiricism, respectively.^72^ The dispute between realism and empiricism is not decidable. Regardless of our philosophical views, we respect the position of the reviewer and generally different philosophical approaches and therefore we do not want to force the adoption of one nomenclature, leaving the reader of this article a choice.

## Supporting Information Available

Total and Gibbs free energies; population factors for all Phe, Tyr, Trp, and His conformers; different NICS indices of all conformers; NICS scans for all conformers; tables of different NICS values; additional plots comparing NICS values of different molecules; and XYZ coordinates of the structures (PDF)
