# Supplementary material for: On Aromaticity of the Aromatic α-Amino Acids and Tuning of the NICS Indices to Find the Aromaticity Order
Source: J Phys Chem A. 2022 May 26;126(22):3433–44. doi: 10.1021/acs.jpca.2c00346 (PMC9189847; doi:10.1021/acs.jpca.2c00346)
Supplement: Supplementary file 1 — jp2c00346_si_001.pdf [file jp2c00346_si_001.pdf]

Supplementary information to:

On Aromaticity of the Aromatic α-Amino Acids and Tuning the NICS Indices to Find the Aromaticity Order

Wojciech M. Dudek, Sławomir Ostrowski and Jan Cz. Dobrowolski*

Institute of Nuclear Chemistry and Technology, 16 Dorodna Street, 03-195 Warsaw, Poland

Contents

No		page
1	**Figure S1.** The $NICS_{zz}$ scans for aromatic rings in the Phe conformers in the gas phase calculated at the B3LYP/D3/aug-cc-pVTZ level.	**S3**
2	**Figure S2.** The $NICS_{zz}$ scans for aromatic rings in the Tyr conformers in the gas phase calculated at the B3LYP/D3/aug-cc-pVTZ level.	**S4**
3	**Figure S3.** The $NICS_{zz}$ scans for aromatic pyrrole rings in the Trp conformers in the gas phase calculated at the B3LYP/D3/aug-cc-pVTZ level.	**S5**
4	**Figure S4.** The $NICS_{zz}$ scans for aromatic phenyl rings in the Trp conformers in the gas phase calculated at the B3LYP/D3/aug-cc-pVTZ level.	**S6**
5	**Figure S5.** The $NICS_{zz}$ scans for aromatic rings in conformers of the His($N^{\varepsilon}H$) tautomer in the gas phase calculated at the B3LYP/D3/aug-cc-pVTZ level.	**S7**
6	**Figure S6.** The $NICS_{zz}$ scans for aromatic rings in conformers of the His($N^{\delta}H$) tautomer in the gas phase calculated at the B3LYP/D3/aug-cc-pVTZ level.	**S8**
7	**Figure S7.** The $NICS_{zz}$ scans for aromatic rings in conformers of the His($N^{\delta}H$) and His($N^{\varepsilon}H$) tautomers and their mixture His in the gas phase estimated based on (a) total energies and (b) Gibbs free energies calculated at the B3LYP/D3/aug-cc-pVTZ level. In the mixture the total content of the His($N^{\delta}H$) tautomer is only 1.9 %, therefore black and red lines representing total population and His($N^{\varepsilon}H$) tautomers overlap.	**S9**
8	**Figure S8.** The population averaged aromaticity indices of rings in the Phe, Tyr, Trp, and His aromatic amino acids (light gray) and selected 6-membered (black) and 5-membered rings (dark gray): (a) and (b) indices taken in points $NICS_{ZZ}(min_{rev})_{pav}$ and $NICS_{ZZ}(0)_{pav}$, respectively. Calculations were done at the B3LYP/D3/aug-cc-pVTZ level.	**S10**
9	**Figure S9.** The $NICS_{zz}$ scans for a series of benzenes monosubstituted with groups of different σ- and π-electron activity.	**S11**
10	**Figure S10.** The linear correlations between differences of the integral aromaticity indices of rings in a series of benzenes monosubstituted with groups of different σ- and π-electron activity, $\Delta(INICS_{ZZ}(tot))= INICS_{ZZ}(benzene) - INICS_{ZZ}(substituted benzene)$, and analogous differences for indices taken in points: (a) $NICS_{ZZ}(1)$ and (b) $NICS_{ZZ}(0)$. Calculations were done at the B3LYP/D3/aug-cc-pVTZ level. Notice that the greater the value of difference the smaller the NICSZZ aromaticity.	**S12**
11	**Table S1.** Energy differences (kcal/mol) and population (%, according to total energies) of the most frequent three conformers in vacuum calculated at the B3LYP/D3/aug-cc-pVTZ level. Data for all conformers found is gathered in S1-S4 of ESI.	**S13**
12	**Table S2.** Total energy, Gibbs free energy and zero-point energy (hartree) and populations determined based on first two kinds of enegy (%) for the Phe conformers assuming T = 298.15 K. Calculations were performed at the B3LYP/D3/aug-cc-pVTZ level.	**S13**
13	**Table S3.** Total energy, Gibbs free energy and zero-point energy (hartree) and populations determined based on first two kinds of enegy (%) for the **Tyr** conformers assuming T = 298.15 K. Calculations were performed at the B3LYP/D3/aug-cc-pVTZ level.	**S14**

14 **Table S4.** Total energy, Gibbs free energy and zero-point energy (hartree) and populations determined based on first two kinds of enegy (%) for the **Trp** conformers assuming T = 298.15 K. Calculations were performed at the B3LYP/D3/aug-cc-pVTZ level. **S15**

15 **Table S5.** Total energy, Gibbs free energy and zero-point energy (hartree) and populations determined based on first two kinds of enegy (%) for the conformers of the His(N^{ε}H) tautomer assuming T = 298.15 K. Calculations were performed at the B3LYP/D3/aug-cc-pVTZ level. **S16**

16 **Table S6.** Total energy, Gibbs free energy and zero-point energy (hartree) and populations determined based on first two kinds of enegy (%) for the conformers of the His(N^{δ}H) tautomer assuming T = 298.15 K. Calculations were performed at the B3LYP/D3/aug-cc-pVTZ level. **S17**

17 **Table S7.** Total energy, Gibbs free energy and zero-point energy (hartree) and populations determined based on first two kinds of enegy (%) for the conformers of the His(N^{δ}H) and His(N^{ε}H) tautomers assuming T = 298.15 K. Calculations were performed at the B3LYP/D3/aug-cc-pVTZ level. **S18**

18 **Table S8.** Different NICS aromaticity indices of rings in Phe, Tyr, Trp, and His aromatic amino acids and their three the most populated conformers calculated at the B3LYP/D3/aug-cc-pVTZ level. min, max, and *s* denote the minimum and maximum value of the population and the standard deviation. Population of the three most frequent conformers were taken according to total energies, T = 298.15 K, or Gibbs free energies, T=298.15 K. **S20**

19 **Table S9.** The $NICS_{zz}$ values (ppm) for Phe and Tyr conformers calculated at the B3LYP/D3/aug-cc-pVTZ level **S21**

20 **Table S10.** The $NICS_{zz}$ values (ppm) for Trp pyrrole and phenyl rings in different conformers calculated at the B3LYP/D3/aug-cc-pVTZ level **S22**

21 **Table S11.** The $NICS_{zz}$ values (ppm) for His (N^{ε}H) and His (N^{δ}H) conformers calculated at the B3LYP/D3/aug-cc-pVTZ level **S23**

22 **Table S12.** The $NICS_{zz}$ values (ppm) for both (N^{ε}H) and His (N^{δ}H) conformers calculated at the B3LYP/D3/aug-cc-pVTZ level **S24**

23 **Table S13.** Different integral INICS aromaticity indices of rings in Phe, Tyr, Trp, and His aromatic amino acids and their three the most populated conformers calculated at the B3LYP/D3/aug-cc-pVTZ level. Population of the three most frequent conformers were taken according to total energies, T = 298.15 K, or Gibbs free energies, T=298.15 K.The integration range was from -9.8 to 0.0 Å, and from 0.0 to 9.8 Å for $INICS_{zz}$(obv) and $INICS_{zz}$(rev), respectively, while $INICS_{zz}$(tot) = $INICS_{zz}$(obv) + $INICS_{zz}$(rev). **S25**

24 **Table S14.** The integral $NICS_{zz}$ values (ppm·Å) for Phe and Tyr conformers calculated at the B3LYP/D3/aug-cc-pVTZ level **S26**

25 **Table S15.** The integral $NICS_{zz}$ values (ppm·Å) for Trp phenyl and pyrrole rings in various conformers calculated at the B3LYP/D3/aug-cc-pVTZ level **S27**

26 **Table S16.** The integral $NICS_{zz}$ values (ppm·Å) for His conformers calculated at the B3LYP/D3/aug-cc-pVTZ level **S28**

27 **Table S17.** The NICSZZ values (ppm) for both (NεH) and (NδH) conformers calculated at the B3LYP/D3/aug-cc-pVTZ level **S29**

28 **Table S18.** Various $NICS_{zz}$ indices for a series of benzenes monosubstituted with groups of different σ- and π-electron activity. [64-67] The values are ordered according to decreasing $INICS_{zz}$(tot) index. The differences are are taken with respect to the adequate values for the unsubstituted benzene. **S30**

29 **Table S19.** Tables with the optimized Cartesian coordinates Phe calculated at the B3LYP/D3/aug-cc-pVTZ . **S31**

30 **Table S20.** Tables with the optimized Cartesian coordinates Tyr conformers calculated at the B3LYP/D3/aug-cc-pVTZ . **S35**

31 **Table S21.** Tables with the optimized Cartesian coordinates Trp conformers calculated at the B3LYP/D3/aug-cc-pVTZ. **S42**

32 **Table S22.** Tables with the optimized Cartesian coordinates His(N^{ε}H) tautomer conformers calculated at the B3LYP/D3/aug-cc-pVTZ . **S49**

33 **Table S23.** Tables with the optimized Cartesian coordinates His(N^{δ}H) tautomer conformers calculated at the B3LYP/D3/aug-cc-pVTZ. **S53**

34 **Table S24.** Tables with the distance of atoms in the rings and HOMA index for: Phe, Tyr, Trp and His **S56**

35 **Table S25.** The HOMA index of some model rings calculated at the B3LYP/D3/aug-cc-pVTZ level. **S63**

36 **Table S26.** The population averaged HOMApav index for: Phe, Tyr, Trp and His conformers and the HOMA index of some model rings calculated at the B3LYP/D3/aug-cc-pVTZ level. **S63**

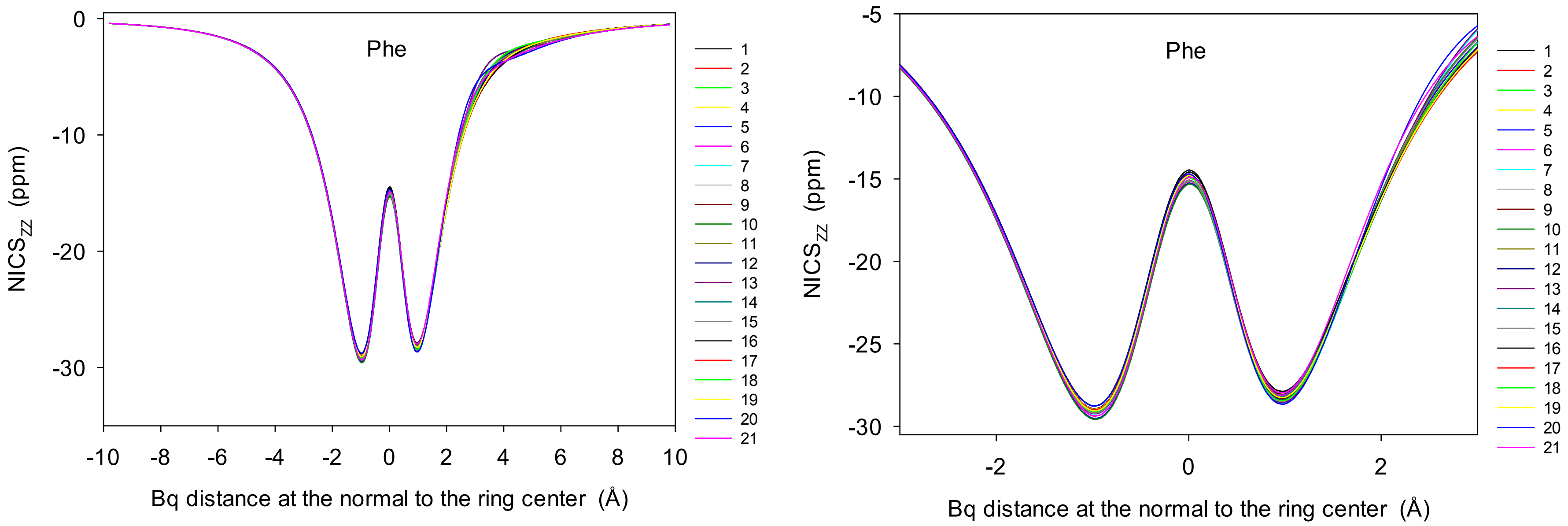

Figure S1. The $NICS_{zz}$ scans for aromatic rings in the Phe conformers in the gas phase calculated at the B3LYP/D3/aug-cc-pVTZ level.

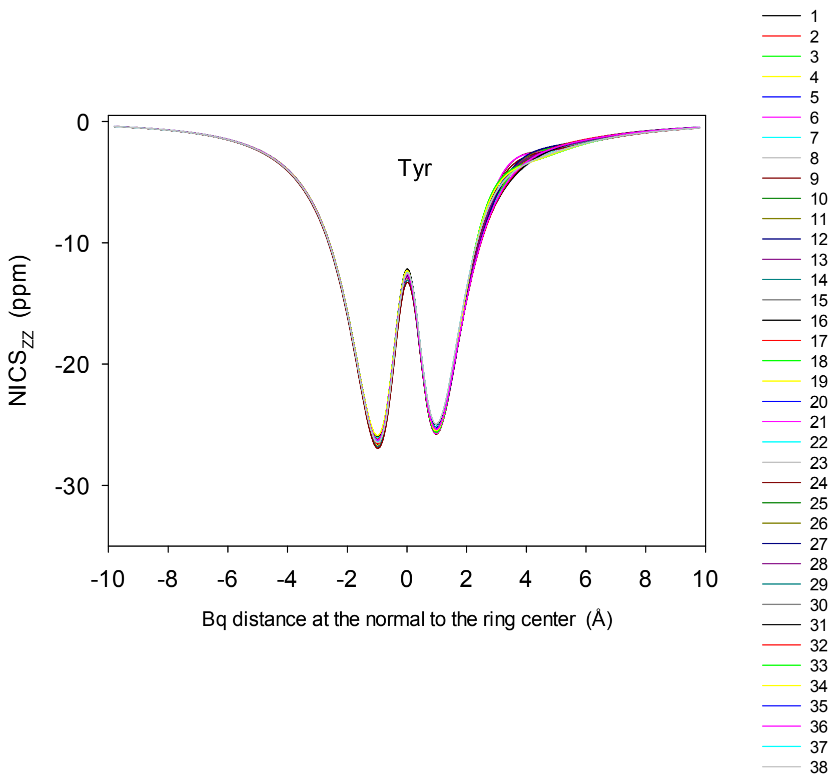

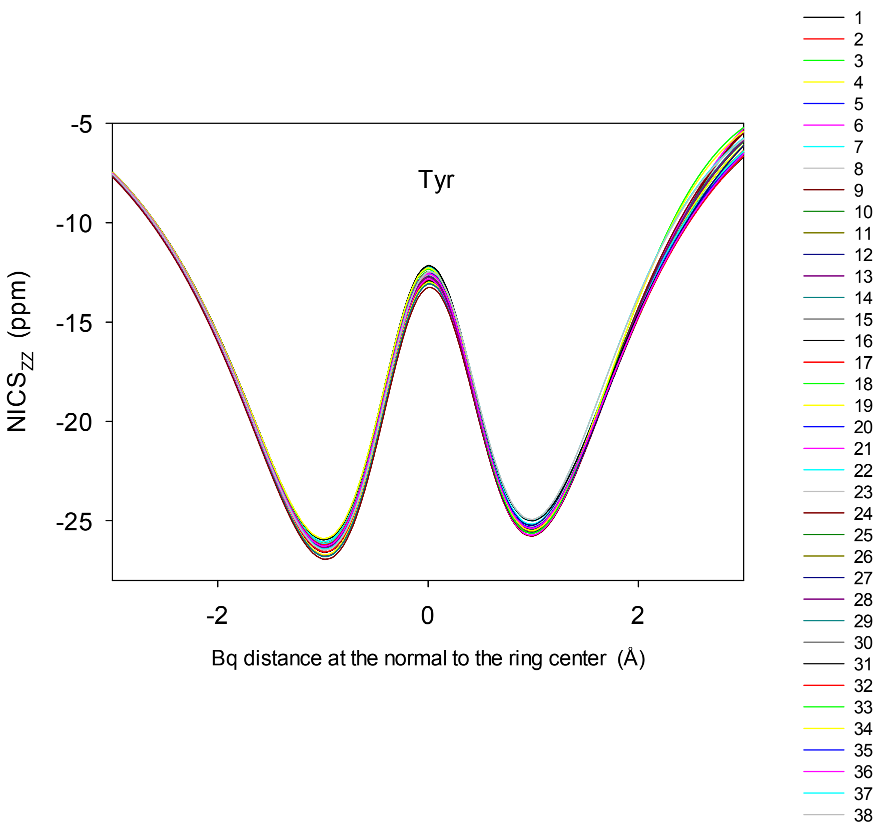

Figure S2. The NICS$_{ZZ}$ scans for aromatic rings in the Tyr conformers in the gas phase calculated at the B3LYP/D3/aug-cc-pVTZ level.

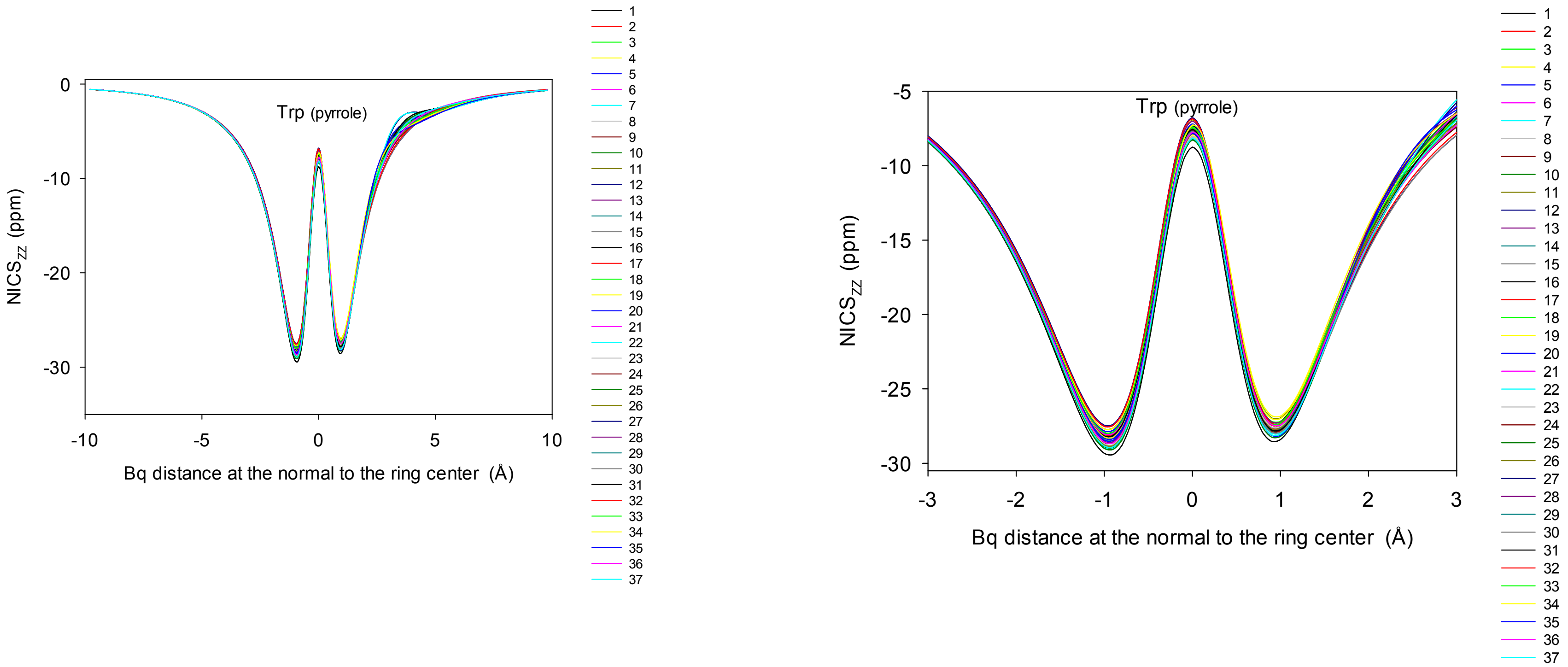

Figure S3. The NICS$_{ZZ}$ scans for aromatic pyrrole rings in the Trp conformers in the gas phase calculated at the B3LYP/D3/aug-cc-pVTZ level.

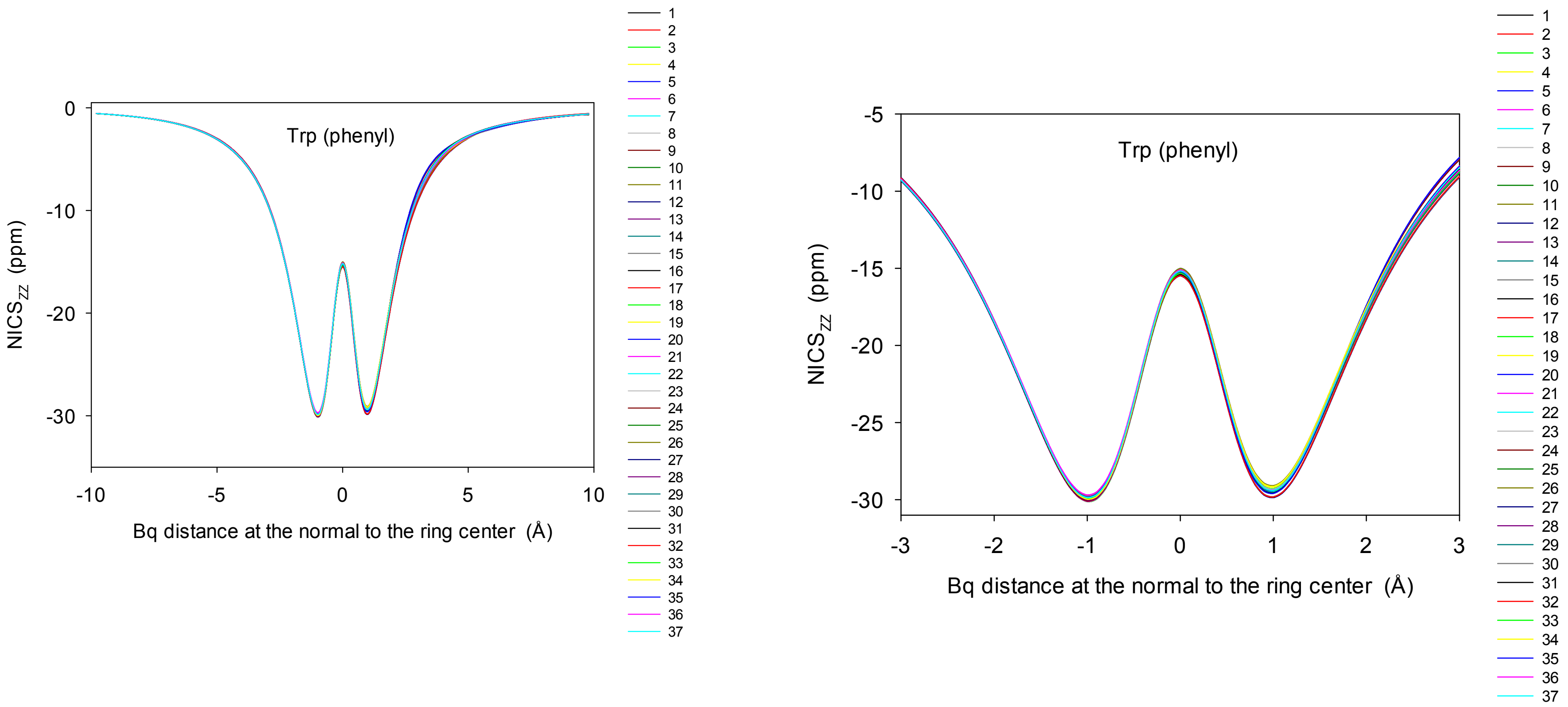

Figure S4. The NICS$_{ZZ}$ scans for aromatic phenyl rings in the Trp conformers in the gas phase calculated at the B3LYP/D3/aug-cc-pVTZ level.

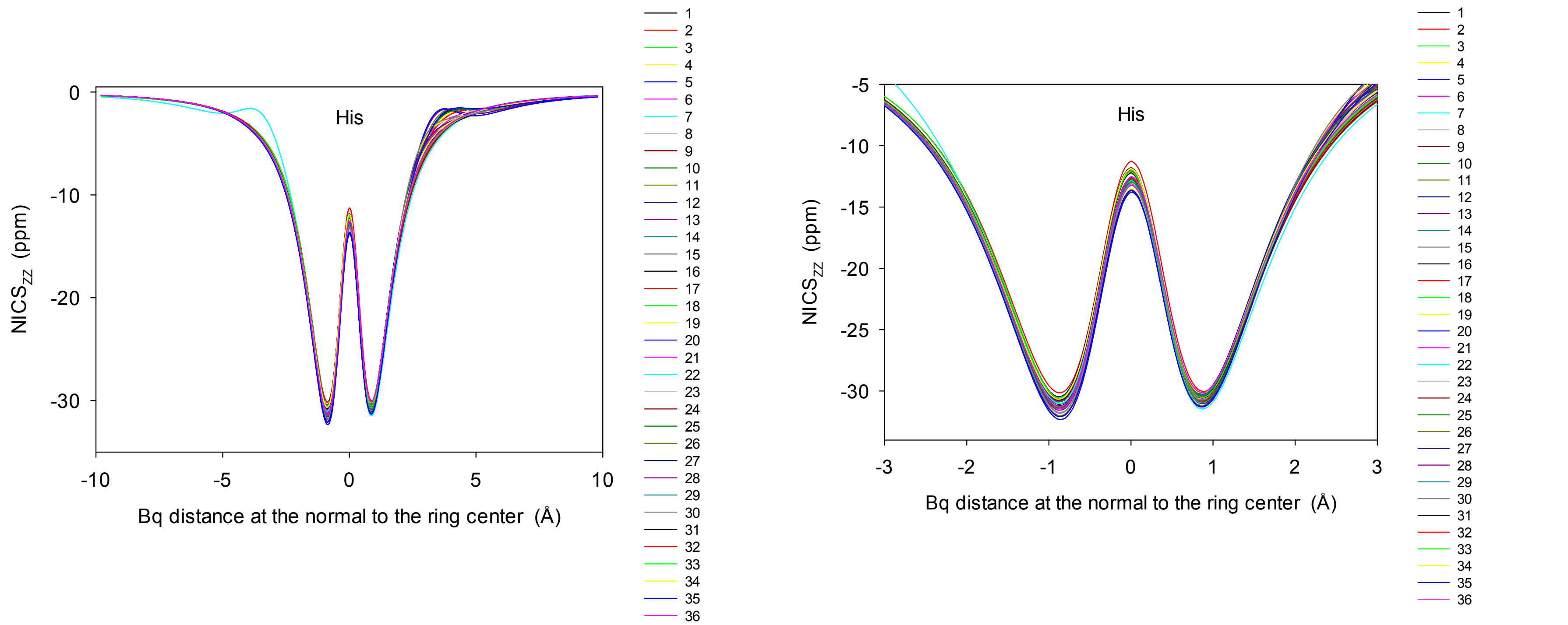

Figure S5. The NICS$_{ZZ}$ scans for aromatic rings in conformers of the His(N$^{\varepsilon}$H) tautomer in the gas phase calculated at the B3LYP/D3/aug-cc-pVTZ level.

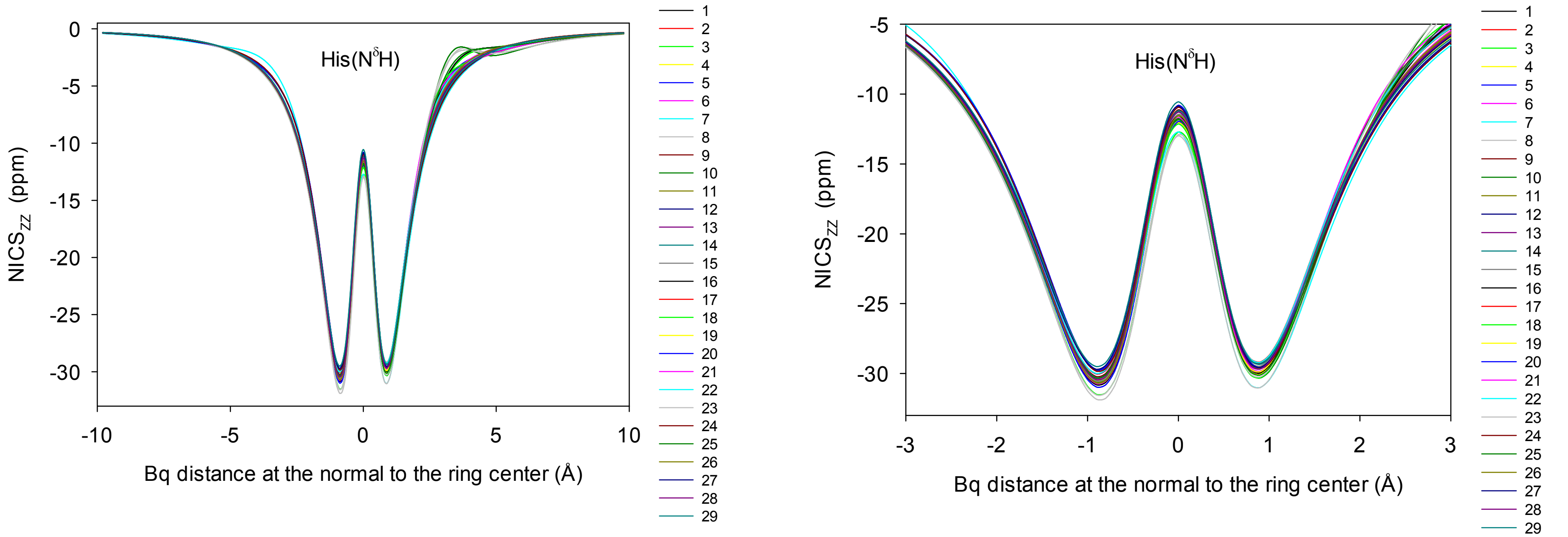

Figure S6. The NICS$_{zz}$ scans for aromatic rings in conformers of the His(N$^{\delta}$H) tautomer in the gas phase calculated at the B3LYP/D3/aug-cc-pVTZ level.

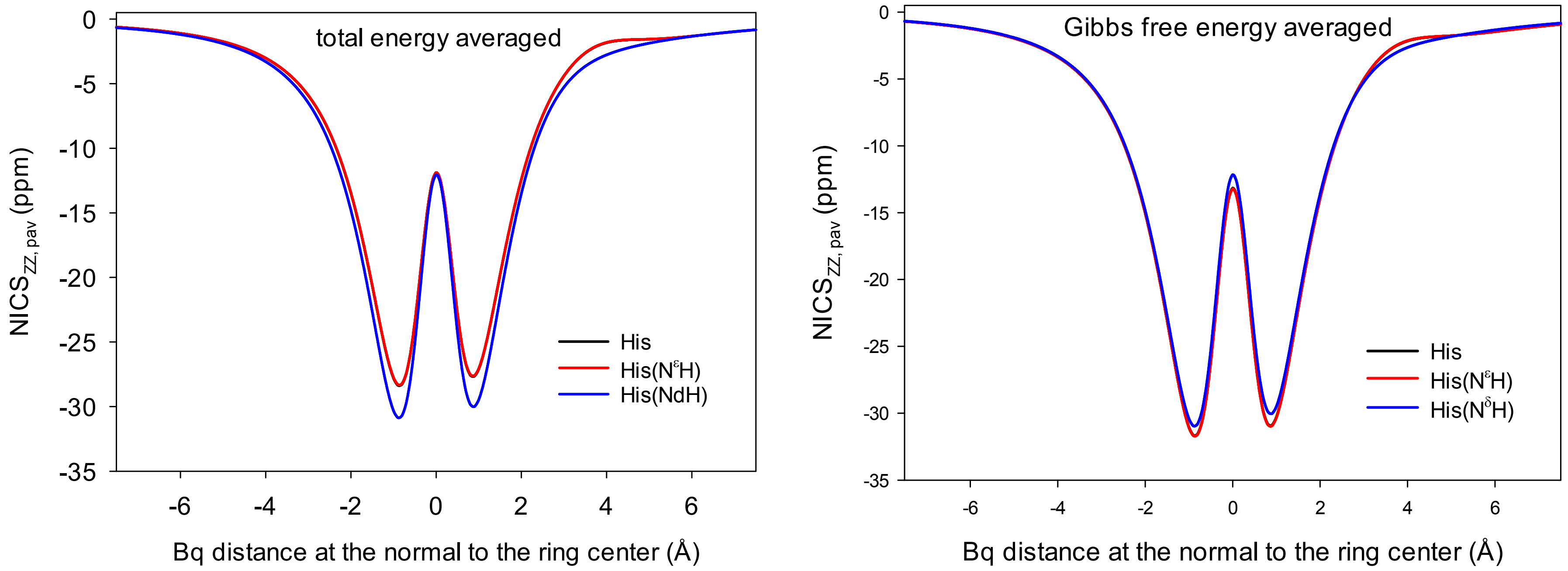

(a) (b)

Figure S7. The NICS$_{ZZ}$ scans for aromatic rings in conformers of the His(N^{δ}H) and His(N^{ε}H) tautomers and their mixture His in the gas phase estimated based on (a) total energies and (b) Gibbs free energies calculated at the B3LYP/D3/aug-cc-pVTZ level. In the mixture the total content of the His(N^{δ}H) tautomer is only 1.9 %, therefore black and red lines representing total population and His(N^{ε}H) tautomers overlap.

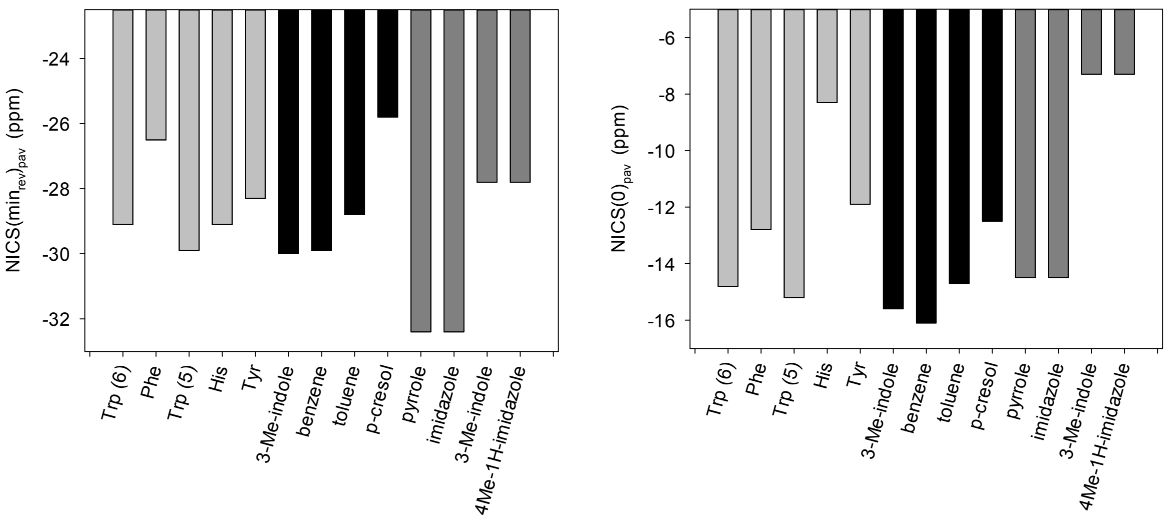

(a) (b)

Figure S8. The population averaged aromaticity indices of rings in the Phe, Tyr, Trp, and His aromatic amino acids (light gray) and selected 6-membered (black) and 5-membered rings (dark gray): (a) and (b) indices taken in points $NICS_{ZZ}(min_{resv})_{pav}$ and $NICS_{ZZ}(0)_{pav}$, respectively. Calculations were done at the B3LYP/D3/aug-cc-pVTZ level.

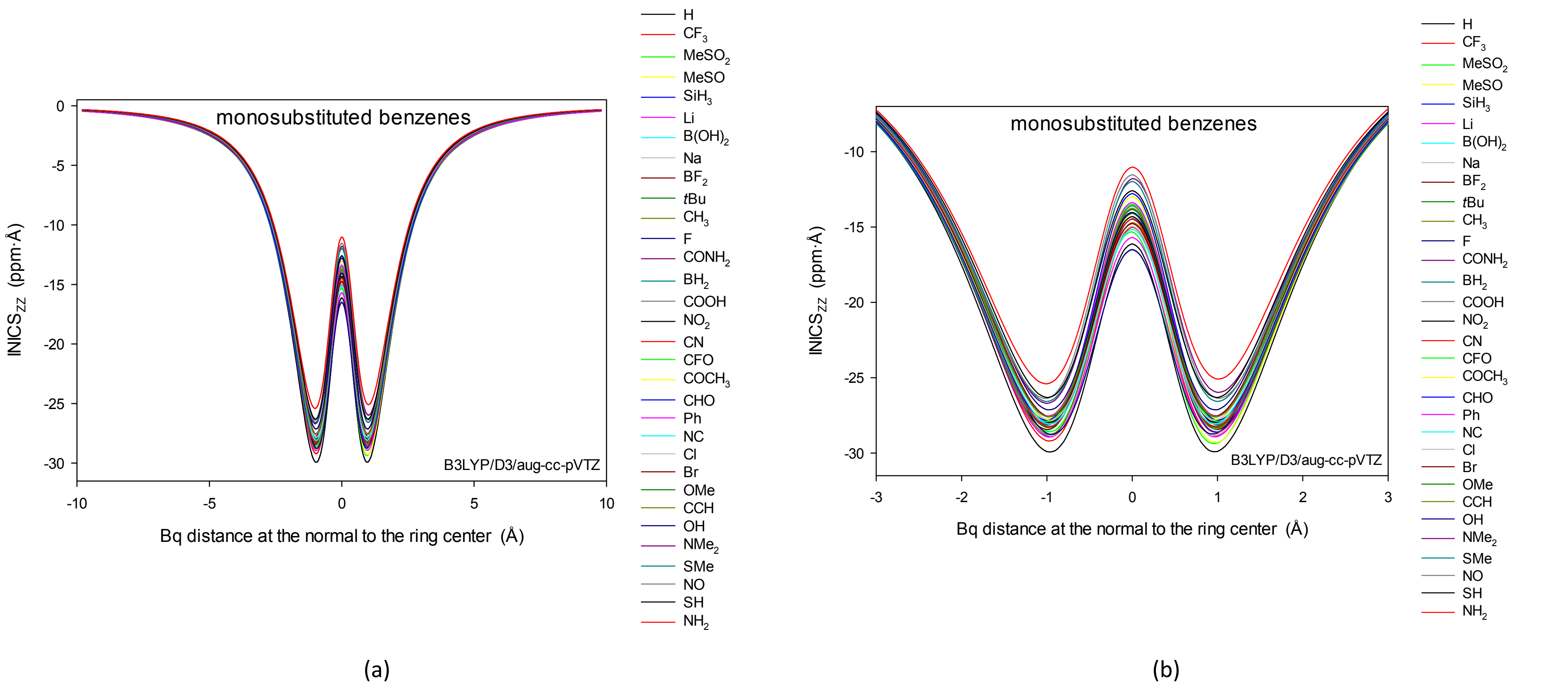

Figure S9. The $NICS_{ZZ}$ scans for a series of benzenes monosubstituted with groups of different σ- and π-electron activity.

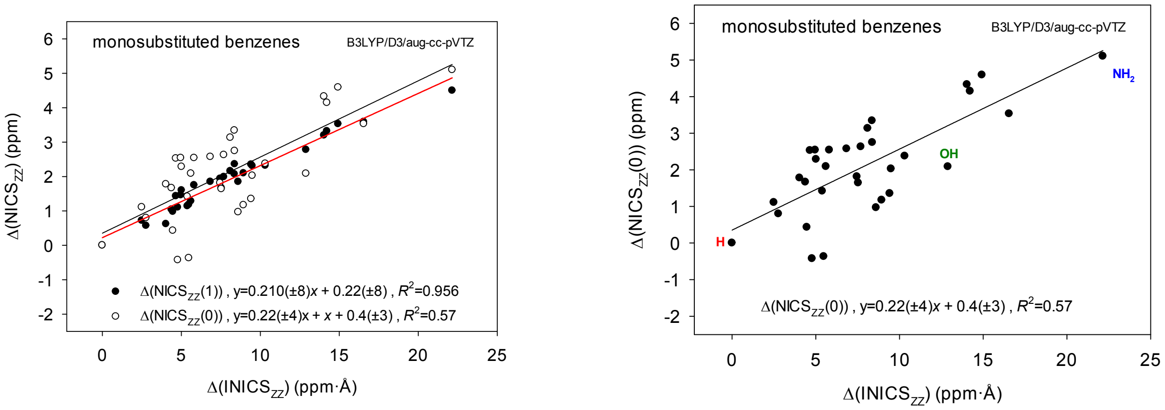

(a) (b)

Figure S10. The linear correlations between differences of the integral aromaticity indices of rings in a series of benzenes monosubstituted with groups of different σ- and π-electron activity, Δ($INICS_{ZZ}$(tot))= $INICS_{ZZ}$(benzene) - $INICS_{ZZ}$(substituted benzene), and analogous differences for indices taken in points: (a) $NICS_{ZZ}(1)$ and (b) $NICS_{ZZ}(0)$. Calculations were done at the B3LYP/D3/aug-cc-pVTZ level. Notice that the greater the value of difference the smaller the NICSZZ aromaticity.

ENERGIES

Table S1. Energy differences (kcal/mol) and population (%, according to total energies) of the most frequent three conformers in vacuum calculated at the B3LYP/D3/aug-cc-pVTZ level. Data for all conformers found is gathered in S1-S4 of ESI.

Energy differences	conformer	Phe	Tyr	Trp	His
Population according to total energies	1	50.53	40.25	58.95	81.54
	2	18.51	22.90	10.47	11.57
	3	9.81	8.96	9.71	1.96
Total energy	1	0.000	0.000	0.000	0.000
	2	0.594	1.020	0.330	1.160
	3	0.970	1.070	0.890	2.210
Zero point energy 0K	1	0.000	0.000	0.000	0.000
	2	0.592	1.040	0.320	1.020
	3	0.870	1.050	0.880	2.180
Gibbs free energy 298.15K	1	0.000	0.000	0.000	0.000
	2	0.173	0.690	0.290	1.010
	3	0.085	1.120	0.420	1.690

Table S2. Total energy, Gibbs free energy and zero-point energy (hartree) and populations determined based on first two kinds of enegy (%) for the Phe conformers assuming T = 298.15 K. Calculations were performed at the B3LYP/D3/aug-cc-pVTZ level.

molecule	pop_E(%)	pop_{G298} (%)	ΔE(kcal)	ΔG_{298} (kcal)	E(hartree)	G_{298} (hartree)	E_{ZPE}(hartree)
1	50.53	19.06	0.00	0.00	-555.035733	-554.882818	-554.845546
2	18.51	14.24	0.59	0.17	-555.034785	-554.882543	-554.844776
3	9.81	16.50	0.97	0.09	-555.034187	-554.882682	-554.844424
4	2.56	5.94	1.77	0.69	-555.032920	-554.881718	-554.843517
5	9.27	14.87	1.00	0.15	-555.034134	-554.882584	-554.844553
6	1.05	5.68	2.29	0.72	-555.032078	-554.881676	-554.842860
7	2.21	8.30	1.85	0.49	-555.032780	-554.882034	-554.843375
8	1.09	3.04	2.27	1.09	-555.032114	-554.881086	-554.842833
9	0.48	1.69	2.75	1.43	-555.031348	-554.880533	-554.842019
10	2.66	3.99	1.74	0.92	-555.032957	-554.881344	-554.843210
11	0.47	1.51	2.76	1.50	-555.031329	-554.880428	-554.841944
12	0.48	1.54	2.75	1.49	-555.031347	-554.880444	-554.841948
13	0.44	1.51	2.81	1.50	-555.031260	-554.880426	-554.841912
14	0.13	1.42	3.53	1.54	-555.030112	-554.880367	-554.840914
15	0.11	0.18	3.61	2.74	-555.029978	-554.878446	-554.840336
16	0.06	0.13	3.99	2.95	-555.029378	-554.878121	-554.839586
17	0.04	0.10	4.23	3.11	-555.028994	-554.877869	-554.839656
18	0.05	0.20	4.07	2.70	-555.029254	-554.878513	-554.840049
19	0.01	0.05	5.07	3.53	-555.027656	-554.877198	-554.838375
20	0.02	0.06	4.72	3.45	-555.028215	-554.877324	-554.838970
21	0.00	0.00	6.30	5.29	-555.025686	-554.874394	-554.836033

Table S3. Total energy, Gibbs free energy and zero-point energy (hartree) and populations determined based on first two kinds of enegy (%) for the **Tyr** conformers assuming T = 298.15 K. Calculations were performed at the B3LYP/D3/aug-cc-pVTZ level.

molecule	pop_E(%)	pop_{G298} (%)	ΔE(kcal)	$ΔG_{298}$ (kcal)	E(hartree)	G_{298} (hartree)	E_{ZPE}(hartree)
1	40.25	15.77	0.00	0.00	-630.292012	-630.136461	-630.097764
2	22.90	9.67	0.33	0.29	-630.291480	-630.135999	-630.097263
3	8.96	7.70	0.89	0.42	-630.290595	-630.135785	-630.096548
4	4.90	9.04	1.25	0.33	-630.290027	-630.135936	-630.096411
5	4.86	8.93	1.25	0.34	-630.290018	-630.135924	-630.096407
6	4.23	8.20	1.33	0.39	-630.289888	-630.135844	-630.096104
7	3.87	7.85	1.39	0.41	-630.289804	-630.135803	-630.096027
8	1.44	2.31	1.97	1.14	-630.288873	-630.134649	-630.095072
9	1.40	2.26	1.99	1.15	-630.288844	-630.134627	-630.095049
10	1.20	3.05	2.08	0.97	-630.288700	-630.134912	-630.095262
11	1.10	5.21	2.13	0.66	-630.288619	-630.135417	-630.095181
12	1.05	4.49	2.16	0.74	-630.288577	-630.135275	-630.095144
13	0.56	3.10	2.53	0.96	-630.287974	-630.134928	-630.094710
14	0.53	1.67	2.56	1.33	-630.287931	-630.134342	-630.094614
15	0.47	2.68	2.63	1.05	-630.287822	-630.134788	-630.094569
16	0.46	1.42	2.65	1.43	-630.287794	-630.134188	-630.094481
17	0.24	0.90	3.03	1.70	-630.287189	-630.133759	-630.093765
18	0.23	0.85	3.05	1.73	-630.287148	-630.133708	-630.093724
19	0.23	0.81	3.07	1.76	-630.287126	-630.133655	-630.093716
20	0.21	0.75	3.11	1.80	-630.287053	-630.133590	-630.093647
21	0.20	0.80	3.13	1.77	-630.287021	-630.133644	-630.093654
22	0.20	0.81	3.15	1.76	-630.287000	-630.133663	-630.093657
23	0.11	0.19	3.50	2.62	-630.286434	-630.132286	-630.092916
24	0.08	0.14	3.66	2.79	-630.286183	-630.132009	-630.092486
25	0.06	0.69	3.81	1.85	-630.285943	-630.133510	-630.092701
26	0.05	0.10	3.92	2.99	-630.285764	-630.131690	-630.092095
27	0.04	0.08	4.03	3.13	-630.285585	-630.131479	-630.091717
28	0.03	0.06	4.36	3.33	-630.285062	-630.131160	-630.091224
29	0.03	0.12	4.36	2.89	-630.285060	-630.131849	-630.091839
30	0.02	0.12	4.37	2.90	-630.285042	-630.131837	-630.091824
31	0.02	0.06	4.39	3.26	-630.285013	-630.131265	-630.091642
32	0.02	0.05	4.59	3.43	-630.284701	-630.130987	-630.091339
33	0.01	0.04	4.81	3.47	-630.284340	-630.130934	-630.091050
34	0.01	0.03	5.00	3.64	-630.284040	-630.130668	-630.090770
35	0.00	0.03	5.33	3.78	-630.283514	-630.130434	-630.090209
36	0.00	0.03	5.36	3.80	-630.283463	-630.130410	-630.090171
37	0.00	0.00	6.33	5.61	-630.281919	-630.127516	-630.088135
38	0.00	0.00	6.44	5.83	-630.281743	-630.127169	-630.087936

Table S4. Total energy, Gibbs free energy and zero-point energy (hartree) and populations determined based on first two kinds of enegy (%) for the **Trp** conformers assuming T = 298.15 K. Calculations were performed at the B3LYP/D3/aug-cc-pVTZ level.

molecule	pop_E(%)	pop_{G298} (%)	ΔE(kcal)	ΔG_{298} (kcal)	E(hartree)	G_{298} (hartree)	E_{ZPE}(hartree)
1	58.95	35.39	0.00	0.00	-686.658685	-686.479326	-686.439382
2	10.47	10.97	1.02	0.69	-686.657054	-686.478221	-686.437879
3	9.71	5.32	1.07	1.12	-686.656984	-686.477538	-686.437714
4	4.07	3.29	1.58	1.41	-686.656163	-686.477085	-686.436908
5	3.09	9.53	1.75	0.78	-686.655904	-686.478089	-686.437048
6	2.82	4.73	1.80	1.19	-686.655817	-686.477428	-686.437074
7	2.79	5.00	1.81	1.16	-686.655807	-686.477480	-686.437032
8	1.54	2.70	2.16	1.52	-686.655247	-686.476900	-686.436264
9	1.44	1.51	2.20	1.87	-686.655186	-686.476349	-686.436248
10	0.98	2.02	2.43	1.70	-686.654818	-686.476624	-686.435916
11	0.91	3.30	2.47	1.40	-686.654755	-686.477089	-686.436262
12	0.60	4.94	2.72	1.17	-686.654351	-686.477468	-686.435868
13	0.56	1.09	2.75	2.06	-686.654295	-686.476041	-686.435661
14	0.33	1.66	3.08	1.81	-686.653784	-686.476441	-686.435427
15	0.32	0.88	3.09	2.19	-686.653765	-686.475842	-686.435163
16	0.27	2.69	3.18	1.52	-686.653619	-686.476896	-686.435304
17	0.14	0.89	3.56	2.18	-686.653014	-686.475850	-686.434612
18	0.14	0.32	3.56	2.79	-686.653011	-686.474887	-686.434462
19	0.14	0.74	3.59	2.29	-686.652968	-686.475681	-686.434519
20	0.12	0.22	3.65	3.00	-686.652872	-686.474546	-686.434281
21	0.12	0.61	3.66	2.40	-686.652846	-686.475498	-686.434356
22	0.10	0.70	3.79	2.32	-686.652644	-686.475628	-686.434245
23	0.07	0.22	3.96	3.00	-686.652377	-686.474538	-686.433792
24	0.07	0.13	4.01	3.30	-686.652294	-686.474066	-686.433313
25	0.07	0.24	4.02	2.96	-686.652278	-686.474606	-686.433747
26	0.07	0.24	4.02	2.95	-686.652271	-686.474617	-686.433683
27	0.03	0.04	4.49	3.98	-686.651530	-686.472989	-686.432532
28	0.02	0.10	4.60	3.50	-686.651354	-686.473753	-686.432947
29	0.02	0.10	4.72	3.47	-686.651164	-686.473801	-686.432773
30	0.01	0.24	5.00	2.96	-686.650714	-686.474602	-686.432225
31	0.01	0.08	5.03	3.61	-686.650673	-686.473569	-686.432392
32	0.01	0.03	5.11	4.25	-686.650542	-686.472552	-686.431960
33	0.00	0.04	5.89	4.01	-686.649293	-686.472943	-686.431197
34	0.00	0.00	5.93	5.92	-686.649238	-686.469891	-686.430107
35	0.00	0.03	6.10	4.11	-686.648971	-686.472781	-686.430676
36	0.00	0.01	6.45	5.20	-686.648414	-686.471044	-686.430034
37	0.00	0.01	6.57	4.97	-686.648220	-686.471411	-686.429941

Table S5. Total energy, Gibbs free energy and zero-point energy (hartree) and populations determined based on first two kinds of enegy (%) for the conformers of the His(N$^{\epsilon}$H) tautomer assuming T = 298.15 K. Calculations were performed at the B3LYP/D3/aug-cc-pVTZ level.

molecule	pop$_E$(%)	pop$_{G298}$ (%)	ΔE(kcal)	ΔG$_{298}$ (kcal)	E(hartree)	G$_{298}$ (hartree)	E$_{ZPE}$(hartree)
1	83.12	74.81	0.00	0.00	-549.010190	-548.885652	-548.849177
2	11.79	13.58	1.16	1.01	-549.008346	-548.884041	-548.847717
3	2.00	4.35	2.21	1.69	-549.006671	-548.882965	-548.845883
4	1.95	1.17	2.22	2.46	-549.006646	-548.881725	-548.845613
5	0.42	1.30	3.13	2.40	-549.005195	-548.881822	-548.844876
6	0.22	0.99	3.52	2.56	-549.004574	-548.881567	-548.844407
7	0.10	0.43	4.01	3.06	-549.003799	-548.880773	-548.843407
8	0.06	0.44	4.24	3.05	-549.003433	-548.880792	-548.843369
9	0.05	0.72	4.38	2.75	-549.003208	-548.881267	-548.843099
10	0.04	0.46	4.54	3.01	-549.002912	-548.880821	-548.842977
11	0.04	0.45	4.57	3.03	-549.002948	-548.880849	-548.843016
12	0.03	0.09	4.66	3.97	-549.002760	-548.879325	-548.842454
13	0.03	0.10	4.76	3.92	-549.002597	-548.879402	-548.841995
14	0.02	0.28	4.81	3.31	-549.002343	-548.879631	-548.842072
15	0.02	0.02	4.88	4.91	-549.002533	-548.880379	-548.842441
16	0.02	0.13	4.92	3.78	-549.002416	-548.877823	-548.841805
17	0.01	0.14	5.18	3.74	-549.001412	-548.879554	-548.841454
18	0.01	0.10	5.21	3.94	-549.001803	-548.878838	-548.841605
19	0.01	0.07	5.23	4.13	-549.001228	-548.878573	-548.841130
20	0.01	0.05	5.26	4.28	-549.001856	-548.879064	-548.841759
21	0.01	0.02	5.40	4.84	-549.001893	-548.879368	-548.841789
22	0.01	0.12	5.51	3.83	-549.001933	-548.879697	-548.841847
23	0.01	0.04	5.62	4.44	-549.001582	-548.877932	-548.840940
24	0.00	0.03	5.83	4.56	-548.999202	-548.877617	-548.839395
25	0.00	0.02	5.90	4.91	-549.000383	-548.878388	-548.840259
26	0.00	0.03	6.15	4.56	-548.999411	-548.876006	-548.839103
27	0.00	0.02	6.22	4.87	-548.999905	-548.876557	-548.839660
28	0.00	0.01	6.23	5.17	-549.000795	-548.877835	-548.840606
29	0.00	0.00	6.45	5.71	-549.000273	-548.877893	-548.840314
30	0.00	0.02	6.62	5.02	-548.999433	-548.876344	-548.839218
31	0.00	0.00	6.75	5.84	-549.000266	-548.877408	-548.840232
32	0.00	0.00	6.76	6.05	-549.000898	-548.878392	-548.840844
33	0.00	0.02	6.89	5.04	-548.999638	-548.877654	-548.839852
34	0.00	0.00	7.95	6.52	-548.997250	-548.874292	-548.837255
35	0.00	0.00	8.12	7.13	-548.996333	-548.873449	-548.836311
36	0.00	0.00	8.70	7.66	-548.997527	-548.875269	-548.837510

Table S6. Total energy, Gibbs free energy and zero-point energy (hartree) and populations determined based on first two kinds of enegy (%) for the conformers of the His(N$^{\delta}$H) tautomer assuming T = 298.15 K. Calculations were performed at the B3LYP/D3/aug-cc-pVTZ level.

molecule	pop_E(%)	pop_{G298} (%)	ΔE(kcal)	$ΔG_{298}$ (kcal)	E(hartree)	G_{298} (hartree)	E_{ZPE}(hartree)
1	52.46	35.00	0.00	0.00	-549.006031	-548.882059	-548.845374
2	17.80	12.92	0.64	0.59	-549.005011	-548.881118	-548.844434
3	11.41	25.91	0.90	0.18	-549.004591	-548.881775	-548.844163
4	7.82	10.64	1.13	0.71	-549.004234	-548.880935	-548.843602
5	5.50	4.35	1.34	1.24	-549.003902	-548.880090	-548.843442
6	1.20	1.22	2.24	1.99	-549.002465	-548.878886	-548.842047
7	1.11	1.29	2.29	1.96	-549.002387	-548.878939	-548.841912
8	0.68	2.88	2.57	1.48	-549.001933	-548.879700	-548.841846
9	0.53	1.21	2.72	1.99	-549.001698	-548.878885	-548.841587
10	0.39	1.14	2.91	2.03	-549.001395	-548.878823	-548.841263
11	0.22	0.23	3.23	2.99	-549.000878	-548.877297	-548.840447
12	0.20	0.35	3.30	2.73	-549.000768	-548.877711	-548.840383
13	0.12	0.38	3.61	2.68	-549.000284	-548.877791	-548.840349
14	0.12	0.40	3.60	2.66	-549.000300	-548.877826	-548.840252
15	0.12	0.39	3.60	2.67	-549.000287	-548.877810	-548.840178
16	0.10	0.40	3.71	2.65	-549.000121	-548.877835	-548.839999
17	0.06	0.85	4.05	2.21	-548.999570	-548.878545	-548.839784
18	0.04	0.03	4.33	4.18	-548.999133	-548.875396	-548.838700
19	0.02	0.05	4.54	3.91	-548.998794	-548.875827	-548.838596
20	0.03	0.05	4.46	3.84	-548.998922	-548.875943	-548.838440
21	0.04	0.01	4.29	4.82	-548.999196	-548.874382	-548.838397
22	0.01	0.20	4.91	3.05	-548.998210	-548.877191	-548.838352
23	0.01	0.02	5.04	4.34	-548.997999	-548.875149	-548.837957
24	0.01	0.04	5.22	4.06	-548.997720	-548.875591	-548.837789
25	0.01	0.04	5.14	3.95	-548.997834	-548.875767	-548.837697
26	0.00	0.01	6.02	5.12	-548.996434	-548.873898	-548.836438
27	0.00	0.00	6.21	5.60	-548.996128	-548.873133	-548.836008
28	0.00	0.00	6.48	6.03	-548.995700	-548.872443	-548.835200
29	0.00	0.00	6.76	6.50	-548.995260	-548.871707	-548.834735

Table S7. Total energy, Gibbs free energy and zero-point energy (hartree) and populations determined based on first two kinds of enegy (%) for the conformers of the His(N$^{\delta}$H) and His(N$^{\varepsilon}$H) tautomers assuming T = 298.15 K. Calculations were performed at the B3LYP/D3/aug-cc-pVTZ level.

molecule	pop$_E$(%)	pop$_{G298}$ (%)	ΔE(kcal)	ΔG$_{298}$ (kcal)	E(hartree)	G$_{298}$ (hartree)	E$_{ZPE}$(hartree)
1	81.54	71.41	0.00	0.00	-549.010190	-548.885652	-548.849177
2	11.57	12.97	1.16	1.01	-549.008346	-548.884041	-548.847717
3	1.96	4.15	2.21	1.69	-549.006671	-548.882965	-548.845883
4	1.91	1.12	2.22	2.46	-549.006646	-548.881725	-548.845613
5	1.00	1.59	2.61	2.25	-549.006031	-548.882059	-548.845374
6	0.41	1.24	3.13	2.40	-549.005195	-548.881822	-548.844876
7	0.34	0.59	3.25	2.85	-549.005011	-548.881118	-548.844434
8	0.22	1.18	3.51	2.43	-549.004591	-548.881775	-548.844163
9	0.21	0.94	3.52	2.56	-549.004574	-548.881567	-548.844407
10	0.15	0.48	3.74	2.96	-549.004234	-548.880935	-548.843602
11	0.10	0.20	3.95	3.49	-549.003902	-548.880090	-548.843442
12	0.09	0.41	4.01	3.06	-549.003799	-548.880773	-548.843407
13	0.06	0.42	4.24	3.05	-549.003433	-548.880792	-548.843369
14	0.05	0.69	4.38	2.75	-549.003208	-548.881267	-548.843099
15	0.04	0.44	4.54	3.01	-549.002948	-548.880849	-548.843016
16	0.04	0.43	4.57	3.03	-549.002912	-548.880821	-548.842977
17	0.03	0.09	4.66	3.97	-549.002760	-548.879325	-548.842454
18	0.03	0.10	4.76	3.92	-549.002597	-548.879402	-548.841995
19	0.02	0.27	4.81	3.31	-549.002532	-548.880379	-548.842441
20	0.02	0.06	4.85	4.25	-549.002465	-548.878886	-548.842047
21	0.02	0.02	4.88	4.91	-549.002416	-548.877823	-548.841805
22	0.02	0.06	4.90	4.21	-549.002387	-548.878939	-548.841912
23	0.02	0.12	4.92	3.78	-549.002343	-548.879631	-548.842072
24	0.01	0.13	5.18	3.73	-549.001933	-548.879700	-548.841846
25	0.01	0.13	5.18	3.74	-549.001933	-548.879697	-548.841847
26	0.01	0.09	5.21	3.94	-549.001893	-548.879368	-548.841789
27	0.01	0.07	5.23	4.13	-549.001856	-548.879064	-548.841759
28	0.01	0.05	5.26	4.28	-549.001802	-548.878838	-548.841605
29	0.01	0.06	5.33	4.25	-549.001698	-548.878885	-548.841587
30	0.01	0.02	5.40	4.84	-549.001582	-548.877932	-548.840940
31	0.01	0.11	5.51	3.83	-549.001412	-548.879554	-548.841454
32	0.01	0.05	5.52	4.29	-549.001395	-548.878823	-548.841263
33	0.01	0.04	5.62	4.44	-549.001228	-548.878573	-548.841130
34	0.00	0.03	5.83	4.56	-549.000898	-548.878392	-548.840844
35	0.00	0.01	5.84	5.24	-549.000878	-548.877297	-548.840447
36	0.00	0.02	5.90	4.91	-549.000795	-548.877835	-548.840606
37	0.00	0.02	5.91	4.98	-549.000768	-548.877711	-548.840383
38	0.00	0.03	6.15	4.56	-549.000383	-548.878388	-548.840259
39	0.00	0.02	6.21	4.91	-549.000300	-548.877826	-548.840252
40	0.00	0.02	6.21	4.92	-549.000287	-548.877810	-548.840178
41	0.00	0.02	6.22	4.93	-549.000284	-548.877791	-548.840349
42	0.00	0.02	6.22	4.87	-549.000273	-548.877893	-548.840314
43	0.00	0.01	6.23	5.17	-549.000266	-548.877408	-548.840232
44	0.00	0.02	6.32	4.91	-549.000121	-548.877835	-548.839999
45	0.00	0.00	6.45	5.71	-548.999905	-548.876557	-548.839660
46	0.00	0.01	6.62	5.02	-548.999638	-548.877654	-548.839852
47	0.00	0.04	6.66	4.46	-548.999570	-548.878545	-548.839784
48	0.00	0.00	6.75	5.84	-548.999433	-548.876344	-548.839218
49	0.00	0.00	6.76	6.05	-548.999411	-548.876006	-548.839103

50	0.00	0.01	6.89	5.04	-548.999202	-548.877617	-548.839395
51	0.00	0.00	6.90	7.07	-548.999196	-548.874382	-548.838397
52	0.00	0.00	6.94	6.44	-548.999133	-548.875396	-548.838700
53	0.00	0.00	7.07	6.09	-548.998922	-548.875943	-548.838440
54	0.00	0.00	7.15	6.17	-548.998794	-548.875827	-548.838596
55	0.00	0.01	7.52	5.31	-548.998210	-548.877191	-548.838352
56	0.00	0.00	7.65	6.59	-548.997999	-548.875149	-548.837957
57	0.00	0.00	7.75	6.20	-548.997834	-548.875767	-548.837697
58	0.00	0.00	7.82	6.31	-548.997720	-548.875591	-548.837789
59	0.00	0.00	7.95	6.52	-548.997527	-548.875269	-548.837510
60	0.00	0.00	8.12	7.13	-548.997250	-548.874292	-548.837255
61	0.00	0.00	8.63	7.38	-548.996434	-548.873898	-548.836438
62	0.00	0.00	8.70	7.66	-548.996333	-548.873449	-548.836311
63	0.00	0.00	8.82	7.86	-548.996128	-548.873133	-548.836008
64	0.00	0.00	9.09	8.29	-548.995700	-548.872443	-548.835200
65	0.00	0.00	9.37	8.75	-548.995260	-548.871707	-548.834735

Table S8. Different NICS aromaticity indices of rings in Phe, Tyr, Trp, and His aromatic amino acids and their three the most populated conformers calculated at the B3LYP/D3/aug-cc-pVTZ level. min, max, and *s* denote the minimum and maximum value of the population and the standard deviation. Population of the three most frequent conformers were taken according to total energies, T = 298.15 K, or Gibbs free energies, T=298.15 K.

**Parameter**	**conformer**	**Phe**	**Tyr**	**Trp**	**Trp**	**His**
		phenyl	phenyl	phenyl	pyrrole	imidazole
$NICS_{ZZ}(0)$ (ppm)	1	-14.6	-12.9	-15.20	-8.8	-13.7
	2	-15.2	-12.9	-15.26	-7.8	-11.3
	3	-14.8	-13.0	-15.40	-7.3	-12.1
	min	-15.3	-13.3	-15.5	-8.8	-13.8
	max	-14.5	-12.2	-15.0	-6.8	-11.3
	s	0.2	0.3	0.1	0.4	0.6
$NICS_{ZZ}(min_{rev})$ (ppm)	1	-28.9	-26.6	-29.9	-29.4*	-32.1*
	2	-29.4	-26.6	-29.9	-28.8*	-30.1*
	3	-29.0	-26.7	-30.1	-28.1	-30.8*
	min	-29.6	-26.9	-30.1	-28.5	-32.3
	max	-28.8	-25.9	-29.7	-26.8	-30.1
	s	0.2	0.2	0.1	0.5	0.6
$NICS_{ZZ}(min_{obv})$ (ppm)	1	-28.1	-25.48	-29.4	-28.5**	-31.2**
	2	-28.4	-25.41	-29.3	-27.6**	-30.0**
	3	-28.2	-25.47	-29.4	-27.0	-30.3**
	min	-28.6	-25.8	-29.9	-28.5	-31.4
	max	-27.9	-24.9	-29.1	-26.8	-30.0
	s	0.2	0.2	0.2	0.4	0.4
$NICS_{ZZ}(0)_{pav}$ (ppm)	*based on total energies*	-14.8	-12.8	-15.2	-8.3	-13.3
$NICS_{ZZ}(min_{rev})_{pav}$ (ppm)		-29.1	-26.5	-30.0	-29.1	-31.8
$NICS_{ZZ}(min_{obv})_{pav}$ (ppm)		-28.2	-25.4	-29.4	-28.1	-31.0
$NICS_{ZZ}(0)_{pav}$ (ppm)	*based on Gibbs free entalpies*	-14.9	-12.7	-15.3	-8.0	-13.2
$NICS_{ZZ}(min_{rev})_{pav}$ (ppm)		-29.2	-26.4	-30.0	-28.8	-31.7
$NICS_{ZZ}(min_{obv})_{pav}$ (ppm)		-28.3	-25.4	-29.4	-27.9	-30.9

* minimum at -0.9 Å, ** minimum at 0.9 Å

Table S9. The $NICS_{zz}$ values (ppm) for Phe and Tyr conformers calculated at the B3LYP/D3/aug-cc-pVTZ level

conformer	name	(0)	(rev)	(obv)	name	(0)	(rev)	(obv)
1	phe-23	-14.58	-28.93	-28.05	36-Tyr	-12.91	-26.58	-25.48
2	phe-21	-15.15	-29.36	-28.36	40-Tyr	-12.85	-26.57	-25.41
3	phe-18	-14.75	-29.02	-28.20	43-Tyr	-12.99	-26.74	-25.47
4	phe-8	-14.74	-28.96	-28.11	10-Tyr	-13.00	-26.71	-25.49
5	phe-4	-15.25	-29.50	-28.34	5-Tyr	-12.55	-26.30	-25.37
6	phe-7	-14.90	-29.08	-28.09	32-Tyr	-12.45	-26.21	-25.34
7	phe-11	-15.10	-29.24	-28.62	33-Tyr	-12.24	-26.06	-25.17
8	phe-16	-14.80	-29.03	-28.22	1-Tyr	-12.81	-26.46	-25.75
9	phe-13	-14.85	-29.09	-28.28	3-Tyr	-12.84	-26.48	-25.77
10	phe-1	-15.31	-29.55	-28.38	16-Tyr	-12.61	-26.26	-25.24
11	phe-9	-14.78	-28.99	-28.17	19-Tyr	-12.44	-26.14	-25.25
12	phe-12	-14.86	-29.06	-28.41	20-Tyr	-12.60	-26.25	-25.23
13	phe-14	-15.07	-29.22	-28.54	11-Tyr	-13.08	-26.79	-25.52
14	phe-10	-14.91	-29.08	-28.16	28-Tyr	-13.07	-26.80	-25.50
15	phe-26	-15.09	-29.18	-28.45	12-Tyr	-12.55	-26.23	-25.39
16	phe-24	-14.46	-27.86	-28.75	29-Tyr	-12.57	-26.27	-25.39
17	phe-17	-14.76	-28.99	-28.24	22-Tyr	-12.65	-26.30	-25.57
18	phe-15	-14.92	-29.09	-28.42	21-Tyr	-12.62	-26.29	-25.52
19	phe-19	-14.80	-29.05	-28.24	17-Tyr	-12.55	-26.21	-25.35
20	phe-20	-14.69	-28.64	-28.77	18-Tyr	-12.65	-26.36	-25.48
21	phe-25	-14.89	-29.34	-27.95	24-Tyr	-12.83	-26.46	-25.73
22					23-Tyr	-12.48	-26.16	-25.31
23					27-Tyr	-12.61	-26.25	-25.30
24					48-Tyr	-13.26	-26.93	-25.61
25					14-Tyr	-12.72	-26.35	-25.47
26					49-Tyr	-12.70	-26.34	-25.56
27					44-Tyr	-12.73	-26.35	-25.61
28					45-Tyr	-12.74	-26.37	-25.49
29					25-Tyr	-12.17	-25.94	-25.01
30					26-Tyr	-12.50	-26.20	-25.36
31					30-Tyr	-12.15	-25.97	-24.99
32					31-Tyr	-12.52	-26.23	-25.37
33					38-Tyr	-12.33	-25.91	-25.64
34					39-Tyr	-12.28	-25.90	-25.50
35					34-Tyr	-12.53	-25.34	-26.27
36					35-Tyr	-12.56	-25.38	-26.28
37					46-Tyr	-12.46	-26.43	-24.94
38					47-Tyr	-12.48	-26.47	-24.93
average		-14.81	-29.09	-28.17		-12.82	-26.52	-25.43

Table S10. The $NICS_{ZZ}$ values (ppm) for **Trp** pyrrole and phenyl rings in different conformers calculated at the B3LYP/D3/aug-cc-pVTZ level

**conformer**	**name**	**(0)**	**(rev)**	**(obv)**	**(0)**	**(rev)**	**(obv)**
			pyrrole			phenyl	
1	30-Trp	-8.76	-29.41	-28.53	-15.20	-29.96	-29.37
2	37-Trp	-7.84	-28.82	-27.60	-15.26	-29.96	-29.33
3	41-Trp	-7.29	-28.13	-26.98	-15.40	-30.13	-29.41
4	35-Trp	-8.00	-28.98	-27.72	-15.13	-29.98	-29.19
5	32-Trp	-7.19	-27.83	-27.21	-15.35	-30.04	-29.37
6	6-Trp	-8.06	-28.89	-27.74	-15.33	-30.12	-29.37
7	4-Trp	-8.08	-28.98	-27.70	-15.28	-29.94	-29.40
8	29-Trp	-8.05	-28.93	-27.89	-15.12	-30.02	-29.09
9	2-Trp	-7.86	-28.62	-27.56	-15.33	-30.08	-29.39
10	1-Trp	-8.25	-29.06	-27.85	-15.27	-29.93	-29.41
11	11-Trp	-6.95	-27.69	-26.96	-15.25	-29.89	-29.39
12	13-Trp	-7.65	-28.21	-27.94	-15.41	-30.01	-29.53
13	16-Trp	-7.85	-28.56	-27.72	-15.37	-30.00	-29.46
14	26-Trp	-7.38	-27.99	-27.42	-15.30	-29.94	-29.43
15	10-Trp	-7.84	-28.82	-27.60	-14.99	-29.78	-29.11
16	7-Trp	-7.32	-27.94	-27.25	-15.27	-29.84	-29.47
17	20-Trp	-7.46	-28.07	-27.45	-15.32	-29.99	-29.40
18	25-Trp	-7.49	-28.45	-27.19	-15.14	-29.81	-29.29
19	12-Trp	-6.99	-27.71	-27.00	-15.26	-29.90	-29.41
20	23-Trp	-7.78	-28.46	-27.65	-15.37	-29.99	-29.48
21	18-Trp	-7.40	-28.04	-27.65	-15.39	-29.96	-29.56
22	21-Trp	-7.73	-28.29	-27.98	-15.40	-29.98	-29.51
23	19-Trp	-7.85	-28.75	-27.55	-15.20	-29.91	-29.33
24	38-Trp	-7.59	-28.16	-27.88	-15.27	-29.94	-29.56
25	17-Trp	-8.28	-28.93	-28.24	-15.26	-29.97	-29.41
26	9-Trp	-7.87	-28.82	-27.68	-15.01	-29.79	-29.10
27	43-Trp	-6.78	-27.44	-27.40	-15.49	-29.88	-29.87
28	33-Trp	-7.00	-27.49	-27.24	-15.35	-30.02	-29.39
29	27-Trp	-7.68	-28.29	-27.78	-15.30	-29.91	-29.54
30	8-Trp	-7.70	-28.24	-27.92	-15.30	-29.81	-29.81
31	24-Trp	-7.55	-28.17	-27.77	-15.42	-29.97	-29.57
32	22-Trp	-6.85	-27.53	-27.35	-15.47	-29.88	-29.83
33	36-Trp	-7.39	-27.37	-27.94	-15.31	-29.90	-29.39
34	42-Trp	-7.27	-28.27	-26.83	-15.17	-29.97	-29.14
35	34-Trp	-7.82	-28.35	-28.13	-15.21	-29.84	-29.60
36	28-Trp	-7.75	-28.64	-27.42	-15.10	-29.69	-29.38
37	39-Trp	-8.17	-28.90	-28.09	-15.17	-29.87	-29.40
average		-8.32	-29.05	-28.09	-15.24	-29.98	-29.36

Table S11. The $NICS_{ZZ}$ values (ppm) for His ($N^{\varepsilon}H$) and His ($N^{\delta}H$) conformers calculated at the B3LYP/D3/aug-cc-pVTZ level

**conformer**	**name**	**(0)**	**(rev)** ($N^{\varepsilon}H$)	**(obv)**	**name**	**(0)**	**(rev)** ($N^{\delta}H$)	**(obv)**
1	65-His	-13.66	-32.05	-31.16	32-His-tau2	-12.02	-30.82	-30.02
2	85-His	-11.27	-30.13	-30.04	16-His-tau2	-11.70	-30.69	-29.89
3	60-His	-12.14	-30.84	-30.27	4-His-tau2	-12.72	-31.50	-30.10
4	88-His	-13.51	-31.96	-30.96	1-His-tau2	-12.03	-30.57	-30.07
5	13-His	-11.84	-30.47	-30.20	3-His-tau2	-11.95	-30.97	-29.65
6	12-His	-13.22	-31.77	-30.77	24-His-tau2	-11.70	-30.73	-29.84
7	10-His	-12.88	-31.07	-31.44	2-His-tau2	-12.71	-30.49	-31.01
8	18-His	-12.24	-30.85	-30.61	19-His-tau2	-12.98	-31.55	-31.02
9	40-His	-12.09	-30.81	-30.31	5-His-tau2	-11.12	-30.23	-29.26
10	55-His	-12.14	-30.88	-30.28	14-His-tau2	-11.83	-30.62	-30.33
11	26-His	-13.62	-31.96	-31.15	15-His-tau2	-11.49	-30.58	-29.58
12	25-His	-12.84	-31.06	-30.87	8-His-tau2	-10.84	-29.75	-29.54
13	86-His	-12.98	-31.55	-31.02	30-His-tau2	-11.27	-30.04	-29.53
14	29-His	-12.79	-31.41	-30.31	6-His-tau2	-11.19	-30.31	-29.34
15	82-His	-12.11	-30.83	-30.36	22-His-tau2	-11.44	-30.55	-29.48
16	2-His	-12.61	-31.21	-30.46	17-His-tau2	-11.55	-30.39	-29.94
17	51-His	-12.65	-31.30	-30.76	9-His-tau2	-10.94	-30.02	-29.19
18	48-His	-12.21	-30.54	-30.52	33-His-tau2	-12.13	-30.46	-30.31
19	47-His	-13.07	-31.73	-30.65	34-His-tau2	-11.73	-30.45	-29.88
20	24-His	-13.78	-32.27	-31.28	13-His-tau2	-10.80	-29.73	-29.58
21	96-His	-12.78	-31.44	-30.58	29-His-tau2	-11.68	-30.41	-29.67
22	21-His	-12.83	-31.01	-30.92	12-His-tau2	-10.89	-30.01	-29.21
23	46-His	-12.76	-31.34	-30.64	7-His-tau2	-12.89	-31.85	-30.25
24	64-His	-12.10	-30.64	-30.52	23-His-tau2	-10.93	-29.84	-29.66
25	53-His	-12.75	-31.36	-30.88	21-His-tau2	-11.75	-30.34	-30.11
26	36-His	-11.98	-30.60	-30.47	26-His-tau2	-11.54	-30.69	-29.62
27	54-His	-11.80	-30.46	-30.12	18-His-tau2	-10.85	-29.71	-29.57
28	58-His	-12.20	-30.81	-30.54	31-His-tau2	-11.28	-30.45	-29.68
29	49-His	-12.98	-31.72	-30.33	35-His-tau2	-10.55	-29.51	-29.35
30	80-His	-13.11	-31.74	-30.70				
31	56-His	-12.20	-30.72	-30.54				
32	39-His	-12.12	-30.66	-30.48				
33	35-His	-12.12	-30.54	-30.51				
34	99-His	-11.86	-30.66	-30.15				
35	92-His	-13.61	-32.04	-31.22				
36	95-His	-12.53	-31.22	-30.10				
average		-11.87	-28.30	-27.60		-12.04	-30.85	-29.99

Table S12. The $NICS_{zz}$ values (ppm) for both ($N^{\epsilon}H$) and His ($N^{\delta}H$) conformers calculated at the B3LYP/D3/aug-cc-pVTZ level

conformer	name	(0)	(rev)	(obv)	conformer	name	(0)	(rev)	(obv)
1	65-His	-13.66	-32.05	-31.16	36	53-His	-11.98	-30.60	-30.47
2	85-His	-11.27	-30.13	-30.04	37	8-His-tau2	-10.84	-29.75	-29.54
3	60-His	-12.14	-30.84	-30.27	38	36-His	-12.83	-31.01	-30.92
4	88-His	-11.84	-30.47	-30.20	39	30-His-tau2	-11.27	-30.04	-29.53
5	32-His-tau2	-12.02	-30.82	-30.02	40	6-His-tau2	-11.19	-30.31	-29.34
6	13-His	-13.51	-31.96	-30.96	41	22-His-tau2	-11.44	-30.55	-29.48
7	16-His-tau2	-11.70	-30.69	-29.89	42	54-His	-12.75	-31.36	-30.88
8	4-His-tau2	-12.72	-31.50	-30.10	43	58-His	-13.11	-31.74	-30.70
9	12-His	-13.22	-31.77	-30.77	44	17-His-tau2	-11.55	-30.39	-29.94
10	1-His-tau2	-12.03	-30.57	-30.07	45	49-His	-12.20	-30.72	-30.54
11	3-His-tau2	-11.95	-30.97	-29.65	46	80-His	-12.20	-30.81	-30.54
12	10-His	-13.62	-31.96	-31.15	47	9-His-tau2	-10.94	-30.02	-29.19
13	18-His	-12.14	-30.88	-30.28	48	56-His	-12.12	-30.66	-30.48
14	40-His	-12.88	-31.44	-31.44	49	39-His	-12.12	-30.54	-30.51
15	55-His	-12.24	-30.85	-30.61	50	35-His	-12.98	-31.72	-30.33
16	26-His	-12.09	-30.81	-30.31	51	33-His-tau2	-12.13	-30.46	-30.31
17	25-His	-12.21	-30.54	-30.52	52	34-His-tau2	-11.73	-30.45	-29.88
18	86-His	-12.61	-31.21	-30.46	53	13-His-tau2	-10.80	-29.73	-29.58
19	29-His	-12.84	-31.06	-30.87	54	29-His-tau2	-11.68	-30.41	-29.67
20	24-His-tau2	-11.70	-30.73	-29.84	55	12-His-tau2	-10.89	-30.01	-29.21
21	82-His	-11.80	-30.46	-30.12	56	7-His-tau2	-12.89	-31.85	-30.25
22	2-His-tau2	-12.71	-31.01	-31.01	57	23-His-tau2	-10.93	-29.84	-29.66
23	2-His	-12.79	-31.41	-30.31	58	21-His-tau2	-11.75	-30.34	-30.11
24	19-His-tau2	-12.98	-31.55	-31.02	59	99-His	-11.86	-30.66	-30.15
25	51-His	-12.98	-31.55	-31.02	60	92-His	-13.61	-32.04	-31.22
26	48-His	-12.65	-31.30	-30.76	61	26-His-tau2	-11.54	-30.69	-29.62
27	47-His	-13.07	-31.73	-30.65	62	95-His	-12.53	-31.22	-30.10
28	24-His	-13.78	-32.27	-31.28	63	18-His-tau2	-10.85	-29.71	-29.57
29	5-His-tau2	-11.12	-30.23	-29.26	64	31-His-tau2	-11.28	-30.45	-29.68
30	96-His	-12.10	-30.64	-30.52	65	35-His-tau2	-10.55	-29.51	-29.35
31	21-His	-12.11	-30.83	-30.36					
32	14-His-tau2	-11.83	-30.62	-30.33					
33	46-His	-12.78	-31.44	-30.58					
34	64-His	-12.76	-31.34	-30.64					
35	15-His-tau2	-11.49	-30.58	-29.58					
average							-13.28	-31.74	-30.97

Table S13. Different integral INICS aromaticity indices of rings in Phe, Tyr, Trp, and His aromatic amino acids and their three the most populated conformers calculated at the B3LYP/D3/aug-cc-pVTZ level. Population of the three most frequent conformers were taken according to total energies, T = 298.15 K, or Gibbs free energies, T=298.15 K.The integration range was from -9.8 to 0.0 Å, and from 0.0 to 9.8 Å for $INICS_{zz}$(rev) and $INICS_{zz}$(obv), respectively, while $INICS_{zz}$(tot) = $INICS_{zz}$(obv) + $INICS_{zz}$(rev).

Parameter	conformer	Phe	Tyr	Trp	Trp	His
		phenyl	phenyl	phenyl	pyrrole	imidazole
**INICS$_{zz}$**(tot) (ppm·Å)	1	-144.2	-129.6	-155.3	-140.1	-134.6
	2	-145.1	-129.4	-155.3	-134.1	-130.2
	3	-144.5	-128.3	-155.3	-134.9	-132.3
	min	-145.4	-130.3	-156.5	-140.1	-135.4
	max	-143.5	-127.5	-154.7	-134.1	-130.2
	s	0.5	0.1	0.5	1.5	1.3
**INICS$_{zz}$(rev)** (ppm·Å)	1	-73.8	-67.2	-79.1	-72.6	-70.0
	2	-74.8	-67.1	-78.7	-68.9	-65.5
	3	-73.8	-65.9	-79.4	-70.1	-67.0
	min	-75.3	-68.1	-79.5	-72.6	-70.5
	max	-73.2	-65.4	-78.3	-68.0	-65.5
	s	0.5	0.0	0.3	1.1	1.0
**INICS$_{zz}$(obv)** (ppm·Å)	1	-70.4	-62.4	-76.2	-67.6	-64.6
	2	-70.3	-62.3	-76.5	-65.2	-64.6
	3	-70.7	-62.4	-75.9	-64.8	-65.3
	min	-70.9	-63.1	-78.4	-68.8	-66.7
	max	-69.7	-61.5	-75.3	-64.8	-63.6
	s	0.4	0.2	0.7	0.9	0.8
**INICS$_{zz}$**(tot)$_{pav}$ (ppm·Å)	*based on total energies*	-145.0	-128.7	-155.1	-138.3	-133.8
**INICS$_{zz}$(rev)**$_{pav}$ (ppm·Å)		-74.7	-66.9	-79.0	-71.6	-69.0
**INICS$_{zz}$(obv)**$_{pav}$ (ppm·Å)		-70.3	-62.4	-76.1	-66.8	-64.8
**INICS$_{zz}$**(tot)$_{pav}$ (ppm·Å)	*based on Gibbs free energies*	-144.8	-129.0	-155.2	-137.5	-133.6
**INICS$_{zz}$(rev)**$_{pav}$ (ppm·Å)		-74.4	-66.8	-79.0	-71.0	-68.7
**INICS$_{zz}$(obv)**$_{pav}$ (ppm·Å)		-70.4	-62.5	-76.2	-66.5	-65.0

* minimum at -0.9 Å, ** minimum at 0.9 Å

Table S14. The integral $NICS_{zz}$ values (ppm·Å) for Phe and Tyr conformers calculated at the B3LYP/D3/aug-cc-pVTZ level

conformer	name	Int(rev)	Int(obv)	Int(tot)	name	Int(rev)	Int(obv)	Int(tot)
1	phe-23	-73.76	-70.42	-144.18	36-Tyr	-67.16	-62.43	-129.01
2	phe-21	-74.77	-70.34	-145.11	40-Tyr	-67.07	-62.32	-127.46
3	phe-18	-73.83	-70.65	-144.48	43-Tyr	-65.90	-62.43	-128.33
4	phe-8	-73.92	-70.57	-144.49	10-Tyr	-67.53	-62.21	-129.74
5	phe-4	-75.20	-70.19	-145.39	5-Tyr	-67.52	-62.22	-129.75
6	phe-7	-74.17	-70.87	-145.04	32-Tyr	-66.28	-62.86	-128.92
7	phe-11	-74.56	-69.95	-144.51	33-Tyr	-66.09	-62.71	-129.14
8	phe-16	-73.95	-70.81	-144.77	1-Tyr	-67.70	-62.15	-129.85
9	phe-13	-73.97	-70.85	-144.82	3-Tyr	-67.72	-62.07	-129.79
10	phe-1	-75.34	-70.02	-145.36	16-Tyr	-66.14	-62.63	-128.78
11	phe-9	-73.97	-70.70	-144.67	19-Tyr	-66.92	-62.13	-129.04
12	phe-12	-74.03	-70.38	-144.41	20-Tyr	-66.98	-62.14	-129.12
13	phe-14	-74.59	-69.78	-144.37	11-Tyr	-66.43	-63.00	-129.43
14	phe-10	-74.13	-70.92	-145.05	28-Tyr	-66.26	-62.93	-129.19
15	phe-26	-74.47	-70.09	-144.56	12-Tyr	-66.42	-62.99	-129.41
16	phe-24	-73.20	-70.34	-143.54	29-Tyr	-66.32	-62.97	-129.29
17	phe-17	-73.69	-70.67	-144.36	22-Tyr	-66.43	-62.53	-128.96
18	phe-15	-74.21	-70.04	-144.24	21-Tyr	-66.39	-62.48	-128.88
19	phe-19	-73.87	-70.65	-144.52	17-Tyr	-66.30	-62.88	-129.19
20	phe-20	-73.44	-70.06	-143.51	18-Tyr	-66.18	-62.78	-128.97
21	phe-25	-74.75	-69.67	-144.41	24-Tyr	-66.99	-61.92	-128.91
22					23-Tyr	-66.45	-63.11	-129.55
23					27-Tyr	-68.14	-62.18	-130.32
24					48-Tyr	-66.68	-62.05	-128.74
25					14-Tyr	-66.37	-63.09	-129.46
26					49-Tyr	-66.69	-62.02	-128.71
27					44-Tyr	-65.42	-62.39	-127.81
28					45-Tyr	-65.42	-62.38	-127.81
29					25-Tyr	-66.58	-62.12	-128.70
30					26-Tyr	-66.61	-62.14	-128.75
31					30-Tyr	-66.04	-62.81	-129.39
32					31-Tyr	-66.08	-62.84	-128.85
33					38-Tyr	-65.58	-62.01	-129.59
34					39-Tyr	-65.48	-61.98	-127.59
35					34-Tyr	-66.22	-62.72	-128.80
36					35-Tyr	-66.26	-62.75	-128.93
37					46-Tyr	-66.69	-61.45	-128.13
38					47-Tyr	-66.73	-61.50	-128.23
average		**-74.15**	**-70.39**	**-144.54**		**-67.18**	**-62.41**	**-129.59**

Table S15. The integral $NICS_{ZZ}$ values (ppm·Å) for **Trp** phenyl and pyrrole rings in various conformers calculated at the B3LYP/D3/aug-cc-pVTZ level

conformer	name	Int(rev)	Int(obv)	Int(tot)	Int(rev)	Int(obv)	Int(tot)
			Phenyl			pyrrole	
1	30-Trp	-79.14	-76.19	-155.32	-72.55	-67.57	-140.11
2	37-Trp	-78.74	-76.53	-155.28	-68.88	-65.19	-134.06
3	41-Trp	-79.38	-75.87	-155.26	-70.13	-64.75	-134.88
4	35-Trp	-79.05	-76.16	-155.21	-71.56	-67.20	-138.76
5	32-Trp	-78.89	-76.63	-155.52	-69.21	-65.85	-135.06
6	6-Trp	-79.49	-75.59	-155.08	-71.72	-65.81	-137.53
7	4-Trp	-79.14	-76.09	-155.23	-71.79	-66.00	-137.79
8	29-Trp	-79.04	-75.83	-154.87	-71.36	-67.35	-138.71
9	2-Trp	-78.87	-76.60	-155.47	-69.69	-66.30	-136.00
10	1-Trp	-79.11	-76.11	-155.23	-71.96	-66.11	-138.07
11	11-Trp	-78.71	-76.66	-155.36	-69.06	-65.55	-134.61
12	13-Trp	-79.11	-76.26	-155.38	-70.26	-65.89	-136.15
13	16-Trp	-79.33	-75.27	-154.60	-70.88	-65.55	-136.43
14	26-Trp	-78.77	-76.84	-155.61	-69.57	-66.29	-135.86
15	10-Trp	-78.63	-75.87	-154.50	-71.22	-66.94	-138.15
16	7-Trp	-78.73	-76.96	-155.69	-69.57	-66.35	-135.92
17	20-Trp	-79.46	-75.26	-154.71	-71.24	-65.14	-136.38
18	25-Trp	-78.61	-76.36	-154.97	-70.39	-66.47	-136.86
19	12-Trp	-78.73	-76.66	-155.39	-69.10	-65.63	-134.73
20	23-Trp	-79.32	-75.25	-154.57	-70.72	-65.34	-136.06
21	18-Trp	-78.94	-76.57	-155.51	-69.77	-66.02	-135.79
22	21-Trp	-79.09	-76.14	-155.23	-70.46	-65.82	-136.28
23	19-Trp	-78.82	-76.47	-155.29	-70.95	-67.09	-138.03
24	38-Trp	-78.81	-77.18	-155.99	-69.86	-67.61	-137.47
25	17-Trp	-79.13	-75.77	-154.90	-71.49	-66.97	-138.46
26	9-Trp	-78.60	-75.89	-154.50	-71.18	-67.12	-138.29
27	43-Trp	-78.37	-78.37	-156.73	-67.98	-67.98	-135.96
28	33-Trp	-78.86	-76.69	-155.56	-68.63	-65.71	-134.35
29	27-Trp	-78.79	-76.96	-155.75	-70.09	-67.13	-137.22
30	8-Trp	-78.27	-78.04	-156.30	-69.57	-68.82	-138.39
31	24-Trp	-79.04	-76.40	-155.44	-70.10	-65.83	-135.93
32	22-Trp	-78.47	-78.07	-156.54	-68.33	-67.50	-135.84
33	36-Trp	-78.72	-76.81	-155.53	-69.43	-66.32	-135.75
34	42-Trp	-78.92	-75.92	-154.84	-70.25	-64.90	-135.16
35	34-Trp	-78.61	-76.38	-154.99	-70.15	-67.25	-137.40
36	28-Trp	-78.33	-76.27	-154.59	-70.63	-66.47	-137.10
37	39-Trp	-78.85	-75.89	-154.74	-71.35	-66.99	-138.34
average	**Trp**	**-79.10**	**-76.19**	**-155.29**	**-71.56**	**-66.77**	**-138.34**

Table S16. The integral $NICS_{ZZ}$ values (ppm·Å) for His conformers calculated at the B3LYP/D3/aug-cc-pVTZ level

**conformer**	**name**	**Int(rev)**	**Int(obv)** ($N^{\varepsilon}H$)	**Int(tot)**	**name**	**Int(rev)**	**Int(obv)** ($N^{\delta}H$)	**Int(tot)**
1	65-His	-70.00	-64.58	-134.58	32-His-tau2	-63.43	-67.10	-130.52
2	85-His	-65.54	-64.64	-130.18	16-His-tau2	-63.64	-66.54	-130.18
3	60-His	-67.01	-65.30	-132.31	4-His-tau2	-68.66	-62.80	-131.46
4	88-His	-66.48	-63.97	-130.44	1-His-tau2	-66.34	-64.73	-131.06
5	13-His	-69.79	-64.52	-134.30	3-His-tau2	-62.40	-67.45	-129.85
6	12-His	-69.47	-63.81	-133.28	24-His-tau2	-63.06	-66.70	-129.77
7	10-His	-69.82	-64.64	-134.46	2-His-tau2	-67.64	-63.73	-131.37
8	18-His	-67.37	-63.88	-131.25	19-His-tau2	-64.13	-68.86	-132.98
9	40-His	-68.61	-64.35	-132.97	5-His-tau2	-61.93	-65.90	-127.83
10	55-His	-67.10	-64.39	-131.50	14-His-tau2	-62.75	-66.90	-129.64
11	26-His	-67.19	-64.16	-131.35	15-His-tau2	-63.09	-66.37	-129.46
12	25-His	-66.27	-65.71	-131.98	8-His-tau2	-63.67	-64.20	-127.87
13	86-His	-67.83	-64.84	-132.67	30-His-tau2	-63.32	-65.68	-129.00
14	29-His	-67.53	-66.37	-133.90	6-His-tau2	-62.15	-66.06	-128.21
15	82-His	-66.48	-64.12	-130.60	22-His-tau2	-62.48	-66.38	-128.86
16	2-His	-68.27	-65.09	-133.36	17-His-tau2	-62.26	-66.26	-128.52
17	51-His	-68.86	-64.13	-132.98	9-His-tau2	-61.94	-65.46	-127.40
18	48-His	-68.17	-64.26	-132.44	33-His-tau2	-66.40	-63.64	-130.04
19	47-His	-69.26	-63.74	-133.00	34-His-tau2	-66.29	-64.14	-130.43
20	24-His	-70.49	-64.95	-135.44	13-His-tau2	-63.50	-64.49	-127.99
21	96-His	-66.12	-66.24	-132.36	29-His-tau2	-62.66	-66.60	-129.27
22	21-His	-67.23	-64.00	-131.23	12-His-tau2	-65.39	-61.98	-127.37
23	46-His	-68.49	-64.34	-132.83	7-His-tau2	-62.48	-69.44	-131.92
24	64-His	-68.16	-64.84	-133.00	23-His-tau2	-63.53	-64.52	-128.05
25	53-His	-66.39	-65.73	-132.11	21-His-tau2	-62.21	-66.28	-128.48
26	36-His	-67.14	-66.68	-133.82	26-His-tau2	-62.88	-66.50	-129.38
27	54-His	-68.42	-64.02	-132.44	18-His-tau2	-63.46	-64.52	-127.97
28	58-His	-69.32	-63.63	-132.95	31-His-tau2	-65.88	-63.37	-129.25
29	49-His	-66.60	-65.87	-132.47	35-His-tau2	-63.93	-63.47	-127.41
30	80-His	-66.98	-64.34	-131.32				
31	56-His	-66.46	-65.90	-132.36				
32	39-His	-66.53	-65.51	-132.04				
33	35-His	-69.03	-64.39	-133.42				
34	99-His	-66.88	-64.13	-131.01				
35	92-His	-70.08	-64.52	-134.60				
36	95-His	-67.93	-64.59	-132.52				
average		**-69.33**	**-64.59**	**-133.92**		**-64.27**	**-66.29**	**-130.55**

Table S17. The $NICS_{ZZ}$ values (ppm) for both ($N^{\varepsilon}H$) and His ($N^{\delta}H$) conformers calculated at the B3LYP/D3/aug-cc-pVTZ level

conformer	name	Int(rev)	Int(obv)	Int(tot)	conformer	name	Int(rev)	Int(obv)	Int(tot)
1	65-His	-70.00	-64.58	-134.58	36	53-His	-65.73	-66.39	-132.11
2	85-His	-64.64	-65.54	-130.18	37	8-His-tau2	-63.67	-64.20	-127.87
3	60-His	-67.01	-65.30	-132.31	38	36-His	-67.14	-66.68	-133.82
4	88-His	-63.97	-66.48	-130.44	39	30-His-tau2	-63.32	-65.68	-129.00
5	32-His-tau2	-63.43	-67.10	-130.52	40	6-His-tau2	-62.15	-66.06	-128.21
6	13-His	-69.79	-64.52	-134.30	41	22-His-tau2	-62.48	-66.38	-128.86
7	16-His-tau2	-63.64	-66.54	-130.18	42	54-His	-64.02	-68.42	-132.44
8	4-His-tau2	-68.66	-62.80	-131.46	43	58-His	-63.63	-69.32	-132.95
9	12-His	-63.81	-69.47	-133.28	44	17-His-tau2	-62.26	-66.26	-128.52
10	1-His-tau2	-66.34	-64.73	-131.06	45	49-His	-65.87	-66.60	-132.47
11	3-His-tau2	-62.40	-67.45	-129.85	46	80-His	-64.34	-66.98	-131.32
12	10-His	-69.82	-64.64	-134.46	47	9-His-tau2	-61.94	-65.46	-127.40
13	18-His	-63.88	-67.37	-131.25	48	56-His	-65.90	-66.46	-132.36
14	40-His	-64.35	-68.61	-132.97	49	39-His	-65.51	-66.53	-132.04
15	55-His	-64.39	-67.10	-131.50	50	35-His	-64.39	-69.03	-133.42
16	26-His	-64.16	-67.19	-131.35	51	33-His-tau2	-66.40	-63.64	-130.04
17	25-His	-65.71	-66.27	-131.98	52	34-His-tau2	-66.29	-64.14	-130.43
18	86-His	-67.83	-64.84	-132.67	53	13-His-tau2	-63.50	-64.49	-127.99
19	29-His	-67.53	-66.37	-133.90	54	29-His-tau2	-62.66	-66.60	-129.27
20	24-His-tau2	-63.06	-66.70	-129.77	55	12-His-tau2	-65.39	-61.98	-127.37
21	82-His	-64.12	-66.48	-130.60	56	7-His-tau2	-62.48	-69.44	-131.92
22	2-His-tau2	-67.64	-63.73	-131.37	57	23-His-tau2	-63.53	-64.52	-128.05
23	2-His	-68.27	-65.09	-133.36	58	21-His-tau2	-62.21	-66.28	-128.48
24	19-His-tau2	-64.13	-68.86	-132.98	59	99-His	-66.88	-64.13	-131.01
25	51-His	-64.13	-68.86	-132.98	60	92-His	-70.08	-64.52	-134.60
26	48-His	-64.26	-68.17	-132.44	61	26-His-tau2	-62.88	-66.50	-129.38
27	47-His	-63.74	-69.26	-133.00	62	95-His	-67.93	-64.59	-132.52
28	24-His	-64.95	-70.49	-135.44	63	18-His-tau2	-63.46	-64.52	-127.97
29	5-His-tau2	-61.93	-65.90	-127.83	64	31-His-tau2	-65.88	-63.37	-129.25
30	96-His	-66.12	-66.24	-132.36	65	35-His-tau2	-63.93	-63.47	-127.41
31	21-His	-64.00	-67.23	-131.23					
32	14-His-tau2	-62.75	-66.90	-129.64					
33	46-His	-64.34	-68.49	-132.83					
34	64-His	-64.84	-68.16	-133.00					
35	15-His-tau2	-63.09	-66.37	-129.46					
average							-13.28	-31.74	-30.97

Table S18. Various $NICS_{ZZ}$ indices for a series of benzenes monosubstituted with groups of different σ- and π-electron activity.[64-67] The values are ordered according to decreasing $INICS_{ZZ}(tot)$ index. The differences are are taken with respect to the adequate values for the unsubstituted benzene.

Substituent	$INICS_{ZZ}(tot)$	$NICS_{ZZ}(min_{rev})$	$NICS_{ZZ}(0)$	$\Delta(INICS_{ZZ}(tot))$	$\Delta(NICS_{ZZ}(min_{rev}))$	$\Delta(NICS_{ZZ}(0))$
H	-147.25	-29.91	-16.12	0.00	0.00	0.00
CF_3	-144.75	-29.19	-15.01	2.50	0.72	1.11
$MeSO_2$	-144.49	-29.34	-15.32	2.77	0.57	0.80
MeSO	-143.22	-29.28	-14.34	4.03	0.62	1.78
SiH_3	-142.87	-28.87	-14.45	4.38	1.04	1.67
Li	-142.78	-28.93	-15.69	4.47	0.98	0.43
$B(OH)_2$	-142.60	-28.48	-13.59	4.66	1.43	2.53
Na	-142.47	-28.80	-16.54	4.78	1.10	-0.43
BF_2	-142.28	-28.44	-13.58	4.97	1.46	2.54
*t*Bu	-142.23	-28.30	-13.83	5.02	1.60	2.29
CH_3	-141.86	-28.76	-14.70	5.39	1.14	1.42
F	-141.77	-28.73	-16.49	5.48	1.18	-0.37
$CONH_2$	-141.64	-28.62	-14.03	5.61	1.28	2.09
BH_2	-141.44	-28.15	-13.58	5.81	1.75	2.54
COOH	-140.41	-28.06	-13.54	6.84	1.85	2.57
NO_2	-139.80	-27.97	-14.30	7.46	1.94	1.81
CN	-139.71	-28.24	-14.48	7.54	1.67	1.64
CFO	-139.56	-27.91	-13.49	7.69	1.99	2.63
$COCH_3$	-139.15	-27.75	-12.98	8.10	2.16	3.13
CHO	-138.89	-27.84	-12.78	8.36	2.07	3.34
Ph	-138.87	-27.54	-13.37	8.38	2.36	2.75
NC	-138.65	-28.05	-15.15	8.60	1.85	0.97
Cl	-138.31	-27.80	-14.95	8.94	2.10	1.17
Br	-137.83	-27.55	-14.77	9.42	2.35	1.35
OMe	-137.75	-27.59	-14.09	9.50	2.31	2.03
CCH	-136.93	-27.58	-13.74	10.32	2.32	2.38
OH	-134.36	-27.13	-14.03	12.90	2.78	2.09
NMe_2	-133.21	-26.70	-11.79	14.04	3.20	4.33
SMe	-133.03	-26.58	-11.97	14.22	3.32	4.15
NO	-132.32	-26.38	-11.53	14.93	3.53	4.59
SH	-130.70	-26.32	-12.59	16.55	3.59	3.53
NH_2	-125.10	-25.41	-11.02	22.15	4.50	5.10

Table S19. Tables with the optimized Cartesian coordinates Phe conformers calculated at the B3LYP/D3/aug-cc-pVTZ .

atom	1			2			3		
	x	y	z	x	y	z	x	y	z
N	-3.127126	2.428961	0.459405	2.632489	-2.492191	-1.551277	-3.135341	-2.485785	-0.305448
C	-3.431552	1.442627	-0.583555	3.387674	-1.475512	-0.809452	-3.371128	-1.472986	0.731534
C	-4.951020	1.336085	-0.780318	3.624293	-1.955740	0.638331	-4.860190	-1.465537	1.127978
O	-5.445877	0.803220	-1.736630	4.055964	-1.240113	1.503402	-5.361825	-0.590840	1.781361
H	-2.254268	2.216260	0.925876	1.638016	-2.295681	-1.532511	-3.231369	-2.085331	-1.231562
H	-3.010345	1.706494	-1.556355	4.390477	-1.427169	-1.240615	-2.846919	-1.805035	1.631213
C	-2.906827	0.044403	-0.193144	2.792712	-0.050102	-0.836786	-2.887105	-0.053511	0.385611
C	-1.403887	0.001207	-0.088260	1.348866	-0.001441	-0.401841	-1.396248	-0.001103	0.179524
H	-3.259940	-0.655890	-0.949395	2.884900	0.333983	-1.853825	-3.194430	0.611041	1.191931
H	-3.359423	-0.245443	0.757406	3.407248	0.573907	-0.189351	-3.408482	0.281646	-0.514561
C	1.394254	0.001354	0.089036	-1.339526	-0.001462	0.397960	1.386157	-0.001339	-0.179554
C	-0.615381	-0.000196	-1.241340	0.320366	0.000798	-1.346265	-0.533075	0.000642	1.277702
C	-0.767211	-0.000949	1.152796	1.008250	0.000650	0.954032	-0.840636	0.000355	-1.099429
C	0.622047	-0.000334	1.243307	-0.323563	0.000800	1.348815	0.539045	0.000868	-1.280782
C	0.770178	-0.001082	-1.155539	-1.014393	0.000654	-0.952700	0.844758	0.000577	1.102539
H	-1.094023	-0.007141	-2.212847	0.566383	0.018220	-2.401229	-0.946616	0.010235	2.278906
H	-1.363000	-0.016090	2.057333	1.793853	-0.001036	1.697070	-1.492972	0.014289	-1.963974
H	1.097543	-0.006037	2.214906	-0.568704	0.004820	2.402391	0.950113	0.007448	-2.281430
H	1.364959	-0.008487	-2.059071	-1.796362	0.007734	-1.700259	1.496778	0.008251	1.965724
H	2.473609	-0.001430	0.155938	-2.375585	0.000752	0.708294	2.458783	0.002111	-0.317327
O	-5.673408	1.848513	0.226670	3.346268	-3.248288	0.836364	-5.540293	-2.542495	0.711235
H	-5.019729	2.234757	0.848381	3.001927	-3.573251	-0.024787	-4.897600	-3.075602	0.195801
H	-3.043792	3.361331	0.075000	2.921241	-2.551566	-2.517207	-2.199981	-2.863857	-0.244806
atom	4			5			6		
	x	y	z	x	y	z	x	y	z
N	-3.075180	2.479850	0.661115	-2.691362	2.194108	-1.982712	-3.269700	-2.102392	-1.188203
C	-3.424298	1.507919	-0.357616	-3.373180	1.476499	-0.923014	-3.473858	-1.477916	0.105513
C	-4.933249	1.435154	-0.470176	-3.364185	2.217399	0.415957	-4.941622	-1.414840	0.518459
O	-5.705262	1.627600	0.433209	-2.800213	3.256304	0.636171	-5.878225	-1.465715	-0.236896
H	-3.637235	2.341996	1.492484	-3.077613	3.124472	-2.084082	-3.416257	-3.102155	-1.135584
H	-3.028775	1.841048	-1.318385	-4.426999	1.375150	-1.197113	-2.940010	-2.050151	0.865174
C	-2.908552	0.054000	-0.113004	-2.811177	0.041134	-0.773046	-2.908692	-0.033018	0.114146
C	-1.406353	0.000076	-0.049971	-1.356693	0.001820	-0.373703	-1.404672	0.000370	0.046942
H	-3.273192	-0.584256	-0.919943	-2.946778	-0.447684	-1.737268	-3.247810	0.469563	1.020323
H	-3.342996	-0.308309	0.820466	-3.415771	-0.490992	-0.040116	-3.343736	0.499579	-0.734478
C	1.396453	0.000599	0.050655	1.347163	0.001384	0.371105	1.396165	-0.000024	-0.047015
C	-0.648661	0.000231	-1.223449	-0.349364	-0.001125	-1.340564	-0.650865	0.000285	1.221294
C	-0.735609	-0.000044	1.173353	-0.985879	-0.000937	0.972523	-0.734045	-0.000857	-1.175602
C	0.655029	-0.000294	1.225536	0.353199	-0.000666	1.343546	0.654911	0.000683	-1.223662
C	0.739141	-0.000567	-1.176124	0.991573	-0.000477	-0.972908	0.738506	-0.000458	1.178043
H	-1.152922	-0.002694	-2.182050	-0.621522	-0.004486	-2.388097	-1.156844	0.005070	2.179298
H	-1.307115	-0.008606	2.092811	-1.754689	-0.007150	1.734538	-1.305383	-0.014254	-2.093303
H	1.156931	-0.004005	2.183851	0.620685	-0.005025	2.391807	1.158914	0.000579	-2.180996
H	1.308878	-0.005101	-2.095728	1.758017	-0.004626	-1.736479	1.306077	0.003765	2.099112
H	2.477264	-0.001813	0.088660	2.389867	-0.000126	0.658508	2.477192	0.002677	-0.084391
O	-5.327377	1.055962	-1.707894	-4.103707	1.572836	1.354186	-5.084006	-1.255181	1.855965
H	-6.292264	0.971747	-1.691535	-4.069775	2.104665	2.162927	-6.031586	-1.180016	2.043118
H	-2.097703	2.407439	0.910973	-1.714459	2.320530	-1.742876	-3.946262	-1.744016	-1.853195

atom	7			8			9		
	x	y	z	x	y	z	x	y	z
N	-4.385920	1.399553	2.293420	-3.122836	-2.282404	-0.667760	-3.174049	-2.329545	-0.471721
C	-3.083975	1.467088	1.638233	-3.399495	-1.495532	0.522080	-3.384054	-1.467932	0.691818
C	-3.112094	2.339643	0.385175	-4.887599	-1.553453	0.820100	-4.857797	-1.457724	1.072264
O	-3.922629	2.225308	-0.500383	-5.673460	-2.301376	0.298524	-5.501374	-0.489663	1.380289
H	-4.652629	2.302070	2.667400	-2.143274	-2.522000	-0.726116	-2.187957	-2.369949	-0.697380
H	-2.362356	1.888960	2.336724	-2.925320	-1.894735	1.433052	-2.867803	-1.836346	1.593920
C	-2.632808	0.044677	1.245732	-2.893228	-0.045443	0.346119	-2.885844	-0.043499	0.412013
C	-1.272270	0.000337	0.598260	-1.397592	-0.000167	0.161640	-1.394482	-0.000723	0.194224
H	-3.384888	-0.370630	0.572173	-3.178060	0.535015	1.222099	-3.164334	0.587196	1.255194
H	-2.647544	-0.550034	2.159030	-3.395436	0.382813	-0.521847	-3.412453	0.335617	-0.463670
C	1.263980	0.000509	-0.594781	1.388238	-0.000430	-0.161076	1.384413	-0.000822	-0.193586
C	-1.137857	-0.000192	-0.790260	-0.549097	0.000180	1.270720	-0.519498	0.000435	1.282987
C	-0.115937	-0.000062	1.379618	-0.827303	-0.000144	-1.111798	-0.852468	0.000247	-1.091620
C	1.142376	-0.000361	0.790997	0.554194	0.000442	-1.273267	0.525307	0.000527	-1.285908
C	0.119708	-0.000231	-1.383834	0.831559	0.000119	1.113781	0.856727	0.000337	1.093902
H	-2.025901	0.003700	-1.409396	-0.975339	0.006975	2.266667	-0.922495	0.009203	2.288457
H	-0.203423	-0.004354	2.459281	-1.473048	-0.005632	-1.979781	-1.517016	0.002745	-1.946003
H	2.027721	-0.004710	1.412743	0.978376	0.003362	-2.268541	0.925997	0.004871	-2.290844
H	0.204709	-0.003930	-2.462374	1.472592	0.005799	1.985304	1.517813	0.006844	1.950303
H	2.242848	-0.002467	-1.054876	2.462617	0.002965	-0.285727	2.455634	0.002617	-0.342702
O	-2.127228	3.262330	0.367227	-5.243718	-0.689657	1.800377	-5.375847	-2.710662	1.080632
H	-2.191889	3.744903	-0.471074	-6.190161	-0.815492	1.961179	-6.304251	-2.637906	1.345933
H	-5.096825	1.133684	1.620574	-3.679412	-3.126326	-0.687954	-3.493034	-3.272062	-0.290951
atom	10			11			12		
	x	y	z	x	y	z	x	y	z
N	2.636643	-2.217460	-2.009531	-3.077580	2.477082	0.662824	-4.442265	1.385796	2.190701
C	3.348507	-1.481110	-0.980168	-3.414714	1.511681	-0.373630	-3.113707	1.442987	1.579148
C	3.422471	-2.122486	0.410071	-4.913534	1.445244	-0.593050	-3.051277	2.460811	0.444216
O	4.027041	-1.648263	1.338915	-5.447006	1.310412	-1.663090	-2.456837	3.506420	0.473711
H	3.017513	-3.147938	-2.119833	-3.499897	2.224434	1.547826	-4.704335	2.280804	2.584993
H	4.392289	-1.385908	-1.290272	-2.995140	1.861912	-1.315896	-2.410180	1.801894	2.329106
C	2.791601	-0.043215	-0.841055	-2.907443	0.056405	-0.134365	-2.690456	0.038298	1.119011
C	1.347097	-0.001713	-0.406927	-1.405960	-0.000270	-0.059570	-1.300458	0.000093	0.534824
H	2.905574	0.434589	-1.813365	-3.267726	-0.573694	-0.950072	-3.416310	-0.323610	0.389445
H	3.415041	0.490127	-0.125191	-3.350910	-0.314665	0.791383	-2.758272	-0.609762	1.993465
C	-1.337912	-0.001349	0.403719	1.396086	0.000441	0.060168	1.291607	0.000444	-0.532066
C	0.316221	0.001078	-1.348723	-0.640359	0.000406	-1.227875	-1.097514	0.000278	-0.844804
C	1.010179	0.000840	0.949047	-0.743567	0.000225	1.168221	-0.183412	-0.000196	1.372042
C	-0.319992	0.000691	1.351462	0.646562	-0.000311	1.230054	1.102008	-0.000073	0.846129
C	-1.015594	0.000454	-0.948578	0.747238	-0.000491	-1.170999	0.187770	-0.000547	-1.376125
H	0.562451	0.005789	-2.402711	-1.138302	-0.002395	-2.189745	-1.953998	0.002851	-1.506431
H	1.798258	0.004223	1.690805	-1.320949	-0.008146	2.084014	-0.323419	-0.004252	2.446146
H	-0.561750	0.007654	2.406003	1.141971	-0.004169	2.191761	1.955619	-0.002658	1.510682
H	-1.800482	0.005822	-1.693183	1.323126	-0.005242	-2.086735	0.325939	-0.002866	-2.449131
H	-2.373262	0.002072	0.716666	2.476611	-0.002442	0.105617	2.291839	-0.000596	-0.943510
O	2.745630	-3.289199	0.501555	-5.604692	1.477684	0.572126	-3.808592	2.080843	-0.619271
H	2.847244	-3.604912	1.411769	-6.544501	1.398083	0.353285	-3.752479	2.787819	-1.278229
H	1.661067	-2.327046	-1.760281	-2.074441	2.510699	0.795429	-5.143818	1.133354	1.504692

atom	13			14			15		
	x	y	z	x	y	z	x	y	z
N	-4.396811	1.554452	2.270750	-3.274511	-2.196894	-0.889264	-4.446539	1.307739	2.277435
C	-3.084703	1.487819	1.637502	-3.456477	-1.471804	0.359892	-3.146349	1.434603	1.604812
C	-3.141677	2.349644	0.387196	-4.894869	-1.389048	0.859525	-3.316267	2.289766	0.341315
O	-3.992165	2.244064	-0.460528	-5.244226	-1.541113	2.001735	-2.421480	2.906839	-0.165348
H	-5.118134	1.300429	1.605373	-3.489769	-3.179368	-0.781225	-4.482618	0.491941	2.874262
H	-2.343635	1.911238	2.314638	-2.904298	-1.988572	1.143846	-2.381890	1.921981	2.213217
C	-2.636920	0.055154	1.233894	-2.900975	-0.031651	0.241552	-2.644803	0.024876	1.212709
C	-1.274037	0.000119	0.594637	-1.401091	0.000396	0.108604	-1.277615	0.000633	0.580644
H	-3.393325	-0.350057	0.559947	-3.204094	0.528764	1.127806	-3.381873	-0.429630	0.547748
H	-2.650794	-0.548499	2.143972	-3.374907	0.446600	-0.618034	-2.629525	-0.573633	2.127799
C	1.265723	0.000396	-0.591001	1.392616	-0.000010	-0.108564	1.270070	0.000379	-0.578163
C	-1.135470	0.000178	-0.793758	-0.596674	0.000238	1.248815	-1.127867	-0.000109	-0.804487
C	-0.119907	-0.000159	1.379294	-0.784786	-0.000841	-1.141683	-0.133153	-0.000658	1.377904
C	1.140153	-0.000099	0.794251	0.600811	0.000648	-1.251482	1.132589	0.000151	0.805712
C	0.123537	-0.000435	-1.383423	0.789124	-0.000430	1.144311	0.135977	-0.000397	-1.381609
H	-2.021652	0.004440	-1.415746	-1.060349	0.003826	2.227962	-2.005915	0.013230	-1.437061
H	-0.209409	-0.003909	2.458880	-1.394894	-0.010338	-2.034697	-0.233069	-0.004229	2.456811
H	2.023685	-0.003475	1.418536	1.062182	0.000427	-2.230060	2.010043	-0.001499	1.438408
H	0.211756	-0.004362	-2.461686	1.396606	0.003397	2.039513	0.234058	0.004106	-2.458694
H	2.245850	-0.002420	-1.048292	2.470949	0.002435	-0.193188	2.254327	0.000961	-1.026484
O	-2.120637	3.222768	0.306119	-5.765772	-1.082959	-0.136328	-4.555675	2.246391	-0.182473
H	-2.204376	3.693706	-0.536977	-6.646594	-1.016474	0.260154	-5.104372	1.759078	0.465186
H	-4.451170	0.916689	3.054635	-3.878426	-1.827686	-1.613223	-4.654856	2.118242	2.846934

atom	16			17			18		
	x	y	z	x	y	z	x	y	z
N	-4.381488	-1.530314	-2.164937	4.498623	1.340778	-2.100458	4.459084	1.543205	-2.157478
C	-3.344827	-1.439272	-1.125428	3.240769	1.423486	-1.343831	3.125204	1.474988	-1.563198
C	-3.877426	-2.117168	0.151447	3.533233	2.270979	-0.110563	3.076705	2.432987	-0.383538
O	-3.508407	-1.844792	1.260900	3.588739	1.891918	1.027714	2.321393	3.357078	-0.256232
H	-5.067922	-0.791273	-2.063031	4.367258	0.784100	-2.935773	5.156105	1.172344	-1.522969
H	-2.518366	-2.074323	-1.455517	2.443485	1.938774	-1.895696	2.409360	1.868196	-2.283249
C	-2.790007	-0.022089	-0.849953	2.752257	0.020833	-0.964129	2.681472	0.051641	-1.134943
C	-1.348861	-0.000214	-0.395511	1.330444	-0.000295	-0.456369	1.295794	0.000084	-0.545492
H	-3.426764	0.473104	-0.116120	3.436500	-0.397106	-0.227532	3.413481	-0.333512	-0.423689
H	-2.870247	0.538841	-1.782927	2.821163	-0.600819	-1.860034	2.734565	-0.575820	-2.027553
C	1.340622	-0.000256	0.393655	-1.321650	0.000072	0.453993	-1.287031	0.000378	0.542485
C	-1.005887	-0.001157	0.956063	1.047090	0.000998	0.909271	1.104071	0.000192	0.835970
C	-0.324414	0.001354	-1.344213	0.263884	-0.000515	-1.357616	0.172270	-0.000129	-1.373602
C	1.010461	-0.001118	-0.956815	-1.051722	0.000626	-0.910292	-1.108801	-0.000101	-0.837058
C	0.328080	0.001391	1.346821	-0.268046	-0.000885	1.361014	-0.176302	-0.000423	1.377697
H	-1.789161	-0.029335	1.699011	1.863761	0.019123	1.616836	1.965664	0.001948	1.491597
H	-0.572638	0.013782	-2.399267	0.465830	-0.009778	-2.422295	0.302607	-0.002698	-2.449023
H	1.789720	0.003309	-1.707313	-1.864158	-0.003725	-1.624788	-1.967893	-0.001823	-1.494494
H	0.576293	0.000150	2.399794	-0.468947	-0.001810	2.424119	-0.305725	-0.001836	2.451727
H	2.377917	0.001019	0.700247	-2.344348	-0.002420	0.806333	-2.283813	-0.000170	0.962094
O	-4.786237	-3.076622	-0.087009	3.760251	3.560864	-0.466031	4.006476	2.107485	0.552740
H	-4.988126	-3.024651	-1.043282	3.981815	4.050366	0.339820	3.937661	2.759422	1.265114
H	-3.983060	-1.449910	-3.090678	4.804345	2.261442	-2.390197	4.496325	1.002999	-3.012574

atom	19			20			21		
	x	y	z	x	y	z	x	y	z
N	-4.579931	1.342750	1.938242	4.295836	-1.189068	2.577350	2.699986	-2.009577	-2.175599
C	-3.206684	1.410213	1.421201	3.053566	-1.362933	1.845550	3.421718	-1.413504	-1.052335
C	-3.213419	2.484462	0.337366	3.110629	-2.519814	0.828989	3.556776	-2.354414	0.156435
O	-2.955649	3.642276	0.547095	4.136552	-3.022925	0.462107	4.614311	-2.775686	0.538528
H	-4.657762	0.610121	2.632953	4.512857	-2.028389	3.101516	3.142506	-2.869358	-2.474649
H	-2.484359	1.765524	2.163750	2.266724	-1.596708	2.568006	4.448552	-1.251237	-1.375663
C	-2.765297	0.027352	0.923287	2.681033	-0.032077	1.146469	2.837987	-0.026799	-0.690060
C	-1.336001	0.000286	0.440052	1.297923	-0.000507	0.545987	1.370788	-0.004908	-0.330715
H	-3.448224	-0.298065	0.140578	3.430085	0.177899	0.380391	3.003159	0.607651	-1.561345
H	-2.877956	-0.667482	1.760254	2.777509	0.742151	1.908201	3.427632	0.385478	0.129974
C	1.326849	0.000576	-0.437907	-1.287386	-0.000784	-0.543490	-1.358895	-0.003213	0.328544
C	-1.034621	0.000615	-0.921420	1.104871	-0.000886	-0.835673	0.391222	0.003254	-1.329830
C	-0.281078	-0.000760	1.354761	0.170919	0.001262	1.374364	0.954151	0.002592	1.003866
C	1.039207	0.000329	0.923005	-1.110644	-0.000617	0.836427	-0.397673	0.001478	1.332148
C	0.285645	-0.001046	-1.358491	-0.175683	0.001533	-1.377614	-0.959593	0.000797	-1.004014
H	-1.841721	0.007667	-1.641265	1.965203	-0.012399	-1.492187	0.696427	0.018211	-2.367593
H	-0.496382	-0.007524	2.416483	0.300209	0.014546	2.449797	1.695609	0.008613	1.792565
H	1.842778	-0.002436	1.647291	-1.969838	0.004277	1.493520	-0.696889	0.008190	2.371439
H	0.500162	-0.003850	-2.418902	-0.303626	-0.000245	-2.451533	-1.701134	0.008880	-1.791536
H	2.353929	-0.000668	-0.776882	-2.283615	-0.001345	-0.963987	-2.410178	-0.002155	0.581852
O	-3.588320	2.026238	-0.875175	1.925244	-2.969304	0.355581	2.412084	-2.753366	0.764915
H	-3.608333	2.786824	-1.474271	1.196913	-2.402819	0.645274	1.651272	-2.241330	0.456498
H	-4.837327	2.214740	2.384728	5.061333	-1.074973	1.921283	1.737321	-2.222053	-1.945878

Table S20. Tables with the optimized Cartesian coordinates Tyr conformers calculated at the B3LYP/D3/aug-cc-pVTZ .

atom	1			2			3		
	x	y	z	x	y	z	x	y	z
N	-2.685071	2.488968	-1.490233	-2.692296	2.491724	-1.473848	-3.126155	2.434616	0.444840
C	-3.414908	1.473865	-0.720851	-3.422561	1.462678	-0.723615	-3.448367	1.430081	-0.574887
C	-3.598625	1.957902	0.732927	-3.627035	1.928394	0.733403	-4.970305	1.317250	-0.740971
O	-3.996149	1.244618	1.616467	-4.030408	1.201672	1.602994	-5.482991	0.770961	-1.680195
H	-1.690836	2.290141	-1.503254	-1.696847	2.298731	-1.479435	-2.240307	2.232126	0.891200
H	-4.432173	1.424761	-1.116935	-4.435104	1.410274	-1.131147	-3.045558	1.677031	-1.559923
C	-2.817584	0.050168	-0.764326	-2.814132	0.044021	-0.777915	-2.912481	0.040162	-0.167846
C	-1.363067	0.001829	-0.367398	-1.360776	0.001206	-0.376063	-1.409077	0.000950	-0.074716
H	-2.936052	-0.333167	-1.779091	-2.926079	-0.330739	-1.796643	-3.272938	-0.673853	-0.907826
H	-3.415409	-0.576493	-0.103794	-3.409799	-0.593164	-0.125464	-3.355245	-0.235957	0.791529
C	1.348926	0.001823	0.362590	1.346892	0.001228	0.370233	1.394666	0.001105	0.076041
C	-0.352698	-0.001049	-1.331716	-0.982521	-0.000636	0.971552	-0.622634	-0.000255	-1.230770
C	-0.981508	-0.000815	0.976345	-0.349501	-0.000580	-1.335489	-0.756335	-0.000631	1.155615
C	0.357461	-0.001010	1.341343	0.994481	-0.000638	-0.975142	0.633007	-0.000399	1.239210
C	0.990886	-0.000778	-0.981164	0.351425	-0.000580	1.344909	0.760374	-0.000769	-1.165380
H	-0.619103	-0.018457	-2.381632	-1.747842	0.000005	1.735462	-1.103754	-0.007560	-2.200938
H	-1.742334	0.001544	1.744622	-0.609625	-0.017741	-2.386891	-1.337314	-0.014355	2.069516
H	0.632206	-0.005298	2.389490	1.763234	-0.009425	-1.738635	1.119514	-0.007127	2.207198
H	1.767402	-0.009359	-1.732864	0.638868	-0.005285	2.386771	1.364366	-0.008905	-2.061629
O	2.683362	0.001244	0.660461	2.645772	-0.001071	0.797278	2.761351	-0.000408	0.081644
H	2.807571	0.000162	1.614627	3.241968	-0.008182	0.042200	3.089023	-0.012998	0.986241
H	-3.005421	2.544778	-2.446397	-3.003905	2.556693	-2.432323	-3.056538	3.361149	0.043899
O	-3.313459	3.251052	0.917341	-3.354469	3.221516	0.936378	-5.674853	1.839525	0.274301
H	-3.001252	3.572915	0.042595	-3.033544	3.556494	0.070058	-5.010394	2.236840	0.877096

atom	4			5			6		
	x	y	z	x	y	z	x	y	z
N	-2.763922	2.188019	-1.900189	-2.759726	2.197734	-1.881867	-3.159079	-2.460457	-0.407211
C	-3.409045	1.474399	-0.814585	-3.408205	1.479294	-0.801998	-3.390423	-1.481303	0.662820
C	-3.350604	2.220268	0.519657	-3.352164	2.216259	0.537242	-4.879900	-1.477988	1.056530
O	-2.778619	3.259934	0.715029	-2.774651	3.250720	0.743749	-5.376934	-0.624435	1.740886
H	-3.155096	3.117312	-1.992723	-3.146051	3.129546	-1.968622	-3.254044	-2.028513	-1.319229
H	-4.471843	1.372284	-1.051139	-4.470656	1.379933	-1.041347	-2.870267	-1.847053	1.551846
C	-2.839302	0.040608	-0.678486	-2.840745	0.043871	-0.671971	-2.893934	-0.054855	0.365894
C	-1.372310	0.001677	-0.329940	-1.373650	0.002004	-0.324217	-1.401681	-0.001175	0.171559
H	-3.009505	-0.452184	-1.635300	-3.011308	-0.444706	-1.630856	-3.205966	0.586322	1.189126
H	-3.418590	-0.489753	0.075933	-3.421144	-0.488713	0.080044	-3.407242	0.311358	-0.527062
C	1.357940	0.001508	0.326025	1.359082	0.001839	0.321197	1.386873	-0.001334	-0.170558
C	-0.947502	-0.000714	1.001215	-0.394936	-0.001200	-1.321199	-0.828547	0.000439	-1.100596
C	-0.394757	-0.001075	-1.324248	-0.948704	-0.000922	1.004008	-0.541988	0.000675	1.270068
C	0.958485	-0.000518	-1.007041	0.401154	-0.001000	1.331142	0.837280	0.000578	1.109578
C	0.398145	-0.000877	1.333989	0.957055	-0.000720	-1.010931	0.548063	0.000816	-1.280051
H	-1.685479	-0.006184	1.793003	-0.698544	-0.003969	-2.359950	-1.469261	0.015350	-1.973642
H	-0.694425	-0.004199	-2.364084	-1.683547	-0.006301	1.798656	-0.954975	0.009674	2.271312
H	1.701649	-0.005985	-1.795644	0.709170	-0.006005	2.370000	1.485936	0.009080	1.977461
H	0.720765	-0.005784	2.365506	1.709904	-0.005716	-1.786435	0.984920	0.009396	-2.268600
O	2.672121	-0.001073	0.706543	2.703510	0.000208	0.574900	2.735264	0.000549	-0.398405
H	3.239267	0.003306	-0.070548	2.858894	0.009717	1.524264	3.211703	0.013816	0.437355
H	-1.780723	2.318414	-1.689627	-1.775256	2.320633	-1.672947	-2.224539	-2.842466	-0.360060
O	-4.052794	1.578643	1.488258	-4.064200	1.572674	1.497929	-5.567461	-2.533951	0.599377
H	-3.987225	2.113484	2.293045	-4.002376	2.103848	2.305382	-4.927031	-3.053672	0.067766

atom	7			8			9		
	x	y	z	x	y	z	x	y	z
N	-3.137718	-2.492179	-0.307129	-2.714140	2.212931	-1.918865	-2.701240	2.225495	-1.917444
C	-3.390603	-1.462672	0.709688	-3.389383	1.476783	-0.864829	-3.385351	1.478415	-0.876574
C	-4.886386	-1.446464	1.078118	-3.417660	2.121921	0.525364	-3.424440	2.108516	0.519891
O	-5.397449	-0.562972	1.712228	-3.957334	1.632176	1.485865	-3.996768	1.621804	1.462684
H	-3.211648	-2.103943	-1.240497	-3.095968	3.144622	-2.014297	-3.085128	3.156882	-2.008228
H	-2.883519	-1.781369	1.623898	-4.443366	1.380152	-1.139348	-4.436539	1.382635	-1.160777
C	-2.896036	-0.050352	0.349263	-2.823608	0.041450	-0.742287	-2.817834	0.042542	-0.763397
C	-1.402910	-0.000924	0.160711	-1.365053	0.001667	-0.358770	-1.361678	0.001669	-0.371348
H	-3.214715	0.627696	1.139942	-2.970833	-0.438791	-1.709048	-2.958780	-0.429165	-1.735293
H	-3.405128	0.272604	-0.562551	-3.421532	-0.493031	-0.005790	-3.419429	-0.499351	-0.035207
C	1.387989	-0.001112	-0.160945	1.350771	0.001362	0.354944	1.347662	0.001432	0.366524
C	-0.546254	0.000761	1.265307	-0.361468	-0.001101	-1.330710	-0.978037	-0.000866	0.973226
C	-0.825588	0.000086	-1.106270	-0.974421	-0.000748	0.980942	-0.354513	-0.000951	-1.335546
C	0.555541	0.000932	-1.275068	0.367421	-0.000765	1.340425	0.989001	-0.000600	-0.977924
C	0.831223	0.000258	1.116265	0.982752	-0.000414	-0.986829	0.357564	-0.000684	1.345069
H	-0.966354	0.010683	2.263676	-0.638570	-0.006196	-2.376951	-1.740436	-0.004424	1.741287
H	-1.460046	0.013384	-1.983835	-1.730113	-0.003034	1.755414	-0.622387	-0.004896	-2.384119
H	0.981925	0.008690	-2.271099	0.649331	-0.008373	2.386793	1.755624	-0.006965	-1.743788
H	1.488395	0.008920	1.974311	1.754828	-0.007090	-1.743225	0.648952	-0.008634	2.385921
O	2.752432	0.000902	-0.251692	2.688674	-0.001246	0.643080	2.650092	-0.000824	0.786791
H	3.022475	0.015304	-1.175016	2.819114	-0.009701	1.596207	3.240359	-0.008975	0.027150
H	-2.205041	-2.871877	-0.220864	-1.729992	2.319412	-1.704132	-1.720223	2.333648	-1.689567
O	-5.563094	-2.525350	0.659817	-2.783060	3.314943	0.578829	-2.755261	3.280710	0.601361
H	-4.911986	-3.067119	0.164363	-2.857798	3.634102	1.490415	-2.832846	3.587815	1.516882

atom	10			11			12		
	x	y	z	x	y	z	x	y	z
N	-3.079237	2.483498	0.648094	-4.509644	1.402384	2.051071	-4.515029	1.408236	2.036601
C	-3.436439	1.501034	-0.358383	-3.170396	1.466607	1.474038	-3.176241	1.473115	1.459704
C	-4.945629	1.423728	-0.455727	-3.123699	2.343484	0.224835	-3.130672	2.333123	0.198857
O	-5.709867	1.622021	0.453295	-3.859483	2.212454	-0.721898	-3.884874	2.206798	-0.733688
H	-3.635899	2.353777	1.484487	-4.797343	2.305277	2.408170	-4.807882	2.314374	2.381133
H	-3.050841	1.826159	-1.325966	-2.490969	1.885429	2.215368	-2.498819	1.904150	2.195973
C	-2.912987	0.051793	-0.103642	-2.699737	0.045530	1.103531	-2.697711	0.048849	1.108241
C	-1.410843	-0.000185	-0.044063	-1.307000	0.000038	0.529380	-1.305068	0.000573	0.534262
H	-3.280612	-0.595269	-0.902409	-3.415295	-0.367341	0.389744	-3.411593	-0.377203	0.400443
H	-3.343544	-0.304080	0.834300	-2.766009	-0.552472	2.012593	-2.761882	-0.536580	2.025552
C	1.396054	0.000481	0.045217	1.293625	0.000464	-0.524867	1.292067	0.000919	-0.528835
C	-0.649074	0.000293	-1.216158	-0.188293	0.000178	1.365256	-1.089714	-0.000357	-0.844992
C	-0.731053	0.000231	1.171953	-1.091837	-0.000002	-0.847083	-0.188397	-0.000050	1.368113
C	0.659236	-0.000379	1.224694	0.193573	-0.000251	-1.376555	1.100296	-0.000694	0.850253
C	0.735680	-0.000440	-1.181644	1.099931	-0.000428	0.853869	0.190816	-0.000390	-1.378802
H	-1.150661	-0.002497	-2.176036	-0.327123	-0.003839	2.439413	-1.940299	0.003803	-1.514516
H	-1.292491	-0.007695	2.097424	-1.940218	0.005367	-1.519186	-0.323591	-0.003753	2.442663
H	1.167024	-0.005366	2.181813	0.339071	-0.004194	-2.450378	1.955055	-0.005854	1.516274
H	1.319738	-0.005411	-2.091041	1.962632	-0.005545	1.504959	0.352264	-0.004970	-2.447525
O	2.763448	-0.002644	0.022857	2.584525	-0.002275	-0.979948	2.534270	-0.000894	-1.103754
H	3.108117	-0.004017	0.921108	2.593158	-0.009820	-1.941915	3.212003	-0.003550	-0.421014
H	-2.100332	2.408709	0.892020	-5.180031	1.134861	1.338535	-5.182986	1.129588	1.325975
O	-5.351018	1.031469	-1.685835	-2.168472	3.295473	0.286803	-2.149567	3.259828	0.231058
H	-6.315369	0.944247	-1.658643	-2.187282	3.784606	-0.549959	-2.164637	3.732668	-0.615131

atom	13			14			15		
	x	y	z	x	y	z	x	y	z
N	-3.286970	-2.050861	-1.267714	3.118230	-2.279800	0.701890	-3.296755	-2.030537	-1.277473
C	-3.481533	-1.479907	0.052771	3.414237	-1.494145	-0.484587	-3.486176	-1.474555	0.049749
C	-4.946473	-1.430695	0.475210	4.907198	-1.547483	-0.755641	-4.949730	-1.425712	0.477421
O	-5.887146	-1.442916	-0.277102	5.688832	-2.284580	-0.212385	-5.893020	-1.426828	-0.271526
H	-3.431908	-3.052225	-1.254247	2.138904	-2.524458	0.739225	-3.448090	-3.031008	-1.276826
H	-2.944245	-2.083916	0.784737	2.956901	-1.896538	-1.402621	-2.948911	-2.088960	0.773090
C	-2.912038	-0.037465	0.117202	2.900109	-0.045336	-0.319633	-2.911790	-0.034880	0.129912
C	-1.408913	0.000348	0.047987	1.403281	0.000006	-0.148381	-1.408661	0.000377	0.056698
H	-3.249536	0.430051	1.042632	3.193682	0.532904	-1.194379	-3.245691	0.422615	1.061624
H	-3.350900	0.528474	-0.707859	3.393913	0.388320	0.550694	-3.351015	0.542247	-0.687217
C	1.395107	-0.000280	-0.047854	-1.388869	-0.000282	0.147725	1.394851	-0.000176	-0.055658
C	-0.645734	0.000269	1.216801	0.557359	0.000152	-1.260264	-0.736428	-0.000938	-1.166772
C	-0.734746	-0.000937	-1.171204	0.816284	-0.000302	1.115465	-0.643771	0.000276	1.221180
C	0.653200	0.000899	-1.225708	-0.565583	0.000439	1.269531	0.745568	-0.000372	1.174957
C	0.741087	-0.000299	1.179979	-0.822472	-0.000012	-1.124076	0.648442	0.000833	-1.230405
H	-1.144352	0.004112	2.178482	0.986373	0.006590	-2.254901	-1.307776	-0.015076	-2.084089
H	-1.300685	-0.015834	-2.091865	1.447679	-0.006630	1.993773	-1.136688	0.004391	2.185711
H	1.159237	0.001118	-2.184013	-1.001566	0.004028	2.261611	1.321388	0.004523	2.092929
H	1.327006	0.004473	2.088201	-1.472018	0.006504	-1.987974	1.165208	0.001469	-2.179738
O	2.764477	0.002730	-0.034679	-2.755104	0.002986	0.229399	2.759188	0.003223	-0.173881
H	3.099911	0.001820	-0.936287	-3.028844	0.011184	1.151656	3.163369	0.004002	0.699046
H	-3.971773	-1.667552	-1.910170	3.678255	-3.121082	0.734635	-3.978186	-1.634256	-1.915547
O	-5.082378	-1.330394	1.819145	5.275565	-0.691071	-1.738095	-5.081070	-1.340357	1.823180
H	-6.028787	-1.258804	2.013478	6.225633	-0.812051	-1.880055	-6.026747	-1.268539	2.020956

atom	16			17			18		
	x	y	z	x	y	z	x	y	z
N	3.132945	-2.256065	0.739724	-4.533569	1.387631	2.010843	-4.549555	1.390144	1.970429
C	3.417923	-1.495704	-0.465437	-3.174605	1.441941	1.469829	-3.194231	1.446356	1.420415
C	4.909392	-1.549779	-0.745150	-3.045036	2.469878	0.350031	-3.083537	2.451623	0.278285
O	5.697007	-2.273008	-0.192136	-2.445261	3.510622	0.420896	-2.496807	3.501141	0.322347
H	2.154585	-2.501857	0.791594	-4.812160	2.281156	2.397071	-4.834367	2.288356	2.340885
H	2.956506	-1.919857	-1.371725	-2.509131	1.789331	2.259063	-2.527656	1.817120	2.197952
C	2.899221	-0.045416	-0.329818	-2.734686	0.039940	1.017693	-2.742685	0.039558	0.995348
C	1.402569	-0.000264	-0.155940	-1.323230	0.000230	0.488321	-1.328101	0.000134	0.474735
H	3.189179	0.515203	-1.217080	-3.431280	-0.314973	0.256625	-3.433650	-0.334124	0.238087
H	3.393654	0.407830	0.530137	-2.842617	-0.615138	1.883075	-2.849446	-0.600130	1.872236
C	-1.388236	-0.000436	0.154227	1.309715	0.000893	-0.483801	1.314272	0.000780	-0.471100
C	0.816730	-0.000001	1.111663	-1.059516	0.000325	-0.881850	-0.244398	-0.000057	1.356122
C	0.556114	0.000183	-1.263908	-0.235628	-0.000225	1.360563	-1.055715	0.000244	-0.891008
C	-0.825778	0.000167	-1.119384	1.069827	-0.000338	0.887986	0.250454	-0.000701	-1.367302
C	-0.561398	0.000351	1.273342	0.238832	-0.000885	-1.371219	1.063487	-0.000400	0.898554
H	1.452622	-0.005008	1.986782	-1.886286	0.003460	-1.580091	-0.427113	-0.003809	2.423676
H	0.980056	0.006663	-2.260629	-0.407571	-0.004033	2.429839	-1.875575	0.002873	-1.597288
H	-1.464989	0.006797	-1.994391	1.900775	-0.003684	1.583410	0.440471	-0.003790	-2.434109
H	-1.009735	0.004557	2.256851	0.437584	-0.003547	-2.433614	1.898433	-0.003561	1.584820
O	-2.740253	0.002873	0.367295	2.570829	-0.001175	-1.014886	2.622618	-0.001594	-0.871842
H	-3.205717	0.009337	-0.474595	3.224495	0.010680	-0.309151	2.671823	0.007306	-1.832564
H	3.695591	-3.094822	0.787446	-5.199326	1.143163	1.287293	-5.217323	1.124748	1.256199
O	5.268671	-0.713063	-1.747990	-3.747079	2.105713	-0.756004	-3.788587	2.055907	-0.815749
H	6.218278	-0.833986	-1.893043	-3.649330	2.817635	-1.404708	-3.707873	2.758365	-1.476926

atom	19			20			21		
	x	y	z	x	y	z	x	y	z
N	-3.085086	2.472711	0.668548	-3.082140	2.481181	0.650159	4.525971	1.562469	-2.010590
C	-3.424088	1.510935	-0.371123	-3.426762	1.504803	-0.374117	3.176932	1.493766	-1.459482
C	-4.922802	1.442416	-0.587128	-4.926715	1.432698	-0.579899	3.156487	2.347612	-0.203043
O	-5.458593	1.306598	-1.656153	-5.469412	1.282651	-1.643385	3.954304	2.238337	0.694102
H	-3.508029	2.217286	1.552546	-3.499115	2.237165	1.540210	5.204331	1.307491	-1.301728
H	-3.007650	1.865914	-1.313143	-3.015983	1.846777	-1.323352	2.479337	1.922402	-2.178435
C	-2.911432	0.056500	-0.139108	-2.912067	0.054207	-0.123761	2.702741	0.059933	-1.092431
C	-1.410164	-0.000242	-0.063222	-1.410533	-0.000525	-0.053045	1.307299	0.000382	-0.528912
H	-3.272681	-0.571441	-0.956239	-3.275687	-0.585362	-0.930806	3.420503	-0.352462	-0.381322
H	-3.354362	-0.320085	0.784882	-3.351383	-0.309798	0.806990	2.768470	-0.537527	-2.004585
C	1.395448	0.000499	0.063200	1.395770	0.000283	0.054079	-1.294308	0.000884	0.523388
C	-0.741398	0.000250	1.162001	-0.641362	0.000429	-1.220285	1.086093	0.000006	0.849671
C	-0.638013	0.000369	-1.225116	-0.738507	0.000510	1.167186	0.194062	-0.000142	-1.367335
C	0.749154	-0.000497	-1.171010	0.651329	-0.000389	1.228933	-1.096837	-0.000491	-0.854767
C	0.644973	-0.000379	1.234148	0.743303	-0.000309	-1.176869	-0.196308	-0.000639	1.377955
H	-1.314654	-0.007924	2.080276	-1.137026	-0.002358	-2.183222	1.933829	0.004646	1.522981
H	-1.127009	-0.002193	-2.191386	-1.305451	-0.007219	2.089313	0.332615	-0.003170	-2.441534
H	1.329306	-0.005825	-2.086136	1.153096	-0.005542	2.189245	-1.948784	-0.004722	-1.524327
H	1.156881	-0.005550	2.186015	1.332998	-0.005542	-2.082613	-0.362295	-0.005241	2.445975
O	2.757598	-0.002479	0.185886	2.763327	-0.003096	0.040726	-2.538408	-0.001035	1.093562
H	3.167217	-0.004463	-0.684657	3.102141	-0.007022	0.941184	-3.213942	0.000293	0.408630
H	-2.081718	2.500766	0.801569	-2.077896	2.511388	0.775899	4.628888	0.924077	-2.789170
O	-5.612188	1.472311	0.579343	-5.608738	1.477575	0.590708	2.129756	3.217859	-0.177067
H	-6.552037	1.389755	0.361758	-6.549858	1.391941	0.379938	2.161038	3.681611	0.673569

atom	22			23			24		
	x	y	z	x	y	z	x	y	z
N	-3.184283	-2.312809	-0.521009	-2.757983	2.308006	-1.960425	4.593759	1.332748	-1.957261
C	-3.401514	-1.468373	0.653584	-3.370278	1.510986	-0.910271	3.259250	1.451971	-1.353471
C	-4.878321	-1.456640	1.021231	-3.308540	2.197617	0.444627	3.368897	2.254760	-0.050303
O	-5.517883	-0.491208	1.345888	-3.974888	1.859069	1.391227	2.454920	2.862750	0.433210
H	-2.195909	-2.354714	-0.736130	-1.774778	2.464649	-1.776656	4.654142	0.537563	-2.579413
H	-2.895859	-1.853855	1.554849	-4.435253	1.419896	-1.130981	2.535774	1.972100	-1.984351
C	-2.891632	-0.042747	0.401403	-2.819588	0.059073	-0.742802	2.719225	0.034692	-1.043598
C	-1.399252	-0.000834	0.191052	-1.364999	0.001988	-0.361322	1.316275	0.001068	-0.499196
H	-3.173672	0.575598	1.252571	-2.980544	-0.450134	-1.695041	3.411077	-0.451451	-0.352553
H	-3.411767	0.354557	-0.470295	-3.430631	-0.447639	0.004758	2.757669	-0.530641	-1.978965
C	1.384281	-0.000830	-0.189254	1.350346	0.001892	0.357181	-1.303781	0.001496	0.495426
C	-0.843922	0.000400	-1.090099	-0.359247	-0.001309	-1.330301	0.218522	-0.000711	-1.360593
C	-0.524327	0.000450	1.277294	-0.975694	-0.000763	0.978588	1.072452	-0.000157	0.871434
C	0.853000	0.000381	1.098087	0.364906	-0.001176	1.340875	-0.222460	-0.001124	1.371406
C	0.530221	0.000433	-1.287080	0.984687	-0.000632	-0.985021	-1.081008	-0.000571	-0.878477
H	-1.500183	0.003370	-1.950770	-0.630397	-0.016012	-2.378223	0.379880	-0.003570	-2.431899
H	-0.923260	0.009059	2.284194	-1.732311	-0.000787	1.752624	1.903261	0.014173	1.564509
H	1.513887	0.007914	1.956845	0.644700	-0.008622	2.387753	-0.391226	0.003629	2.441481
H	0.953476	0.006153	-2.281605	1.758032	-0.008883	-1.740054	-1.929029	-0.002930	-1.548413
O	2.730582	0.001952	-0.435233	2.687454	-0.000757	0.647384	-2.603210	0.000981	0.922832
H	3.216635	0.010152	0.394923	2.816896	-0.006534	1.600697	-2.632113	0.027406	1.884122
H	-3.510157	-3.256365	-0.359030	-2.836425	1.836312	-2.852193	4.842147	2.159694	-2.485486
O	-5.405936	-2.705632	0.997555	-2.393125	3.184436	0.511647	4.577134	2.173149	0.538428
H	-6.335897	-2.631254	1.256884	-2.400263	3.519075	1.420425	5.154345	1.705109	-0.098909

atom	25			26			27		
	x	y	z	x	y	z	x	y	z
N	-3.293128	-2.140307	-1.015134	4.602340	1.311998	-1.957589	-4.494807	-1.557133	-1.915465
C	-3.467120	-1.480102	0.271326	3.255030	1.439436	-1.385142	-3.411984	-1.444272	-0.925607
C	-4.902828	-1.418580	0.780023	3.330292	2.285942	-0.107399	-3.896404	-2.073092	0.394721
O	-5.248177	-1.622296	1.915465	2.401874	2.904486	0.332828	-3.474503	-1.772668	1.478195
H	-3.505722	-3.127518	-0.955159	4.681956	0.497638	-2.552163	-5.165060	-0.803646	-1.811304
H	-2.913182	-2.038304	1.024974	2.540671	1.933231	-2.046975	-2.607775	-2.100310	-1.269332
C	-2.905724	-0.037336	0.224665	2.720122	0.028382	-1.042042	-2.829142	-0.025718	-0.723793
C	-1.406046	0.000489	0.099947	1.315552	0.000365	-0.501208	-1.369596	-0.000547	-0.334826
H	-3.212830	0.481667	1.134475	3.410962	-0.436160	-0.335343	-3.429394	0.501732	0.018670
H	-3.377051	0.482142	-0.612474	2.766366	-0.560458	-1.962564	-2.947501	0.504261	-1.670846
C	1.392295	-0.000235	-0.099630	-1.303381	0.000732	0.496747	1.356227	-0.000732	0.332028
C	-0.600364	0.000169	1.239991	1.068576	0.000420	0.871487	-0.382230	0.001367	-1.322432
C	-0.777396	-0.001028	-1.142737	0.221590	-0.000604	-1.363086	-0.960128	-0.000901	0.997107
C	0.607760	0.000897	-1.249260	-1.079410	0.000052	-0.877296	0.387750	0.001534	1.331967
C	0.783751	-0.000292	1.151688	-0.222928	-0.000965	1.373356	0.967977	-0.000721	-1.003844
H	-1.063384	0.002679	2.219285	1.900427	0.014511	1.563578	-0.670987	0.013034	-2.366943
H	-1.375815	-0.012302	-2.043183	0.381678	-0.003961	-2.434505	-1.703682	-0.028565	1.779607
H	1.077905	0.001114	-2.225641	-1.917673	-0.003042	-1.563765	0.686005	-0.000119	2.373806
H	1.402847	0.004004	2.037612	-0.409896	0.003960	2.437610	1.727110	0.004774	-1.773171
O	2.761085	0.002949	-0.136472	-2.558078	-0.002198	1.041749	2.698511	0.000034	0.599858
H	3.063655	-0.000347	-1.049630	-3.219792	0.019661	0.343851	2.841923	-0.002613	1.551200
H	-3.905962	-1.736830	-1.712962	4.855929	2.123636	-2.506600	-4.135827	-1.515980	-2.859742
O	-5.777656	-1.063389	-0.196441	4.525195	2.232760	0.510930	-4.829035	-3.024575	0.228083
H	-6.656047	-1.012306	0.207631	5.120195	1.747801	-0.096327	-5.071843	-2.999270	-0.719987
atom	28			29			30		
	x	y	z	x	y	z	x	y	z
N	-4.494369	-1.548963	-1.928865	4.582921	1.547408	-1.893476	4.561268	1.542984	-1.950128
C	-3.415876	-1.437873	-0.934048	3.218859	1.479578	-1.370755	3.192972	1.472284	-1.439019
C	-3.910281	-2.058867	0.386225	3.114052	2.421066	-0.181920	3.060440	2.451935	-0.284210
O	-3.494685	-1.753711	1.470533	2.357350	3.347154	-0.078597	2.290975	3.371329	-0.223857
H	-5.163430	-0.793935	-1.828059	5.242635	1.159958	-1.229599	5.219746	1.192444	-1.264815
H	-2.613531	-2.099565	-1.271244	2.544218	1.887670	-2.121761	2.520573	1.844834	-2.210274
C	-2.826576	-0.021445	-0.735234	2.743890	0.054306	-0.985744	2.734903	0.053951	-1.011065
C	-1.367787	-0.000261	-0.342703	1.328476	0.000220	-0.473078	1.323084	0.000191	-0.488106
H	-3.425914	0.511003	0.004372	3.435007	-0.344404	-0.241475	3.433101	-0.315535	-0.258506
H	-2.940745	0.506089	-1.684184	2.844804	-0.562668	-1.881768	2.838809	-0.587973	-1.889187
C	1.354606	-0.000383	0.339002	-1.314735	0.000953	0.469273	-1.309667	0.000945	0.483431
C	-0.960535	-0.001104	0.992845	0.246275	0.000024	-1.355864	1.059033	0.000331	0.882242
C	-0.379992	0.001306	-1.326125	1.054211	0.000122	0.892549	0.235998	-0.000146	-1.360618
C	0.970918	-0.000977	-0.998457	-0.252000	-0.000709	1.367036	-1.069536	-0.000423	-0.888199
C	0.382789	0.001419	1.335439	-1.062226	-0.000610	-0.899916	-0.238912	-0.000898	1.371251
H	-1.708322	-0.027355	1.771391	0.429518	-0.002119	-2.423423	1.885299	0.002849	1.581697
H	-0.663020	0.013735	-2.372117	1.872728	0.002025	1.601013	0.407288	-0.002264	-2.430084
H	1.721738	0.005006	-1.779756	-0.443396	-0.002884	2.433526	-1.900381	-0.003028	-1.583704
H	0.693831	0.000461	2.370575	-1.896347	-0.002939	-1.587163	-0.437951	-0.002585	2.433543
O	2.665196	0.002100	0.733938	-2.623409	-0.001474	0.868293	-2.570586	-0.001477	1.014469
H	3.240533	-0.002030	-0.037004	-2.674083	0.015047	1.828844	-3.224456	0.016522	0.309060
H	-4.131020	-1.508903	-2.871521	4.660486	1.019744	-2.753731	4.657069	0.983547	-2.788219
O	-4.844079	-3.009363	0.218339	3.993358	2.077695	0.796550	3.929673	2.152810	0.716784
H	-5.081654	-2.988144	-0.730959	3.892915	2.722484	1.511601	3.807903	2.816424	1.411074

atom	31			32			33		
	x	y	z	x	y	z	x	y	z
N	4.583979	1.345732	-1.923818	4.582542	1.349929	-1.925888	4.415299	-1.169155	2.392416
C	3.294835	1.425866	-1.220884	3.297281	1.427523	-1.215939	3.152055	-1.356112	1.700705
C	3.536893	2.265022	0.028530	3.550054	2.249634	0.042855	3.191532	-2.505369	0.675284
O	3.545523	1.879081	1.166130	3.569455	1.847872	1.174578	4.210141	-3.007769	0.287131
H	4.488952	0.787483	-2.763027	4.480900	0.804805	-2.772877	4.669267	-2.014358	2.889883
H	2.522761	1.946603	-1.802742	2.524900	1.959140	-1.787478	2.393232	-1.608103	2.446913
C	2.786737	0.022462	-0.871515	2.783847	0.022190	-0.881716	2.734080	-0.025660	1.026543
C	1.349168	-0.000202	-0.411936	1.347071	-0.000141	-0.419192	1.325707	-0.000697	0.487846
H	3.444731	-0.405682	-0.116822	3.441438	-0.417121	-0.133111	3.446197	0.196325	0.228953
H	2.886051	-0.591396	-1.770164	2.879189	-0.581751	-1.787473	2.860364	0.745190	1.787484
C	-1.335271	0.000234	0.408452	-1.333406	0.000248	0.414845	-1.310645	-0.000644	-0.484498
C	0.307172	-0.000509	-1.342088	1.013991	0.000853	0.936595	0.230707	0.001427	1.359388
C	1.014253	0.000931	0.940519	0.305244	-0.000514	-1.345529	1.064115	-0.000687	-0.881244
C	-0.312996	-0.000943	1.352546	-1.024530	0.000456	-0.942299	-0.236760	0.001353	-1.369818
C	-1.022326	0.000488	-0.947493	-0.308370	-0.000903	1.355579	-1.073124	-0.000752	0.888325
H	0.538019	-0.009333	-2.400732	1.804866	0.018069	1.672764	0.401708	0.013736	2.428878
H	1.801486	0.019746	1.680510	0.530709	-0.009642	-2.405251	1.888734	-0.011067	-1.581943
H	-0.552368	-0.001296	2.409522	-1.818274	-0.005047	-1.680047	-0.414768	-0.000683	-2.438323
H	-1.823950	-0.004760	-1.672490	-0.561160	-0.002160	2.406500	-1.914594	0.005026	1.566435
O	-2.661167	-0.001727	0.750608	-2.620912	-0.002188	0.881346	-2.612566	-0.001368	-0.895879
H	-2.751190	-0.006185	1.708407	-3.237012	-0.003558	0.142559	-2.657220	-0.003594	-1.857139
H	4.899318	2.266717	-2.201998	4.902882	2.272944	-2.191279	5.154687	-1.024240	1.712949
O	3.779896	3.556909	-0.308471	3.789744	3.546334	-0.278658	1.997190	-2.949876	0.219522
H	3.969742	4.040411	0.508971	3.986368	4.018463	0.543880	1.277484	-2.379258	0.523162
atom	34			35			36		
	x	y	z	x	y	z	x	y	z
N	4.425023	-1.109412	2.408149	4.616536	1.350088	-1.859508	4.603205	1.351968	-1.892482
C	3.175649	-1.327235	1.700334	3.235296	1.415510	-1.363666	3.219811	1.413858	-1.402895
C	3.248784	-2.493099	0.695162	3.226265	2.473745	-0.264581	3.196016	2.486043	-0.317531
O	4.277210	-3.019671	0.370357	2.959329	3.632573	-0.456713	2.911170	3.638121	-0.524570
H	4.703160	-1.955744	2.890692	4.705542	0.618215	-2.553683	4.701104	0.610518	-2.575184
H	2.411140	-1.585216	2.438969	2.526111	1.783280	-2.112816	2.510843	1.766134	-2.159720
C	2.742618	-0.011856	1.006923	2.779748	0.028493	-0.890800	2.772603	0.029866	-0.913307
C	1.328755	-0.000267	0.480366	1.345494	0.000235	-0.424196	1.341132	0.000409	-0.437970
H	3.445465	0.203292	0.199279	3.452205	-0.311256	-0.104843	3.450117	-0.298004	-0.126647
H	2.870297	0.772165	1.754118	2.901750	-0.655609	-1.735479	2.894219	-0.662776	-1.751122
C	-1.313863	-0.000017	-0.476046	-1.331444	0.000715	0.420994	-1.327375	0.000905	0.433840
C	1.054038	-0.000655	-0.889301	0.295189	-0.000524	-1.345357	1.025957	0.000539	0.921412
C	0.243425	0.001051	1.359811	1.021818	0.000526	0.930368	0.286642	-0.000704	-1.350148
C	-1.065374	-0.000904	0.894942	-0.301505	-0.000997	1.356274	-1.036321	0.000044	-0.927770
C	-0.246981	0.000792	-1.369772	-1.029552	0.000047	-0.938083	-0.290035	-0.001192	1.360636
H	1.874360	-0.009394	-1.595132	0.516761	-0.006686	-2.405666	1.826163	0.007378	1.648804
H	0.420756	0.013409	2.428144	1.814821	0.007203	1.665533	0.497531	-0.006946	-2.412556
H	-1.891388	0.004707	1.595862	-0.531487	-0.004161	2.415228	-1.840248	-0.003592	-1.654251
H	-0.451261	-0.000940	-2.430879	-1.837834	-0.003454	-1.655558	-0.528580	-0.004130	2.414842
O	-2.574401	0.000830	-1.000367	-2.653806	-0.001431	0.772760	-2.607380	-0.000888	0.917657
H	-3.228720	0.001708	-0.294855	-2.738625	0.002287	1.731040	-3.234513	0.008450	0.188217
H	5.166019	-0.927353	1.739472	4.880818	2.222536	-2.300963	4.863060	2.219846	-2.345367
O	2.072082	-2.927512	0.187349	3.598355	2.000663	0.943895	3.574739	2.033049	0.895929
H	1.346997	-2.339748	0.442392	3.611088	2.753766	1.552490	3.572019	2.791784	1.497647

atom	37			38		
	x	y	z	x	y	z
N	2.784866	-1.964513	-2.140375	2.782696	-1.956935	-2.149358
C	3.465212	-1.393177	-0.977738	3.467667	-1.386496	-0.988038
C	3.554763	-2.367720	0.207467	3.567049	-2.370734	0.188132
O	4.592771	-2.828437	0.597864	4.606176	-2.852056	0.549827
H	3.248040	-2.809881	-2.449483	3.254105	-2.794303	-2.467966
H	4.502487	-1.222017	-1.260698	4.502533	-1.210720	-1.276738
C	2.862725	-0.018356	-0.603286	2.862435	-0.015299	-0.605807
C	1.385009	-0.005600	-0.288153	1.384431	-0.005397	-0.291370
H	3.054158	0.636516	-1.454089	3.053565	0.644740	-1.452670
H	3.425312	0.378714	0.243099	3.423662	0.378379	0.243080
C	-1.368390	-0.003510	0.286125	-1.368029	-0.003246	0.288324
C	0.428894	0.003668	-1.311739	0.918634	0.003169	1.028036
C	0.921440	0.003109	1.029029	0.431068	0.003429	-1.313750
C	-0.437929	0.001460	1.320856	-0.928777	0.000882	-1.033997
C	-0.929025	0.000874	-1.036117	-0.437327	0.001164	1.322757
H	0.759744	0.020510	-2.341503	1.630895	0.011352	1.843199
H	1.632189	0.009540	1.845377	0.760061	0.019696	-2.344015
H	-0.771017	0.008645	2.351560	-1.648882	0.011215	-1.843470
H	-1.661122	0.010870	-1.831135	-0.787130	0.008707	2.345145
O	-2.716489	-0.004896	0.499692	-2.689404	-0.003227	0.630336
H	-2.904084	-0.001834	1.443569	-3.238061	-0.000519	-0.160205
H	1.820305	-2.199187	-1.941658	1.824507	-2.207582	-1.939213
O	2.387004	-2.750335	0.782433	2.406812	-2.740894	0.785164
H	1.649139	-2.207913	0.468207	1.668767	-2.186665	0.492239

Table S21. Tables with the optimized Cartesian coordinates Trp conformers calculated at the B3LYP/D3/aug-cc-pVTZ level.

atom	1			2			3		
	x	y	z	x	y	z	x	y	z
H	-3.446180	0.017809	-2.740228	2.469823	-0.037044	3.642977	1.918074	-0.070123	-3.960423
C	-2.655709	0.000693	-2.009726	1.937842	-0.013250	2.706539	1.528352	-0.028118	-2.956823
C	-2.748020	0.000938	-0.643433	2.430657	-0.007970	1.429728	2.204686	-0.015718	-1.765080
C	-1.399131	0.001753	-0.144203	1.289721	0.001629	0.553107	1.201298	0.001930	-0.729501
N	-1.331322	-0.003020	-2.389331	0.558181	-0.007690	2.679229	0.168728	-0.015543	-2.730275
C	-0.534472	-0.000716	-1.268831	0.132667	-0.000859	1.369828	-0.061366	-0.001721	-1.372735
C	-0.836838	-0.001292	1.139849	1.139410	-0.000965	-0.839481	1.247230	-0.000586	0.671817
H	1.497830	-0.002316	-2.008249	-2.028700	-0.004235	1.471631	-2.215743	-0.001985	-1.173533
C	0.540687	-0.000248	1.271344	-0.136620	-0.000501	-1.374119	0.057586	-0.001001	1.377079
H	-1.470635	-0.010201	2.017178	2.006034	-0.011967	-1.486901	2.194773	0.006956	1.190226
H	0.985292	-0.004008	2.257177	-0.267798	-0.004879	-2.447421	0.080396	-0.000043	2.458154
C	1.377332	0.001328	0.141741	-1.271357	0.001308	-0.544525	-1.183640	0.001253	0.717575
H	2.450226	0.002193	0.276398	-2.255381	0.001010	-0.992951	-2.094708	0.003056	1.300407
C	0.852421	-0.000825	-1.139900	-1.153821	-0.000612	0.835190	-1.261109	0.000124	-0.664235
C	-4.006693	0.060386	0.165461	3.865597	0.024885	1.020617	3.693192	-0.004855	-1.610270
H	-3.839226	-0.383161	1.148625	4.075971	-0.743771	0.276310	4.150469	-0.384880	-2.525523
H	-4.798237	-0.509658	-0.318476	4.505375	-0.176562	1.882895	4.001044	-0.660854	-0.796099
C	-4.519319	1.500785	0.364167	4.278816	1.383250	0.406870	4.293302	1.396385	-1.331452
H	-5.415079	1.468246	0.990006	3.681196	1.551356	-0.491015	5.376053	1.318911	-1.450146
C	-5.001462	2.087833	-0.978511	5.737923	1.268474	-0.049744	4.112320	1.805954	0.143366
O	-4.715466	3.384175	-1.140020	6.617000	1.882034	0.757382	3.696697	3.063122	0.318029
H	-4.198765	3.633179	-0.341222	6.073673	2.323857	1.445124	3.585018	3.422077	-0.590964
O	-5.605036	1.446065	-1.798440	6.067956	0.646721	-1.023855	4.361790	1.064749	1.058601
N	-3.570046	2.430018	0.991189	4.139527	2.459141	1.394261	3.804514	2.462378	-2.212974
H	-3.638422	2.417744	1.998726	3.948052	3.347886	0.949919	4.426546	2.635163	-2.989180
H	-2.615151	2.197442	0.741563	3.370482	2.265191	2.024970	2.889179	2.228462	-2.582727
H	-1.003444	-0.001768	-3.337337	-0.037801	-0.041758	3.485087	-0.538823	-0.053693	-3.439900
atom	4			5			6		
	x	y	z	x	y	z	x	y	z
H	2.342248	-0.033822	-3.725966	-2.328927	-0.033304	-3.733624	2.274171	-0.044656	-3.767626
C	1.844972	-0.015725	-2.770739	-1.834296	-0.013405	-2.776885	1.792002	-0.024179	-2.805022
C	2.382923	-0.004130	-1.512696	-2.375592	-0.006224	-1.521446	2.353677	-0.010164	-1.556699
C	1.272732	0.002395	-0.597263	-1.268507	0.002126	-0.602464	1.260907	0.002492	-0.620570
N	0.468141	-0.009580	-2.694843	-0.455526	-0.008686	-2.698142	0.416424	-0.015890	-2.703028
C	0.086400	-0.001175	-1.373728	-0.080189	-0.000861	-1.374198	0.060973	-0.002078	-1.374159
C	1.166209	-0.001670	0.800918	-1.170234	-0.001537	0.795134	1.181066	-0.000786	0.777896
H	-2.076432	-0.004132	-1.402481	2.083262	-0.005690	-1.392791	-2.102323	-0.001260	-1.363523
C	-0.091121	-0.000327	1.378123	0.084372	-0.000365	1.378683	-0.064818	-0.001383	1.378838
H	2.053398	-0.010927	1.420508	-2.060252	-0.018317	1.410209	2.082137	-0.000451	1.375307
H	-0.185757	-0.005018	2.455288	0.174260	-0.006022	2.456255	-0.139666	-0.004608	2.457823
C	-1.253316	0.001605	0.587363	1.249923	0.001682	0.593094	-1.242335	0.001852	0.610835
H	-2.221758	0.001554	1.068327	2.216173	0.001276	1.078634	-2.201635	0.004027	1.110084
C	-1.180905	-0.000829	-0.795413	1.184634	-0.001046	-0.790250	-1.195793	-0.000097	-0.772840
C	3.836485	0.035240	-1.162116	-3.823192	0.038163	-1.162050	3.814742	0.019782	-1.234149
H	4.424964	-0.514343	-1.895579	-4.075661	-0.740783	-0.442186	4.377914	-0.523784	-1.992395
H	3.998152	-0.449560	-0.196216	-4.438127	-0.146868	-2.047045	3.994542	-0.469574	-0.277279
C	4.415516	1.466669	-1.096103	-4.235342	1.393266	-0.553346	4.419914	1.446312	-1.182853
H	4.303899	1.920645	-2.083488	-3.634135	1.567289	0.341332	5.477571	1.334494	-0.931001
C	5.923686	1.349927	-0.833579	-5.688842	1.344507	-0.049378	3.798116	2.266058	-0.053422
O	6.285079	1.627196	0.428317	-6.388715	2.461965	-0.289970	4.074721	1.734703	1.164612
H	5.459655	1.908536	0.879247	-5.773931	3.051682	-0.778517	3.640637	2.293568	1.825913
O	6.706117	0.998515	-1.675524	-6.149594	0.399648	0.533768	3.151506	3.270515	-0.191951
N	3.782767	2.251236	-0.029279	-4.047899	2.551177	-1.437639	4.317846	2.087905	-2.480137
H	3.816917	3.242526	-0.230267	-3.117977	2.935367	-1.343792	3.339982	2.232498	-2.706916
H	2.809698	2.001953	0.092440	-4.156402	2.283339	-2.409190	4.738119	3.008732	-2.452876
H	-0.154768	-0.042414	-3.480336	0.170498	-0.046783	-3.480604	-0.221924	-0.035754	-3.476164

atom	7			8			9		
	x	y	z	x	Y	z	x	y	z
H	-3.330619	-0.031076	-2.875779	-2.254245	0.046883	-3.779137	2.094700	-0.027036	-3.870806
C	-2.573840	-0.016777	-2.109940	-1.780031	0.022866	-2.812306	1.658114	-0.012563	-2.886519
C	-2.720899	-0.009057	-0.749495	-2.348094	0.004772	-1.567608	2.278333	-0.006188	-1.666513
C	-1.391110	0.000763	-0.198286	-1.259867	-0.004619	-0.627032	1.230590	0.001259	-0.678504
N	-1.235228	-0.009638	-2.440404	-0.404458	0.015978	-2.704587	0.288652	-0.006618	-2.720203
C	-0.484490	-0.000974	-1.287760	-0.055150	0.002903	-1.374730	-0.003295	-0.000915	-1.375417
C	-0.881506	0.000080	1.107406	-1.183168	0.002501	0.772326	1.217088	-0.000535	0.722804
H	1.575238	0.001101	-1.949426	2.107842	0.005724	-1.353786	-2.163951	-0.001799	-1.264726
C	0.489780	-0.000723	1.290512	0.059447	0.001439	1.380012	-0.000576	-0.000555	1.379882
H	-1.553354	0.013825	1.954465	-2.082393	0.027016	1.374180	2.143263	0.007924	1.280545
H	0.895594	0.000301	2.292962	0.128760	0.010299	2.459115	-0.024029	-0.002813	2.461166
C	1.370431	0.000528	0.194604	1.239993	-0.003263	0.616616	-1.212796	0.000927	0.667855
H	2.437373	0.002298	0.371273	2.197007	-0.004156	1.119905	-2.147856	0.001002	1.211316
C	0.896895	0.000326	-1.106475	1.198745	0.001038	-0.767192	-1.231011	-0.000180	-0.716621
C	-4.015128	0.011036	0.002836	-3.809539	-0.051161	-1.255019	3.754278	0.018002	-1.421495
H	-3.921607	-0.565143	0.923977	-4.393095	0.468548	-2.013288	4.278519	-0.434211	-2.263067
H	-4.798623	-0.451312	-0.595495	-4.012019	0.459720	-0.309532	3.998194	-0.559159	-0.529417
C	-4.485141	1.428175	0.409614	-4.340908	-1.493398	-1.155139	4.343753	1.443102	-1.250484
H	-5.465963	1.318780	0.882149	-4.159394	-1.987344	-2.113218	5.419639	1.325233	-1.107908
C	-4.715805	2.295500	-0.828959	-5.871909	-1.510695	-0.989935	3.840577	2.029707	0.068058
O	-5.620940	1.738558	-1.675439	-6.323763	-2.498984	-0.204326	2.975055	3.054421	-0.092530
H	-5.734791	2.352723	-2.415476	-5.521460	-2.961771	0.120717	2.670779	3.311543	0.790423
O	-4.195067	3.357041	-1.048951	-6.603603	-0.732725	-1.541340	4.164513	1.613843	1.152985
N	-3.578937	2.026523	1.371191	-3.721305	-2.329794	-0.118814	4.118928	2.229232	-2.450584
H	-2.657255	2.131570	0.962160	-2.835448	-2.702043	-0.432899	3.125036	2.334016	-2.616789
H	-3.886560	2.960749	1.611797	-3.524475	-1.786186	0.713274	4.503645	3.159975	-2.354967
H	-0.868570	-0.017369	-3.373709	0.236993	0.054116	-3.474643	-0.385459	-0.032800	-3.462142
atom	10			11			12		
	x	y	z	x	y	z	x	y	z
H	-3.325789	-0.016837	-2.883541	2.425621	-0.016668	3.672246	3.379662	-0.000651	-2.818851
C	-2.568469	-0.012507	-2.118182	1.905176	-0.004373	2.729424	2.608515	-0.005332	-2.067161
C	-2.718747	-0.009114	-0.757771	2.413141	-0.006902	1.459157	2.729795	-0.006762	-0.705629
C	-1.390730	0.000108	-0.202531	1.282652	0.000093	0.569472	1.392711	-0.000011	-0.177405
N	-1.228420	-0.004500	-2.444247	0.525389	-0.001615	2.685609	1.274525	-0.000933	-2.421293
C	-0.480536	-0.000340	-1.289526	0.115293	0.000130	1.371606	0.504090	0.000329	-1.280792
C	-0.884898	0.000222	1.104579	1.149758	-0.000103	-0.824501	0.864651	-0.000141	1.119191
H	1.581311	0.000048	-1.944471	-2.047110	-0.001393	1.445553	-1.545983	-0.001801	-1.972097
C	0.485981	-0.000320	1.292158	-0.119230	-0.000115	-1.376011	-0.509300	-0.000031	1.283952
H	-1.559414	0.012954	1.949645	2.025356	-0.002315	-1.460139	1.521299	-0.003007	1.979375
H	0.888685	0.000422	2.295892	-0.236891	-0.000312	-2.450975	-0.930330	-0.000688	2.280105
C	1.369988	0.000089	0.199012	-1.264396	0.000341	-0.560931	-1.372638	0.000350	0.174731
H	2.436408	0.000509	0.378878	-2.242741	0.000907	-1.021706	-2.442119	0.000593	0.335296
C	0.900195	0.000240	-1.103691	-1.164077	-0.000345	0.820366	-0.879513	-0.000496	-1.119677
C	-4.017045	0.009113	-0.011606	3.848869	0.032852	1.060460	3.996694	0.035764	0.084279
H	-3.924464	-0.550459	0.919458	4.038382	-0.694358	0.267222	4.842635	-0.246362	-0.545544
H	-4.794243	-0.463872	-0.609284	4.487418	-0.228566	1.906656	3.962163	-0.669640	0.916099
C	-4.499048	1.429966	0.358427	4.271789	1.449110	0.551579	4.296243	1.422447	0.687935
H	-5.474588	1.325315	0.843089	3.656447	1.686191	-0.317319	3.474710	1.717391	1.338861
C	-4.797235	2.196171	-0.935569	5.711303	1.355478	0.096767	4.399310	2.434081	-0.450634
O	-4.231932	3.424638	-0.976584	5.811168	0.833872	-1.148557	3.409626	3.352699	-0.430157
H	-4.488874	3.824890	-1.820409	6.754424	0.744851	-1.349846	3.540533	3.940120	-1.190067
O	-5.481413	1.770534	-1.832794	6.671904	1.644018	0.763497	5.273990	2.431895	-1.281560
N	-3.594058	2.060474	1.303981	4.148383	2.517129	1.526198	5.520738	1.359923	1.480508
H	-2.666362	2.148707	0.906874	3.270452	2.438549	2.023951	6.319003	1.210748	0.872917
H	-3.904080	2.994333	1.537883	4.907747	2.473196	2.195672	5.680103	2.227515	1.977693
H	-0.859559	-0.023267	-3.376587	-0.080145	-0.019500	3.484737	0.924422	-0.017054	-3.360759

atom	13			14			15		
	x	y	z	x	y	z	x	y	z
H	-1.802926	-0.017961	-4.013611	-2.486248	-0.001099	-3.631547	2.425529	-0.032873	-3.671797
C	-1.442547	-0.006675	-2.998599	-1.948981	-0.004360	-2.698526	1.907288	-0.016094	-2.727733
C	-2.149479	-0.001856	-1.828151	-2.436321	-0.005869	-1.421479	2.417006	-0.006835	-1.457586
C	-1.177307	0.000385	-0.767255	-1.292309	0.000360	-0.549488	1.285977	0.001861	-0.568078
N	-0.088768	-0.001685	-2.733629	-0.568146	-0.001201	-2.676708	0.528781	-0.009733	-2.683404
C	0.104438	-0.000100	-1.371560	-0.136792	0.000301	-1.369907	0.117776	-0.001461	-1.370951
C	-1.266701	-0.000302	0.631073	-1.136441	-0.000575	0.842288	1.147591	-0.000681	0.826631
H	2.250997	-0.001037	-1.101987	2.023947	-0.003181	-1.477469	-2.044229	-0.000958	-1.449329
C	-0.100969	-0.000074	1.376451	0.141248	0.000114	1.374176	-0.122008	-0.000935	1.375500
H	-2.231078	0.012231	1.119895	-2.002050	-0.006664	1.491621	2.021579	-0.002254	1.463924
H	-0.157259	-0.000391	2.456511	0.275684	-0.001698	2.447245	-0.241025	-0.003021	2.450315
C	1.160348	0.000368	0.755398	1.273485	0.000558	0.541361	-1.266513	0.001376	0.558714
H	2.052901	0.000267	1.366157	2.259066	0.000364	0.986488	-2.245592	0.003143	1.017812
C	1.280191	-0.000276	-0.624107	1.150810	-0.000758	-0.838431	-1.162823	-0.000160	-0.821816
C	-3.638508	0.022029	-1.693255	-3.868344	0.035988	-0.999504	3.860976	0.028813	-1.075843
H	-3.944533	-0.559498	-0.819634	-4.037733	-0.664452	-0.181785	4.460871	-0.486296	-1.828423
H	-4.110032	-0.439095	-2.560962	-4.515761	-0.253165	-1.828057	4.010568	-0.493682	-0.128056
C	-4.265300	1.431558	-1.563715	-4.285860	1.453187	-0.539902	4.406952	1.484429	-0.920693
H	-3.980791	2.018903	-2.435900	-3.653608	1.713549	0.323307	4.325089	1.967473	-1.895729
C	-3.710133	2.115286	-0.318568	-5.700503	1.488806	0.005292	5.876221	1.377669	-0.569327
O	-2.724234	2.991618	-0.598793	-5.911896	0.516924	0.926044	6.643269	1.169403	-1.665648
H	-2.371072	3.316448	0.243476	-6.815780	0.628276	1.254497	7.554828	1.048964	-1.360893
O	-4.091785	1.890026	0.803595	-6.543889	2.296620	-0.287545	6.327072	1.407095	0.546813
N	-5.717952	1.307507	-1.530532	-4.173952	2.391530	-1.643867	3.745189	2.309906	0.072826
H	-6.004030	0.880376	-0.655948	-4.666803	3.252464	-1.448169	2.740131	2.224192	0.002359
H	-6.158595	2.218676	-1.566893	-3.204484	2.594034	-1.842805	4.029234	2.038142	1.006220
H	0.637411	-0.022717	-3.424756	0.024133	-0.023074	-3.485566	-0.076562	-0.030896	-3.482733

atom	16			17			18		
	x	y	z	x	y	z	x	y	z
H	2.759637	-0.024137	-3.426713	-2.339646	-0.018995	-3.727433	2.316634	-0.048901	-3.740853
C	2.152615	-0.003395	-2.538043	-1.840434	-0.009606	-2.773284	1.825720	-0.022783	-2.782399
C	2.537440	0.003845	-1.227996	-2.379192	-0.006651	-1.516491	2.373566	-0.012913	-1.528591
C	1.330464	0.000981	-0.447757	-1.269886	0.001191	-0.600159	1.268951	0.000395	-0.604672
N	0.775070	-0.002742	-2.624273	-0.461301	-0.005558	-2.696896	0.448665	-0.009304	-2.698147
C	0.242281	-0.001385	-1.355405	-0.082771	-0.000520	-1.374058	0.077886	-0.000972	-1.373369
C	1.068185	-0.000021	0.927694	-1.168555	-0.000766	0.796716	1.172158	0.000509	0.793818
H	-1.903467	0.001752	-1.629711	2.080637	-0.003522	-1.396483	-2.085721	0.000568	-1.390410
C	-0.247153	-0.000538	1.359189	0.086929	-0.000358	1.378648	-0.082070	-0.000838	1.377528
H	1.881424	0.000155	1.641946	-2.058727	-0.009002	1.411771	2.067433	0.018837	1.398844
H	-0.464210	-0.001166	2.418721	0.178836	-0.003651	2.456172	-0.170005	-0.000114	2.455469
C	-1.311535	0.000150	0.441392	1.251219	0.001057	0.591103	-1.250164	0.000266	0.594851
H	-2.328679	0.000088	0.808842	2.218448	0.000986	1.074830	-2.215697	0.000969	1.082144
C	-1.082242	0.000815	-0.925113	1.183064	-0.000605	-0.792250	-1.186761	0.000639	-0.788156
C	3.924185	-0.040995	-0.683882	-3.829442	0.024661	-1.156791	3.836004	-0.010681	-1.208825
H	4.005900	0.633267	0.170814	-4.045684	-0.709197	-0.380050	4.391465	-0.437090	-2.042500
H	4.645712	0.297989	-1.430097	-4.441876	-0.234891	-2.020601	4.029458	-0.630345	-0.330880
C	4.339780	-1.469333	-0.234408	-4.270704	1.406439	-0.652809	4.388147	1.404031	-0.920005
H	3.532803	-1.874400	0.376653	-3.670031	1.653058	0.237574	4.214645	2.005145	-1.827486
C	5.575799	-1.349734	0.648655	-5.704790	1.367149	-0.148910	5.897894	1.413988	-0.750398
O	5.262783	-0.933546	1.899951	-6.195861	2.615456	0.050919	6.526900	0.727434	-1.735150
H	6.091701	-0.834443	2.391404	-7.098611	2.519564	0.387675	7.478338	0.818616	-1.580655
O	6.708619	-1.557664	0.295186	-6.339791	0.376221	0.100330	6.498540	1.996531	0.115171
N	4.538410	-2.327937	-1.387676	-4.137241	2.402774	-1.715849	3.778176	1.965170	0.273358
H	5.432151	-2.119883	-1.819515	-4.384496	3.326378	-1.386292	4.290799	2.771228	0.604871
H	4.569333	-3.301472	-1.113456	-3.175946	2.427041	-2.033196	2.816092	2.222847	0.103825
H	0.248153	-0.002278	-3.477295	0.162836	-0.031486	-3.481293	-0.180421	-0.037388	-3.478547

atom	19			20			21		
	x	y	z	x	y	z	x	y	z
H	2.466792	-0.016030	3.644671	-1.807978	-0.010222	-4.011538	3.316299	-0.004640	-2.892256
C	1.936047	-0.004523	2.707600	-1.446983	-0.002677	-2.996674	2.562613	-0.005542	-2.123216
C	2.429549	-0.007405	1.431878	-2.151962	-0.001096	-1.824725	2.713867	-0.005643	-0.764690
C	1.288882	0.000059	0.554898	-1.177916	0.000024	-0.765454	1.388342	0.000529	-0.207582
N	0.555757	-0.001377	2.679599	-0.092773	0.000735	-2.733660	1.221484	-0.004376	-2.448145
C	0.130720	0.000196	1.370216	0.102538	0.000190	-1.371793	0.476192	-0.000221	-1.291221
C	1.140568	-0.000129	-0.837692	-1.265938	-0.000155	0.632832	0.888673	-0.000259	1.100263
H	-2.030821	-0.001734	1.468691	2.249541	-0.000377	-1.104987	-1.588253	-0.000376	-1.938170
C	-0.134679	-0.000061	-1.374476	-0.099227	0.000070	1.376637	-0.481233	-0.000332	1.294583
H	2.008722	-0.002605	-1.483425	-2.229821	0.013244	1.122839	1.563481	-0.002013	1.946232
H	-0.264294	-0.000384	-2.448036	-0.153948	0.000402	2.456756	-0.880567	-0.000169	2.299593
C	-1.270612	0.000319	-0.546517	1.161195	0.000145	0.753699	-1.368374	0.000652	0.204304
H	-2.254084	0.000727	-0.996287	2.054551	0.000173	1.363257	-2.434093	0.002339	0.388063
C	-1.154879	-0.000383	0.833571	1.279347	-0.000273	-0.625921	-0.903601	-0.000369	-1.100346
C	3.860139	0.033246	1.014787	-3.639038	0.035721	-1.678858	3.995412	0.033925	0.002355
H	4.042158	-0.697200	0.222244	-3.949765	-0.535390	-0.800920	4.828414	-0.259906	-0.638172
H	4.510373	-0.224420	1.853307	-4.109873	-0.432938	-2.545878	3.968320	-0.669162	0.836879
C	4.269800	1.445684	0.490797	-4.259961	1.460051	-1.557090	4.296617	1.418880	0.599197
H	3.629521	1.687700	-0.356236	-3.948635	2.045683	-2.421737	3.477532	1.716952	1.251668
C	5.671239	1.338744	-0.072079	-3.719296	2.126277	-0.305691	4.365047	2.491637	-0.482734
O	6.624966	1.534270	0.871676	-2.710238	2.978135	-0.562398	5.278643	2.185443	-1.444244
H	7.483930	1.412272	0.441854	-2.362833	3.285394	0.288700	5.299419	2.929226	-2.063321
O	5.934287	1.042807	-1.208858	-4.117551	1.887548	0.807504	3.742878	3.521577	-0.491866
N	4.165232	2.523620	1.464107	-5.715003	1.472162	-1.468426	5.521366	1.365371	1.398619
H	3.227065	2.545285	1.845716	-6.125700	0.899305	-2.194488	6.325340	1.169988	0.813797
H	4.807805	2.379190	2.233756	-6.012136	1.107313	-0.570627	5.690767	2.243461	1.873225
H	-0.040694	-0.022613	3.485436	0.632449	-0.014469	-3.425968	0.850546	-0.006870	-3.379661

atom	22			23			24		
	x	y	z	x	y	z	x	y	z
H	-3.360606	-0.001186	2.841686	-2.232742	0.048705	-3.791225	3.382482	-0.047896	2.815624
C	-2.594779	-0.005172	2.084535	-1.763571	0.022075	-2.821999	2.609409	-0.005530	2.069396
C	-2.725348	-0.007216	0.723603	-2.339151	0.010501	-1.580943	2.731147	0.000881	0.707727
C	-1.391926	-0.000427	0.186288	-1.254763	-0.001127	-0.633583	1.395045	-0.000337	0.176963
N	-1.258565	0.000246	2.429405	-0.388270	0.010240	-2.707355	1.276211	-0.000548	2.419204
C	-0.495984	0.000444	1.283828	-0.046495	0.001062	-1.375215	0.504554	-0.000452	1.280558
C	-0.871470	0.000238	-1.113357	-1.189675	0.000289	0.766873	0.863647	0.000694	-1.118833
H	1.558269	-0.000481	1.962102	2.116737	0.001274	-1.342173	-1.544126	0.002296	1.972569
C	0.501268	-0.000064	-1.287164	0.050773	0.000631	1.379493	-0.510836	-0.000246	-1.283584
H	-1.532478	0.001008	-1.970240	-2.097542	-0.009234	1.353956	1.516890	0.004997	-1.982183
H	0.915835	0.000816	-2.285978	0.114111	0.001688	2.459111	-0.931938	0.000635	-2.279815
C	1.371578	0.000074	-0.183467	1.236066	-0.000719	0.623344	-1.373869	-0.000554	-0.174541
H	2.439970	0.000810	-0.350807	2.190257	-0.001028	1.132376	-2.443409	-0.000944	-0.334261
C	0.886535	-0.000265	1.113872	1.204094	-0.000136	-0.760912	-0.878541	0.000896	1.119437
C	-3.999187	0.042366	-0.053603	-3.806423	0.000819	-1.283772	3.989858	-0.038915	-0.100958
H	-4.844117	-0.227966	0.582037	-4.356802	0.466221	-2.100151	4.776114	0.543731	0.381239
H	-3.969670	-0.676729	-0.876971	-4.015134	0.587545	-0.387675	3.798171	0.424085	-1.071120
C	-4.302647	1.431589	-0.676230	-4.372116	-1.410902	-1.072087	4.517004	-1.465997	-0.369598
H	-3.473418	1.725951	-1.317787	-4.177361	-1.990235	-1.990065	3.660009	-2.141143	-0.438210
C	-4.421766	2.443934	0.450082	-5.889763	-1.371229	-0.959594	5.351730	-2.000472	0.809871
O	-3.385838	3.302600	0.499292	-6.387973	-2.558505	-0.532665	6.221609	-2.962171	0.461954
H	-3.527550	3.886220	1.260042	-7.351802	-2.471713	-0.500742	6.198216	-3.002357	-0.516099
O	-5.335524	2.465812	1.236716	-6.597150	-0.442714	-1.250329	5.236296	-1.622239	1.944533
N	-5.540068	1.494587	-1.448761	-3.801940	-2.035589	0.121814	5.293920	-1.645641	-1.606016
H	-5.548676	0.782475	-2.168035	-4.171784	-2.967017	0.258074	4.690876	-1.674810	-2.416974
H	-6.336643	1.337489	-0.841592	-2.796755	-2.108640	0.028472	5.948600	-0.883258	-1.740194
H	-0.901911	-0.016048	3.366427	0.257616	0.041011	-3.473813	0.927346	-0.016825	3.359324

atom	25			26			27		
	x	y	z	x	y	z	x	y	z
H	-1.943909	-0.027295	-3.946948	2.373571	-0.031794	-3.705662	-3.693549	0.033786	-2.393890
C	-1.549358	-0.016844	-2.944771	1.868406	-0.015542	-2.754526	-2.841026	-0.000522	-1.739918
C	-2.214135	-0.008100	-1.749227	2.396242	-0.006564	-1.491784	-2.795797	-0.001286	-0.372867
C	-1.204823	0.001492	-0.724551	1.278203	0.002158	-0.586226	-1.404148	0.000223	-0.008827
N	-0.187785	-0.011487	-2.727722	0.490729	-0.009802	-2.690556	-1.558107	-0.003753	-2.247184
C	0.054704	-0.001254	-1.374263	0.098551	-0.001730	-1.372390	-0.654722	-0.000076	-1.210850
C	-1.242347	-0.000375	0.676026	1.158831	-0.000757	0.810052	-0.723792	-0.000124	1.214642
H	2.209456	-0.000389	-1.182244	-2.064258	-0.001161	-1.420325	1.296310	0.000271	-2.143598
C	-0.051233	-0.001008	1.379305	-0.102721	-0.001115	1.377132	0.660783	-0.000129	1.213220
H	-2.189810	0.005459	1.196355	2.041377	-0.004515	1.435473	-1.270135	0.003707	2.148947
H	-0.067440	-0.003660	2.460824	-0.206573	-0.003947	2.453540	1.198285	0.001516	2.151709
C	1.187047	0.001277	0.713009	-1.258687	0.001591	0.576629	1.384106	0.000281	0.008723
H	2.100987	0.003001	1.291158	-2.231205	0.003389	1.049427	2.465126	0.001703	0.039039
C	1.256652	-0.000132	-0.669527	-1.174177	-0.000148	-0.805197	0.737773	-0.000176	-1.216907
C	-3.697934	0.034334	-1.554422	3.846056	0.032884	-1.133194	-3.911787	-0.011422	0.625088
H	-3.953546	-0.370591	-0.573241	4.436999	-0.472034	-1.900114	-3.927554	0.927853	1.187796
H	-4.208883	-0.583841	-2.293290	4.015311	-0.497970	-0.193428	-3.691849	-0.791093	1.363235
C	-4.301755	1.447027	-1.671923	4.383363	1.489285	-0.978881	-5.330044	-0.292545	0.097085
H	-4.105952	1.831042	-2.671998	4.262368	1.988774	-1.939421	-5.292136	-1.129964	-0.602843
C	-3.618229	2.425971	-0.722916	5.878643	1.404882	-0.745302	-5.876872	0.915684	-0.682835
O	-3.811392	2.083044	0.577705	6.186042	1.237237	0.564435	-6.544955	1.795650	0.078644
H	-3.342348	2.734025	1.119316	7.148837	1.156258	0.626747	-6.632316	1.377747	0.960035
O	-3.003297	3.407269	-1.049402	6.713700	1.411666	-1.612265	-5.715027	1.080900	-1.862244
N	-5.750421	1.383329	-1.480081	3.737075	2.302487	0.042268	-6.218230	-0.530220	1.246313
H	-5.974244	1.106392	-0.531565	2.737912	2.330102	-0.118056	-7.061281	-1.018956	0.971795
H	-6.182966	2.283765	-1.645142	3.887986	1.913564	0.964719	-5.761416	-1.079600	1.961975
H	0.513200	-0.017822	-3.444766	-0.125821	-0.031622	-3.481275	-1.327764	0.020953	-3.222797

atom	28			29			30		
	x	y	z	x	y	z	x	y	z
H	3.031116	0.023793	3.192059	-3.330699	0.026513	2.876395	3.067834	-0.000263	-3.157024
C	2.353338	0.013374	2.355174	-2.571207	0.000486	2.114740	2.377909	-0.002628	-2.332986
C	2.633645	0.000919	1.013065	-2.717801	-0.003880	0.755923	2.643207	-0.005446	-0.990174
C	1.362228	-0.003432	0.334645	-1.390767	0.000207	0.202711	1.368572	-0.000446	-0.322947
N	0.989841	0.016126	2.551584	-1.231184	-0.000215	2.442947	1.013054	-0.001651	-2.539114
C	0.354877	0.002655	1.329327	-0.480788	0.000326	1.289742	0.367050	0.000358	-1.325896
C	0.986941	0.001432	-1.014847	-0.884452	-0.000429	-1.102798	0.976796	0.000318	1.021829
H	-1.760555	0.002887	1.783896	1.580902	-0.002057	1.943646	-1.743355	-0.000642	-1.799346
C	-0.359186	0.001404	-1.333036	0.486628	0.000108	-1.292946	-0.372427	-0.000097	1.329700
H	1.736767	0.011418	-1.794598	-1.555151	-0.002410	-1.952409	1.716578	0.001976	1.811535
H	-0.664500	0.005751	-2.370288	0.889315	-0.000321	-2.296746	-0.685849	-0.000281	2.364817
C	-1.343024	-0.002260	-0.328912	1.370050	0.000432	-0.199898	-1.347965	-0.000001	0.317907
H	-2.387008	-0.004217	-0.610532	2.436477	0.000636	-0.379528	-2.394739	-0.000013	0.589684
C	-1.001836	0.000201	1.012822	0.899330	-0.000644	1.103190	-0.992026	-0.000131	-1.020593
C	3.979661	0.016744	0.360328	-3.997230	0.025005	-0.017911	3.972534	-0.000926	-0.298169
H	4.755942	0.233214	1.097932	-4.811221	-0.418314	0.553630	3.839272	-0.404042	0.707981
H	4.015818	0.817152	-0.380667	-3.881882	-0.572944	-0.925892	4.343580	1.020761	-0.164899
C	4.352929	-1.279372	-0.404758	-4.413135	1.437279	-0.443934	5.071865	-0.821438	-0.998214
H	3.477157	-1.590426	-0.981643	-3.527953	1.970032	-0.816169	4.677075	-1.811208	-1.223543
C	4.672856	-2.442721	0.553344	-4.932045	2.240299	0.742921	6.211116	-1.047835	-0.009089
O	3.688791	-2.799812	1.409231	-5.143647	3.532580	0.386712	6.975861	0.063435	0.150938
H	2.962946	-2.155560	1.379301	-5.508297	3.991167	1.157797	7.656761	-0.146499	0.806529
O	5.717084	-3.035551	0.547627	-5.154333	1.829669	1.850445	6.422842	-2.070805	0.589354
N	5.428744	-0.999497	-1.339779	-5.504348	1.381069	-1.427698	5.480539	-0.190507	-2.251739
H	6.252537	-0.695363	-0.831572	-5.748801	2.310019	-1.747325	5.904042	0.712228	-2.072099
H	5.707084	-1.853609	-1.808539	-5.215116	0.845054	-2.236398	6.167412	-0.754931	-2.736193
H	0.534456	0.008886	3.445407	-0.864823	0.001173	3.376369	0.566096	-0.006406	-3.436657

atom	31			32			33		
	x	y	z	x	y	z	x	y	z
H	-3.349346	-0.002504	2.853856	-3.764176	0.023000	-2.279651	-3.054039	0.000115	-3.166211
C	-2.587230	-0.004447	2.092996	-2.894893	0.000644	-1.645986	-2.370440	0.010444	-2.334748
C	-2.722662	-0.005389	0.732623	-2.806334	0.001009	-0.280325	-2.639612	0.003553	-0.996453
C	-1.391028	0.000089	0.190952	-1.403231	0.000586	0.036159	-1.366934	-0.001253	-0.327025
N	-1.250331	-0.003609	2.433311	-1.628833	-0.002259	-2.196888	-1.006076	0.008177	-2.543958
C	-0.491726	-0.000005	1.285350	-0.693149	-0.000398	-1.189364	-0.363111	0.000346	-1.327622
C	-0.875126	0.000074	-1.110434	-0.684667	-0.000330	1.237400	-0.979690	0.000981	1.018840
H	1.564649	0.000880	1.956897	1.227741	-0.000124	-2.184079	1.748983	0.003473	-1.793659
C	0.496911	-0.000325	-1.288811	0.698979	-0.000123	1.191664	0.368695	0.000230	1.331553
H	-1.538753	0.001421	-1.965329	-1.202815	0.000180	2.187477	-1.723145	0.007963	1.805410
H	0.908218	0.001270	-2.288962	1.266506	0.000118	2.112309	0.679551	0.002248	2.367418
C	1.370784	0.000411	-0.187948	1.383336	0.000324	-0.035665	1.346416	-0.001168	0.322154
H	2.438609	0.002686	-0.358848	2.464823	0.000905	-0.039851	2.392399	-0.001925	0.596730
C	0.890185	-0.000243	1.110890	0.698731	-0.000059	-1.240195	0.994624	0.000864	-1.017900
C	-3.995927	0.047454	-0.045128	-3.907679	-0.016183	0.734621	-3.977340	-0.003897	-0.333687
H	-4.840228	-0.237387	0.584716	-3.793101	0.827015	1.422462	-4.769640	0.222407	-1.047310
H	-3.961883	-0.663034	-0.875889	-3.819588	-0.918607	1.348949	-4.010644	0.790213	0.415922
C	-4.295657	1.440015	-0.658460	-5.345554	0.003207	0.182726	-4.332694	-1.310327	0.424428
H	-3.461118	1.740707	-1.289247	-5.468210	-0.802935	-0.539237	-3.393396	-1.731468	0.812991
C	-4.373139	2.491583	0.435876	-5.633985	1.284968	-0.585215	-4.889228	-2.408569	-0.466312
O	-5.447588	2.282486	1.243506	-5.639617	2.362134	0.234906	-4.233367	-2.517117	-1.642502
H	-5.453765	2.993325	1.900462	-5.839502	3.139952	-0.305764	-4.627512	-3.265790	-2.114010
O	-3.600752	3.394544	0.607053	-5.830774	1.366696	-1.769571	-5.795011	-3.144179	-0.163466
N	-5.520797	1.507176	-1.454376	-6.386465	-0.146270	1.202194	-5.312857	-1.029123	1.466339
H	-5.450720	0.905078	-2.264934	-6.313114	-1.050251	1.651379	-5.790042	-1.874040	1.753127
H	-6.320247	1.205103	-0.910439	-6.293039	0.565164	1.917259	-4.872990	-0.610584	2.274214
H	-0.890270	0.002345	3.369092	-1.429409	0.016842	-3.179368	-0.556740	0.019824	-3.440200

atom	34			35			36		
	x	y	z	x	y	z	x	y	z
H	3.073677	0.082185	-3.149189	-2.721476	-0.002205	-3.459397	-1.896573	-0.056791	-3.969342
C	2.386183	0.038942	-2.320996	-2.128065	0.010050	-2.560525	-1.514802	-0.028016	-2.962245
C	2.652551	0.010348	-0.974091	-2.531023	0.005377	-1.248310	-2.195508	-0.018084	-1.774566
C	1.370026	-0.004709	-0.312204	-1.327187	-0.000970	-0.457432	-1.197752	-0.000393	-0.736752
N	1.026524	0.030245	-2.535114	-0.752460	0.006611	-2.629719	-0.155702	-0.010625	-2.729807
C	0.376604	0.004837	-1.321996	-0.232158	-0.000114	-1.355290	0.069005	-0.000838	-1.372413
C	0.970025	0.000955	1.031554	-1.074931	0.001167	0.920540	-1.250368	0.001318	0.663620
H	-1.730345	0.000345	-1.813697	1.916121	0.003573	-1.616182	2.222452	0.001720	-1.160327
C	-0.381336	0.002756	1.325505	0.235528	-0.000255	1.359514	-0.065819	-0.000997	1.377081
H	1.710126	-0.009264	1.818815	-1.891161	0.011643	1.630013	-2.202925	0.027905	1.170931
H	-0.703214	0.006913	2.357821	0.444657	0.003616	2.420289	-0.094214	0.002928	2.458279
C	-1.349571	-0.002740	0.306095	1.307991	-0.000855	0.449559	1.180141	-0.000250	0.724925
H	-2.398128	-0.005615	0.570422	2.321691	-0.000123	0.825845	2.087873	0.000580	1.313088
C	-0.985747	-0.001099	-1.028954	1.090756	0.001027	-0.916891	1.264793	0.001160	-0.656462
C	4.010982	0.063254	-0.335305	-3.944186	-0.031589	-0.744544	-3.687113	-0.029469	-1.621950
H	3.979791	0.761920	0.502803	-3.942358	-0.212039	0.332474	-3.972205	-0.441998	-0.654956
H	4.733002	0.457809	-1.052680	-4.485086	-0.864366	-1.195091	-4.122095	-0.680860	-2.384378
C	4.572265	-1.261001	0.227488	-4.793606	1.229764	-1.031903	-4.344147	1.347833	-1.778482
H	5.520872	-1.031968	0.710878	-4.696106	1.460182	-2.097392	-3.887254	1.859905	-2.633886
C	4.931090	-2.303390	-0.843920	-4.278025	2.467226	-0.269521	-4.118277	2.230441	-0.556556
O	3.959536	-2.632575	-1.728751	-3.023501	2.873240	-0.563423	-4.390696	3.527583	-0.851165
H	3.211515	-2.015900	-1.656060	-2.577274	2.216794	-1.121427	-4.285509	4.038213	-0.034770
O	5.993358	-2.864214	-0.876315	-4.942982	3.075089	0.524344	-3.790179	1.869637	0.540262
N	3.684276	-1.814080	1.252605	-6.191413	0.939355	-0.764102	-5.803465	1.208722	-1.906603
H	2.787804	-2.085692	0.867124	-6.309493	0.711455	0.217461	-6.235677	2.107663	-2.080985
H	4.096870	-2.635116	1.677924	-6.750113	1.771816	-0.910989	-6.029262	0.604345	-2.686885
H	0.582646	0.036065	-3.434800	-0.216193	0.017638	-3.477485	0.554438	-0.036463	-3.437199

atom	37		
	x	y	z
H	-2.098623	-0.025383	-3.866896
C	-1.665569	-0.015897	-2.880716
C	-2.282324	-0.008579	-1.659254
C	-1.232801	0.001147	-0.675511
N	-0.296588	-0.010463	-2.718082
C	-0.000481	-0.001180	-1.375108
C	-1.214541	-0.000062	0.725493
H	2.160499	0.000377	-1.270134
C	0.003898	-0.001009	1.379909
H	-2.140591	0.008928	1.282539
H	0.030956	-0.002694	2.461195
C	1.214789	0.000998	0.664633
H	2.151087	0.002634	1.205861
C	1.229136	0.000107	-0.719416
C	-3.757251	0.027634	-1.402469
H	-3.975680	-0.309444	-0.389776
H	-4.275561	-0.653794	-2.082901
C	-4.382386	1.421670	-1.593229
H	-4.175068	1.757331	-2.614327
C	-3.718244	2.467120	-0.702866
O	-3.697520	2.111070	0.597936
H	-3.243467	2.816661	1.081014
O	-3.264327	3.508589	-1.103947
N	-5.811303	1.371614	-1.272553
H	-6.274033	2.235381	-1.527690
H	-6.260604	0.623575	-1.786257
H	0.375350	-0.013224	-3.462435

Table S22. Tables with the optimized Cartesian coordinates His($N^{\varepsilon}H$) tautomer conformers calculated at the B3LYP/D3/aug-cc-pVTZ .

atom	1			2			3		
	x	y	z	x	y	z	x	y	z
C	0.195023	0.241938	1.501632	0.183863	1.034860	-0.580596	-0.105687	-0.636574	0.602536
H	0.028263	1.064323	2.199283	0.465403	0.770177	-1.604143	-0.304434	-1.691679	0.421625
H	0.389730	-0.660332	2.077927	0.124283	2.121320	-0.538540	-0.287660	-0.460281	1.667808
C	1.451588	0.560347	0.665279	1.311740	0.605680	0.393521	-1.110040	0.201540	-0.201880
H	2.296575	0.674796	1.347633	0.862646	0.429008	1.374245	-0.829949	0.152340	-1.258453
N	1.349184	1.768349	-0.165475	2.267543	1.695687	0.533464	-1.179039	1.625185	0.152807
H	0.375553	1.996122	-0.348435	2.995259	1.419647	1.183047	-1.194592	1.725395	1.162967
H	1.783257	2.566124	0.275368	2.752682	1.826514	-0.348773	-0.329570	2.087996	-0.148690
C	1.814530	-0.653000	-0.216353	1.982408	-0.723976	-0.038423	-2.523676	-0.405307	-0.151998
O	1.830918	-1.782732	0.199672	3.162015	-0.783607	-0.283262	-2.742808	-1.585656	-0.087971
O	2.126989	-0.331881	-1.474822	1.203550	-1.804345	-0.111175	-3.501302	0.508621	-0.235777
H	2.010062	0.647034	-1.515164	0.243486	-1.594855	0.045937	-3.046246	1.378954	-0.242904
C	-1.015980	0.047223	0.651505	-1.159723	0.465575	-0.258307	1.314998	-0.323745	0.261647
C	-1.651667	-1.118503	0.323683	-2.379593	1.082602	-0.235560	2.311361	-1.198662	-0.078149
N	-1.620831	1.106490	-0.000926	-1.320080	-0.859932	0.088642	1.808098	0.967707	0.240747
C	-2.602599	0.588380	-0.700078	-2.600207	-1.044441	0.311015	3.070498	0.870010	-0.100350
N	-2.665482	-0.759047	-0.535010	-3.286973	0.107649	0.123439	3.428536	-0.424753	-0.303456
H	-1.458009	-2.138603	0.599790	-2.673771	2.096871	-0.433273	2.322482	-2.268896	-0.176694
H	-3.288669	1.130295	-1.328896	-3.066823	-1.968907	0.604644	3.761412	1.688499	-0.213930
H	-3.328281	-1.380940	-0.963304	-4.277843	0.230860	0.236050	4.338259	-0.756904	-0.570761
atom	4			5			6		
	x	y	z	x	y	z	x	y	z
C	0.154964	-1.089840	-0.890077	0.245454	0.390084	1.442457	-0.205680	0.783485	-1.249283
H	-0.048474	-2.157849	-0.965664	0.106168	1.279171	2.058660	-0.001127	1.779057	-1.641181
H	0.414262	-0.741956	-1.893788	0.425381	-0.455684	2.102994	-0.482094	0.135801	-2.079821
C	1.386897	-0.912966	0.025369	1.506807	0.634693	0.582104	-1.414816	0.913919	-0.292519
H	2.099244	-1.688781	-0.248537	2.364416	0.636481	1.263700	-2.250631	1.295972	-0.884166
N	1.024338	-1.136358	1.427848	1.435697	1.915937	-0.090346	-1.124594	1.864356	0.765641
H	0.441576	-0.386145	1.779209	2.113728	1.962638	-0.840452	-0.348067	1.523424	1.321851
H	1.856574	-1.167934	2.003777	0.518214	2.041834	-0.505244	-1.910884	1.934556	1.399959
C	2.181463	0.396109	-0.187947	1.748417	-0.546633	-0.358534	-1.852061	-0.458735	0.222378
O	3.374932	0.366419	-0.368777	1.851422	-0.487038	-1.555449	-1.849857	-0.801092	1.375978
O	1.526712	1.558853	-0.104912	1.878119	-1.713849	0.325930	-2.283646	-1.252234	-0.784089
H	0.544610	1.442299	-0.008928	2.055592	-2.407686	-0.325577	-2.493447	-2.117024	-0.402937
C	-1.094480	-0.412188	-0.425975	-0.982862	0.134846	0.632045	1.013850	0.237032	-0.583729
C	-2.366192	-0.906198	-0.343630	-1.684068	-1.033059	0.495802	2.087859	0.915369	-0.070875
N	-1.093398	0.897803	0.008672	-1.554432	1.113056	-0.158292	1.167352	-1.116039	-0.359508
C	-2.329447	1.192095	0.339857	-2.575855	0.548830	-0.753794	2.307837	-1.251758	0.269306
N	-3.141277	0.127106	0.139105	-2.701874	-0.755401	-0.389576	2.910472	-0.047902	0.470565
H	-2.774999	-1.873236	-0.571508	-1.549478	-2.004950	0.934321	2.330674	1.962169	-0.047647
H	-2.676987	2.136709	0.721288	-3.248967	1.026047	-1.446059	2.744100	-2.179964	0.598606
H	-4.129675	0.094972	0.317391	-3.408072	-1.396582	-0.704659	3.794442	0.109256	0.921230
atom	7			8			9		
	x	y	z	x	y	z	x	y	z
C	0.183038	1.035558	-0.582055	0.189301	-0.208591	-0.779753	0.301623	-1.257006	0.530918
H	0.463436	0.771203	-1.605994	0.420691	-1.196033	-1.183347	0.598766	-1.136487	1.574127
H	0.123434	2.121998	-0.539579	0.417383	0.534997	-1.545666	0.251452	-2.324194	0.315508
C	1.312042	0.606090	0.390687	1.098687	0.055361	0.459651	1.409252	-0.668361	-0.361612
H	0.863978	0.428753	1.371755	0.881880	-0.723760	1.190683	1.121897	-0.789718	-1.404232
N	2.267757	1.696185	0.530275	0.936812	1.357973	1.081822	2.660958	-1.384333	-0.129262
H	2.752057	1.827535	-0.352350	-0.044476	1.584620	1.179734	3.016704	-1.171166	0.796200
H	2.996160	1.419953	1.179015	1.382008	2.075305	0.521586	3.370187	-1.106987	-0.796465
C	1.982477	-0.723179	-0.042806	2.534829	-0.097041	0.007187	1.544750	0.821813	-0.056911
O	3.161813	-0.782432	-0.289021	3.253933	0.807606	-0.334107	1.925605	1.266284	0.998405
O	1.203745	-1.803683	-0.115368	2.915113	-1.393924	-0.038342	1.208743	1.609141	-1.102253
H	0.243814	-1.594435	0.042772	3.816814	-1.418321	-0.391153	1.319106	2.529625	-0.818993
C	-1.160105	0.466036	-0.258399	-1.254924	-0.146084	-0.419634	-1.028262	-0.621358	0.302273
C	-2.379918	1.083057	-0.233189	-2.180422	0.810761	-0.742371	-1.701430	0.265189	1.099039
N	-1.319948	-0.859803	0.087543	-1.838477	-1.108073	0.378715	-1.746040	-0.849504	-0.853557
C	-2.599752	-1.044533	0.311572	-3.088233	-0.746266	0.526487	-2.830465	-0.122319	-0.754310
N	-3.286784	0.107750	0.126158	-3.352090	0.413957	-0.134869	-2.855063	0.576951	0.413817
H	-2.674386	2.097512	-0.429501	-2.114535	1.701641	-1.340307	-1.464917	0.690575	2.057399
H	-3.065954	-1.969276	0.604981	-3.838741	-1.274630	1.090518	-3.623235	-0.062895	-1.481299
H	-4.277485	0.230852	0.240349	-4.236415	0.888654	-0.178891	-3.582877	1.195780	0.724306

atom	10			11			12		
	x	y	z	x	y	z	x	y	z
C	0.162969	-0.137969	-0.795723	-0.156966	-0.597574	0.609239	-0.135875	1.235552	-0.629810
H	0.413608	-1.121104	-1.196972	-0.373367	-1.656069	0.467205	0.123036	2.279854	-0.455041
H	0.354003	0.599062	-1.578387	-0.352467	-0.344289	1.651558	-0.337882	1.134314	-1.697359
C	1.093902	0.160584	0.405477	-1.097335	0.231870	-0.288800	-1.430297	0.942505	0.148709
H	0.812904	-0.521464	1.208083	-0.910338	-0.091706	-1.323535	-2.187235	1.626935	-0.244735
N	0.925511	1.529894	0.859138	-0.876867	1.655833	-0.079726	-1.266819	1.210092	1.564748
H	1.345295	1.660516	1.770576	-0.029895	1.954790	-0.542586	-0.471457	0.704734	1.934166
H	1.404317	2.162632	0.227627	-1.655773	2.197607	-0.430415	-2.077251	0.902388	2.087048
C	2.525321	-0.161347	-0.009724	-2.560480	-0.084809	-0.048869	-1.965807	-0.457504	-0.162084
O	3.354508	0.652167	-0.332121	-3.443429	0.730213	0.039396	-2.111874	-1.348839	0.636444
O	2.766893	-1.493077	-0.013311	-2.794929	-1.417364	0.005711	-2.294376	-0.587244	-1.468103
H	3.674584	-1.624430	-0.325179	-3.750408	-1.533014	0.110468	-2.629157	-1.486628	-1.598060
C	-1.275302	-0.114530	-0.406646	1.275746	-0.332489	0.284812	1.053158	0.395679	-0.269928
C	-2.252715	0.769241	-0.770868	2.252289	0.200979	1.080585	1.136205	-0.809058	0.381798
N	-1.793547	-1.056965	0.455494	1.791891	-0.607517	-0.965512	2.315941	0.811968	-0.637126
C	-3.057512	-0.749333	0.609060	3.053185	-0.255396	-0.921972	3.141112	-0.116057	-0.221600
N	-3.391445	0.352472	-0.116994	3.387569	0.241488	0.300014	2.478090	-1.123945	0.405294
H	-2.236861	1.637823	-1.403393	2.238271	0.549320	2.097159	0.391350	-1.452046	0.813686
H	-3.770425	-1.279789	1.217977	3.762643	-0.336295	-1.728648	4.211808	-0.110861	-0.340131
H	-4.296815	0.784800	-0.163891	4.292242	0.574966	0.581952	2.886134	-1.943443	0.818940
atom	13			14			15		
	x	y	z	x	y	z	x	y	z
C	-0.118635	0.829505	0.827348	-0.164789	-0.432617	0.670579	-0.151832	-0.424506	0.593283
H	-0.416460	0.352257	1.761849	-0.383626	-1.496188	0.780852	-0.345036	-1.493838	0.521719
H	0.007181	1.895503	1.018614	-0.397064	0.057443	1.618893	-0.355638	-0.135979	1.629960
C	-1.252884	0.669918	-0.206043	-1.092383	0.171992	-0.428064	-1.138111	0.338028	-0.313845
H	-0.821124	0.769942	-1.205225	-0.801784	-0.266623	-1.382722	-0.874435	0.128702	-1.351786
N	-2.342200	1.654592	-0.119380	-1.054117	1.623219	-0.550124	-1.052149	1.773465	-0.088542
H	-2.057113	2.545691	-0.503057	-0.087069	1.927667	-0.578804	-1.416748	1.993378	0.832321
H	-2.614063	1.812831	0.844488	-1.472815	2.055192	0.264864	-1.637601	2.271743	-0.747900
C	-1.867920	-0.741382	-0.172417	-2.497381	-0.333975	-0.165842	-2.540652	-0.217915	-0.073791
O	-1.285606	-1.721618	0.207965	-2.943959	-1.369626	-0.588296	-3.397715	0.319872	0.579913
O	-3.126766	-0.791745	-0.636199	-3.194346	0.471741	0.671484	-2.715967	-1.420019	-0.669687
H	-3.396631	0.139760	-0.773918	-4.059519	0.060462	0.811912	-3.605409	-1.731898	-0.446122
C	1.198570	0.298974	0.360699	1.281124	-0.265395	0.344823	1.290514	-0.205657	0.259122
C	1.703416	-0.970282	0.437735	2.200955	-1.238703	0.058504	1.948460	0.944067	-0.092641
N	2.094554	1.122153	-0.291652	1.879849	0.976839	0.256811	2.179207	-1.260891	0.292695
C	3.121531	0.366776	-0.596781	3.129316	0.755874	-0.072579	3.346886	-0.761927	-0.031097
N	2.934308	-0.913604	-0.174076	3.378271	-0.573933	-0.204384	3.262097	0.573454	-0.276845
H	1.282924	-1.879126	0.824514	2.123791	-2.310121	0.020712	1.597559	1.949802	-0.222104
H	4.014197	0.684992	-1.108653	3.886809	1.506452	-0.224214	4.271291	-1.309601	-0.107311
H	3.573089	-1.680192	-0.290760	4.259012	-0.995091	-0.441475	4.015701	1.179737	-0.548146
atom	16			17			18		
	x	y	z	x	y	z	x	y	z
C	0.178207	-1.138019	-0.795402	-0.181459	0.106640	-0.810879	-0.263112	0.524343	-1.426759
H	-0.028047	-2.209471	-0.822632	-0.386086	0.994833	-1.413330	-0.110351	1.463861	-1.960732
H	0.470804	-0.854754	-1.810983	-0.430835	-0.770452	-1.410991	-0.467598	-0.252649	-2.163389
C	1.390270	-0.903906	0.157316	-1.095732	0.139052	0.447747	-1.528581	0.703116	-0.530777
H	2.101957	-1.703559	-0.035173	-0.865549	1.048338	0.999194	-2.377781	0.839262	-1.202257
N	1.093338	-0.925442	1.588004	-0.956943	-0.995347	1.353147	-1.497451	1.822300	0.396305
H	0.400476	-0.232032	1.838812	-0.001352	-1.055331	1.681996	-1.431290	2.688683	-0.123539
H	0.741922	-1.831078	1.871799	-1.178387	-1.864891	0.883261	-0.669889	1.774228	0.979794
C	2.188486	0.372484	-0.176595	-2.528930	0.281456	-0.023489	-1.785316	-0.625459	0.162638
O	3.342313	0.316200	-0.516980	-3.111604	1.323467	-0.170648	-2.132408	-1.623217	-0.421029
O	1.555029	1.545583	-0.064060	-3.083276	-0.916161	-0.342139	-1.528808	-0.608747	1.485794
H	0.578169	1.438228	0.065830	-3.981186	-0.740952	-0.658641	-1.683588	-1.506751	1.813518
C	-1.081043	-0.430503	-0.409239	1.261810	0.073839	-0.439655	0.963797	0.176532	-0.654489
C	-2.367764	-0.893771	-0.405683	2.186828	-0.908724	-0.671883	1.586445	-1.039620	-0.559861
N	-1.076263	0.880028	0.025469	1.847503	1.108588	0.260050	1.610951	1.093902	0.151060
C	-2.323292	1.204516	0.275996	3.098426	0.763632	0.437364	2.601460	0.445735	0.713985
N	-3.147043	0.159138	0.026156	3.360696	-0.454405	-0.110535	2.631978	-0.854919	0.316879
H	-2.786877	-1.847900	-0.667633	2.119163	-1.854726	-1.177845	1.377495	-1.988371	-1.019649
H	-2.671060	2.158046	0.634503	3.850208	1.344266	0.945549	3.319074	0.858771	1.402930
H	-4.145799	0.153165	0.137154	4.245090	-0.930999	-0.111495	3.301765	-1.548840	0.598092

atom	19			20			21		
	x	y	z	x	y	z	x	y	z
C	0.232239	1.211240	-0.575575	-0.280758	-1.219120	-0.547761	-0.254639	-1.135020	-0.713205
H	0.537579	0.998804	-1.601936	-0.575132	-1.045929	-1.585030	-0.551554	-0.833816	-1.718602
H	0.143317	2.292579	-0.473077	-0.214549	-2.296412	-0.399053	-0.177443	-2.222418	-0.698844
C	1.359445	0.764104	0.374101	-1.425691	-0.722387	0.365088	-1.364266	-0.751179	0.277779
H	1.065633	0.981958	1.400037	-1.138165	-0.886057	1.403518	-1.063935	-1.058721	1.277527
N	2.575622	1.521711	0.074221	-2.627284	-1.498550	0.081494	-2.606690	-1.443502	-0.067293
H	2.925602	1.279709	-0.845510	-2.982952	-1.246917	-0.834964	-2.971101	-1.113713	-0.953165
H	3.308310	1.310931	0.740727	-3.360517	-1.276365	0.744088	-3.319082	-1.293377	0.636032
C	1.570805	-0.746566	0.320622	-1.635136	0.775675	0.155446	-1.556645	0.760528	0.346951
O	1.409466	-1.505281	1.241215	-2.290187	1.250645	-0.736707	-1.537281	1.429727	1.346971
O	2.017272	-1.145939	-0.894490	-1.008080	1.520895	1.090947	-1.820353	1.294696	-0.878056
H	2.086508	-2.110789	-0.874531	-1.114077	2.450249	0.838292	-1.978556	2.240513	-0.746919
C	-1.079130	0.552465	-0.304736	1.043351	-0.577821	-0.300853	1.060379	-0.523466	-0.362487
C	-2.159711	1.049367	0.373992	2.085788	-1.043092	0.455865	1.721782	0.505239	-0.977424
N	-1.337495	-0.740782	-0.708764	1.367108	0.653164	-0.831693	1.772986	-0.943349	0.741314
C	-2.544757	-1.018554	-0.284061	2.575234	0.925209	-0.406066	2.841969	-0.188852	0.789499
N	-3.091946	0.035643	0.380037	3.060124	-0.072692	0.382640	2.862676	0.709633	-0.233052
H	-2.343346	2.002420	0.836112	2.218883	-1.950066	1.017418	1.487197	1.091267	-1.847297
H	-3.068640	-1.949020	-0.425563	3.144348	1.810787	-0.634737	3.625244	-0.244836	1.526892
H	-4.006900	0.071053	0.793174	3.965625	-0.104637	0.816373	3.575844	1.394016	-0.411980
atom	22			23			24		
	x	y	z	x	y	z	x	y	z
C	0.291206	-1.195322	0.615094	-0.156076	-1.078401	-0.295198	0.177344	-0.563715	-0.570301
H	0.601576	-0.994699	1.641573	-0.442806	-1.096638	-1.350884	0.384979	-1.630162	-0.644688
H	0.218339	-2.277336	0.489137	-0.011222	-2.122869	-0.007438	0.428900	-0.107819	-1.530433
C	1.408658	-0.695350	-0.335902	-1.328357	-0.525507	0.535354	1.107904	0.027992	0.518472
H	1.092000	-0.857504	-1.364430	-0.979409	-0.311500	1.547797	0.960085	-0.556233	1.430960
N	2.708658	-1.329646	-0.135237	-2.434540	-1.495082	0.489982	0.792468	1.418479	0.785624
H	3.056107	-1.129027	0.795656	-2.097035	-2.447573	0.524373	0.452169	1.895247	-0.039013
H	2.633632	-2.333996	-0.234663	-3.077585	-1.364960	1.260867	1.586628	1.927366	1.146039
C	1.603169	0.795408	-0.112575	-1.836538	0.803647	-0.050921	2.538612	-0.290273	0.091710
O	2.055770	1.268055	0.900541	-1.380426	1.879398	0.233212	3.030513	-1.388928	0.157231
O	1.197711	1.545663	-1.155713	-2.831640	0.659983	-0.938085	3.210158	0.780250	-0.396242
H	1.342844	2.473877	-0.917164	-3.080393	-0.287753	-0.909556	4.087443	0.465868	-0.660438
C	-1.035586	-0.572826	0.341325	1.171131	-0.404331	-0.132426	-1.269680	-0.321021	-0.303762
C	-1.681182	0.411337	1.040866	1.498343	0.853767	0.298425	-2.154826	-1.087653	0.405496
N	-1.773474	-0.916092	-0.772544	2.316454	-1.101536	-0.461152	-1.900057	0.816674	-0.761862
C	-2.842653	-0.160243	-0.743793	3.313771	-0.282853	-0.236358	-3.136590	0.741872	-0.336038
N	-2.837703	0.666870	0.337515	2.872068	0.916869	0.227681	-3.345336	-0.395948	0.380625
H	-1.424751	0.936782	1.942838	0.890807	1.680418	0.614449	-2.048244	-2.030697	0.910267
H	-3.644221	-0.167461	-1.463437	4.358250	-0.501283	-0.383370	-3.914432	1.465170	-0.514684
H	-3.549216	1.331940	0.583634	3.438636	1.709402	0.472785	-4.210466	-0.686163	0.800709
atom	25			26			27		
	x	y	z	x	y	z	x	y	z
C	-0.164073	-0.492922	0.479030	0.093984	-1.300211	-0.564816	0.175807	-1.218893	0.120253
H	-0.324553	-1.558099	0.308036	-0.175771	-2.323759	-0.306406	0.436337	-1.398390	1.166369
H	-0.422051	-0.306311	1.525832	0.280198	-1.277939	-1.639871	0.039146	-2.205039	-0.331677
C	-1.126795	0.315505	-0.406614	1.408027	-0.962848	0.159785	1.372441	-0.555275	-0.586510
H	-0.816754	0.218182	-1.446018	2.133579	-1.714702	-0.162203	1.098481	-0.311626	-1.612192
N	-1.098294	1.734830	-0.049837	1.260477	-1.072684	1.600099	2.581128	-1.382337	-0.628836
H	-1.450702	1.869316	0.890701	0.506857	-0.488068	1.936774	2.858530	-1.657355	0.305931
H	-1.682896	2.278306	-0.672837	2.103244	-0.793410	2.083448	2.409239	-2.226380	-1.160512
C	-2.517304	-0.305932	-0.331040	1.992212	0.342591	-0.395376	1.741627	0.771250	0.060739
O	-3.061311	-0.915275	-1.215252	2.280658	0.513910	-1.551081	1.598321	1.859326	-0.434020
O	-3.099776	-0.098056	0.878769	2.189741	1.299623	0.549461	2.254851	0.592680	1.300197
H	-3.967070	-0.527931	0.860000	2.588805	2.057237	0.096216	2.478519	1.466065	1.652831
C	1.285262	-0.214754	0.230058	-1.074623	-0.418228	-0.244185	-1.123443	-0.479755	0.047890
C	1.922314	0.969227	-0.036356	-1.120647	0.854830	0.264811	-1.416737	0.738805	-0.506355
N	2.204704	-1.243213	0.260323	-2.355910	-0.860940	-0.497152	-2.266555	-1.036870	0.584425
C	3.369982	-0.695237	0.017860	-3.155409	0.118167	-0.155198	-3.227245	-0.175583	0.361035
N	3.254692	0.646942	-0.171448	-2.458171	1.185900	0.316122	-2.766700	0.921123	-0.298896
H	1.547471	1.969716	-0.136159	-0.353263	1.536390	0.582395	-0.804438	1.475837	-0.992224
H	4.313966	-1.211141	-0.036923	-4.230642	0.110275	-0.220454	-4.259498	-0.289595	0.647377
H	4.000618	1.287060	-0.378601	-2.842506	2.052743	0.647711	-3.305059	1.721533	-0.579118

atom	28			29			30		
	x	y	z	x	y	z	x	y	z
C	0.177122	-1.286098	0.013881	-0.273026	-1.102753	-0.714127	0.163366	-1.254082	0.084605
H	0.394836	-1.572203	1.045758	-0.604827	-0.783921	-1.703193	0.395472	-1.489487	1.126045
H	0.068940	-2.213552	-0.551133	-0.197081	-2.191955	-0.722042	0.032718	-2.212072	-0.425897
C	1.386733	-0.543588	-0.556982	-1.364191	-0.731545	0.323189	1.381623	-0.555389	-0.542457
H	1.169313	-0.205326	-1.569736	-1.009486	-1.008644	1.313685	1.140725	-0.229494	-1.553976
N	2.529134	-1.456398	-0.622577	-2.662368	-1.365458	0.099142	2.584228	-1.389333	-0.610761
H	2.815857	-1.720545	0.313669	-2.586000	-2.369858	0.196737	2.873217	-1.655173	0.324057
H	3.324416	-1.016730	-1.069438	-3.007061	-1.166313	-0.832188	2.396634	-2.237151	-1.131048
C	1.717466	0.674558	0.305074	-1.560235	0.774948	0.360589	1.776624	0.664671	0.278636
O	2.022993	0.625855	1.468309	-1.253619	1.508939	1.260274	2.162623	0.611156	1.416910
O	1.686009	1.838271	-0.393876	-2.122697	1.227590	-0.792804	1.666105	1.826557	-0.405519
H	1.930755	2.547970	0.219408	-2.240025	2.183605	-0.697549	1.951754	2.536819	0.189102
C	-1.109602	-0.525560	-0.016537	1.059308	-0.515149	-0.394854	-1.126488	-0.499269	0.020828
C	-1.402882	0.688118	-0.581668	1.692477	0.550115	-0.976645	-1.403736	0.728912	-0.520176
N	-2.240936	-1.050177	0.572308	1.814531	-0.991302	0.656231	-2.277498	-1.045498	0.550337
C	-3.194652	-0.176660	0.368418	2.880592	-0.232461	0.706849	-3.226571	-0.169547	0.335656
N	-2.741443	0.900321	-0.327890	2.858504	0.721157	-0.264032	-2.751713	0.928718	-0.311127
H	-0.801141	1.403605	-1.110702	1.423543	1.182829	-1.802882	-0.785208	1.460442	-1.005656
H	-4.216826	-0.266723	0.696466	3.691423	-0.323905	1.410089	-4.260193	-0.272603	0.621194
H	-3.277153	1.704729	-0.601358	3.559092	1.422108	-0.428105	-3.280530	1.738195	-0.583120
atom	31			32			33		
	x	y	z	x	y	z	x	y	z
C	-0.286486	-1.200271	-0.571517	0.129422	0.955039	-0.848625	0.186055	-0.869453	0.859563
H	-0.592081	-0.999128	-1.599458	0.400777	0.538073	-1.816934	0.441688	-0.409414	1.814094
H	-0.215714	-2.283245	-0.451736	0.003572	2.032322	-0.971520	0.125030	-1.944759	1.039277
C	-1.426772	-0.719176	0.373690	1.284738	0.727344	0.130431	1.349410	-0.676295	-0.142272
H	-1.109936	-0.882072	1.404168	0.925463	0.931715	1.147445	0.932697	-0.829737	-1.147340
N	-2.702526	-1.387830	0.152843	2.444945	1.549900	-0.243868	2.435331	-1.595627	0.180770
H	-3.125352	-1.053343	-0.705528	3.197431	1.431061	0.422942	3.310308	-1.269879	-0.209379
H	-2.579637	-2.389318	0.077722	2.186139	2.528826	-0.239653	2.240789	-2.519194	-0.180948
C	-1.645630	0.770342	0.171940	1.743269	-0.725316	0.115943	1.906123	0.735734	-0.170646
O	-2.318460	1.240137	-0.709292	1.363825	-1.594352	-0.623229	3.077923	1.014731	-0.225355
O	-0.980868	1.515886	1.075964	2.683112	-0.939660	1.071446	0.938876	1.680086	-0.172080
H	-1.090043	2.443570	0.818905	2.966438	-1.862737	0.998363	1.384896	2.537324	-0.235878
C	1.044815	-0.577446	-0.316650	-1.163370	0.385174	-0.365468	-1.141456	-0.388804	0.378128
C	2.066304	-1.035198	0.473139	-1.748647	-0.810445	-0.680952	-2.002168	0.498497	0.961913
N	1.389478	0.640989	-0.862968	-1.941844	1.059040	0.554309	-1.679842	-0.852078	-0.804195
C	2.589669	0.912420	-0.415263	-2.977214	0.289483	0.783748	-2.839324	-0.255556	-0.932713
N	3.049232	-0.073292	0.403246	-2.909687	-0.860210	0.057293	-3.086749	0.576341	0.115221
H	2.180473	-1.930846	1.056683	-1.431777	-1.610721	-1.323413	-1.935368	1.074913	1.866446
H	3.170426	1.789317	-0.648028	-3.795780	0.507775	1.449086	-3.533445	-0.385580	-1.746166
H	3.943574	-0.101967	0.859860	-3.582543	-1.606072	0.056086	-3.906057	1.142081	0.247566
atom	34			35			36		
	x	y	z	x	y	z	x	y	z
C	0.273156	0.394857	1.438400	-0.184586	-0.397229	0.675729	-0.173355	-0.088901	0.800935
H	0.142667	1.289072	2.048643	-0.418971	-1.456986	0.801110	-0.380556	-1.091615	1.180944
H	0.464201	-0.436299	2.118816	-0.399151	0.099576	1.624160	-0.366857	0.608503	1.616678
C	1.510460	0.641402	0.537515	-1.119837	0.230455	-0.403783	-1.139979	0.243891	-0.364782
H	2.391399	0.676180	1.191684	-0.859666	-0.222760	-1.367865	-0.744818	-0.236093	-1.263423
N	1.392068	1.911928	-0.144885	-1.038652	1.671960	-0.529789	-1.180507	1.676634	-0.591467
H	0.448061	2.033451	-0.496957	-0.083573	1.983217	-0.396268	-1.763770	2.111778	0.115658
H	2.013146	1.944259	-0.943550	-1.624061	2.107604	0.173427	-1.614618	1.884940	-1.481912
C	1.722598	-0.555106	-0.406045	-2.549366	-0.166282	-0.047855	-2.511726	-0.370382	-0.062381
O	1.633259	-0.510004	-1.598423	-3.284968	0.503956	0.618930	-3.463349	0.255181	0.313626
O	2.046693	-1.732156	0.203698	-2.946186	-1.394605	-0.473874	-2.596806	-1.717689	-0.214765
H	2.130407	-1.610837	1.156554	-2.276115	-1.783957	-1.047537	-1.759076	-2.079195	-0.531182
C	-0.971670	0.120564	0.660704	1.261031	-0.259253	0.333894	1.251767	-0.025133	0.372694
C	-1.605124	-1.075797	0.457389	2.171786	-1.248743	0.074654	2.226021	0.874585	0.701933
N	-1.617403	1.115313	-0.045312	1.870908	0.973786	0.211925	1.752491	-0.955695	-0.514050
C	-2.617575	0.532421	-0.659215	3.117694	0.733272	-0.111936	3.005084	-0.626099	-0.713923
N	-2.657155	-0.798818	-0.385208	3.354895	-0.601712	-0.207439	3.345645	0.478765	0.003060
H	-1.398490	-2.068844	0.812706	2.089054	-2.320509	0.075069	2.219054	1.740466	1.338069
H	-3.334505	1.014870	-1.301914	3.881172	1.473023	-0.285006	3.703643	-1.142138	-1.351073
H	-3.325594	-1.460633	-0.738135	4.232791	-1.036741	-0.429814	4.244425	0.927306	0.016304

Table S23. Tables with the optimized Cartesian coordinates His(N$^{\delta}$H) tautomer conformers calculated at the B3LYP/D3/aug-cc-pVTZ .

atom	1			2			3		
	x	y	z	x	y	z	x	y	z
C	2.345501	0.007369	1.302747	-2.406491	-0.004525	1.182551	2.660695	-0.010583	-0.302208
H	2.187349	-0.280990	2.340702	-3.037524	-0.762959	0.711140	2.953852	0.800814	-0.971332
H	3.012277	-0.729798	0.853415	-2.307949	-0.275930	2.232394	3.156119	0.154080	0.653035
C	3.055698	1.372499	1.319894	-3.144918	1.338635	1.140042	3.193530	-1.322355	-0.935912
H	3.957255	1.272548	1.933499	-2.521631	2.109194	1.594028	4.272047	-1.202625	-1.053982
N	2.208988	2.380183	1.945405	-4.383994	1.248756	1.919687	2.587890	-1.565410	-2.242744
H	1.306467	2.426166	1.486616	-5.023288	0.575905	1.512264	3.247940	-2.014960	-2.862944
H	2.633379	3.297217	1.918380	-4.857453	2.142005	1.967299	1.816272	-2.215644	-2.135444
C	3.576008	1.759641	-0.067995	-3.460552	1.774893	-0.287837	2.979024	-2.491070	0.022079
O	3.545516	1.079666	-1.066665	-3.538578	1.051677	-1.253672	2.285292	-3.448826	-0.199292
O	4.126725	2.993013	-0.058891	-3.731060	3.096443	-0.350655	3.656896	-2.321469	1.178878
H	4.447997	3.181327	-0.953067	-3.988985	3.305439	-1.260992	3.450684	-3.074211	1.752713
C	1.021743	0.000432	0.613795	-1.051472	0.001067	0.561240	1.188570	-0.000697	-0.085862
C	-0.261408	0.000184	1.099054	0.204834	-0.000595	1.110489	0.419983	-0.000177	1.048772
C	-0.459369	0.001082	-1.027392	0.511548	0.000857	-1.002594	-0.968620	-0.001540	-0.574947
N	-1.175363	-0.000774	0.071610	1.169768	-0.000151	0.131482	-0.918852	0.001050	0.734646
H	-0.569015	-0.009350	2.131423	0.460896	-0.011988	2.156991	0.762084	0.007178	2.070230
H	-0.845016	0.002849	-2.033354	0.947956	0.001819	-1.987568	-1.863634	-0.004735	-1.174520
H	1.640450	0.055327	-1.408566	-1.563232	0.035703	-1.494803	0.526543	-0.046068	-2.096749
N	0.874398	-0.000924	-0.757067	-0.834677	-0.001178	-0.800617	0.278918	0.001363	-1.122609
atom	4			5			6		
	x	y	z	x	y	z	x	y	z
H	-2.653171	0.017013	0.364827	-2.445337	0.038930	-1.099632	-2.387783	0.010586	1.219329
H	-2.884819	-0.171368	1.413253	-2.406776	-0.290676	-2.136467	-3.040860	-0.724564	0.742147
C	-3.063849	1.002972	0.122326	-3.100584	-0.644863	-0.560279	-2.279473	-0.283871	2.263674
H	-3.379997	-1.024984	-0.515346	-3.076276	1.449613	-1.101540	-3.108234	1.374378	1.199037
N	-2.911545	-1.996131	-0.363935	-4.101464	1.339760	-1.472928	-2.484608	2.123407	1.685954
H	-3.255805	-0.663070	-1.930831	-2.354159	2.327982	-1.998649	-4.415576	1.384231	1.857001
H	-3.808901	0.162541	-2.130798	-1.368143	2.332535	-1.759894	-4.987065	0.610162	1.539746
C	-3.604247	-1.404926	-2.525380	-2.681140	3.281569	-1.905489	-4.317102	1.310948	2.860801
O	-4.811747	-1.169871	-0.012881	-3.243777	2.026882	0.307060	-3.358924	1.845494	-0.229601
O	-5.196702	-2.024215	0.740937	-3.023344	3.164744	0.622093	-3.596523	1.111298	-1.161025
H	-5.618807	-0.186019	-0.485088	-3.733300	1.112282	1.193072	-3.317035	3.181503	-0.344532
C	-6.496317	-0.316931	-0.096815	-3.906776	1.585892	2.020649	-3.540747	3.409627	-1.259519
C	-1.171669	-0.001891	0.205475	-1.067566	-0.001087	-0.533333	-1.040444	0.000298	0.582065
C	-0.151147	-0.000052	1.120656	0.172959	0.000942	-1.116123	0.226450	-0.000010	1.106330
N	0.797436	-0.003435	-0.793875	0.541431	-0.000296	0.988610	0.492259	0.000509	-1.012512
H	1.070220	0.002131	0.488839	1.165048	-0.000406	-0.162811	1.171949	-0.000305	0.108476
H	-0.232396	0.003192	2.194905	0.399056	-0.005876	-2.169322	0.503907	-0.009756	2.147439
H	1.519471	-0.014835	-1.593314	1.005313	-0.003254	1.960931	0.909365	0.000426	-2.005785
N	-1.043776	-0.083655	-1.893644	-1.511986	0.002023	1.545291	-1.589659	0.027900	-1.467201
H	-0.544841	0.003247	-1.021095	-0.811873	0.000848	0.823656	-0.850214	-0.000493	-0.784359
atom	7			8			9		
	x	y	z	x	y	z	x	y	z
H	2.649558	0.005939	-0.400301	0.202694	0.055447	2.619490	2.674461	0.058854	0.113735
H	2.979652	0.989941	-0.749672	-0.733640	-0.204715	3.115291	3.036066	-0.645442	0.867346
C	3.173268	-0.194424	0.532568	0.961585	-0.665275	2.930245	3.111741	-0.229570	-0.842762
H	3.106460	-1.026605	-1.454265	0.652412	1.447501	3.132336	3.180158	1.495678	0.471346
N	4.203243	-1.041624	-1.443375	1.566557	1.732875	2.615294	2.764802	1.761431	1.446014
H	2.559837	-0.681030	-2.772225	0.880453	1.521713	4.572887	2.842111	2.525804	-0.488781
H	2.675342	-1.438252	-3.432898	0.020242	1.330246	5.073816	1.890255	2.415757	-0.815719
C	3.042396	0.128784	-3.141384	1.571117	0.839678	4.859359	3.467634	2.488760	-1.284781
O	2.713539	-2.421061	-0.988985	-0.433111	2.458767	2.802260	4.685854	1.420802	0.618137
O	3.078663	-2.889122	0.056286	-1.524153	2.462890	3.316329	5.481614	1.703667	-0.238709
H	1.935939	-3.091710	-1.867692	-0.063680	3.344273	1.856330	5.045147	0.925546	1.825689
C	1.740430	-3.955372	-1.474313	-0.818490	3.932194	1.700921	6.010841	0.849117	1.833030
C	1.182237	-0.001873	-0.133146	0.040784	-0.001126	1.138480	1.194157	-0.002210	0.004407
C	0.461235	-0.000371	1.032526	-1.113397	0.000817	0.401890	0.348099	0.001633	-1.073835
N	-0.988884	-0.004012	-0.536964	1.133354	0.001016	0.296268	-0.929461	-0.001080	0.638262
H	-0.888657	0.002677	0.771099	0.650313	-0.000515	-0.921077	-0.967325	-0.000359	-0.669781
H	0.843177	-0.003188	2.039679	-0.711054	-0.000192	-0.915560	0.619787	-0.005987	-2.116194
H	-1.907206	-0.016444	-1.100094	-2.148825	0.007744	0.690412	-1.780970	-0.007164	1.298213
N	0.468049	-0.098309	-2.109920	1.225552	-0.005372	-1.831860	0.639931	-0.009843	2.064759
H	0.234069	0.003579	-1.133515	-1.311262	-0.009968	-1.721032	0.354530	0.002015	1.100947

atom	10			11			12		
	x	y	z	x	y	z	x	y	z
C	0.152909	0.053428	2.674036	2.327807	0.013699	-1.331049	-2.170990	-0.030809	-1.584568
H	-0.820938	-0.168541	3.112753	3.008312	-0.715156	-0.883375	-1.940999	0.571263	-2.466093
H	0.850456	-0.699361	3.046441	2.178352	-0.287731	-2.366833	-2.352972	-1.051423	-1.927291
C	0.625826	1.418928	3.228177	3.020834	1.384217	-1.364686	-3.478303	0.529348	-0.995877
H	1.617507	1.642652	2.834516	2.370802	2.102719	-1.861366	-4.226907	0.457298	-1.790524
N	0.707179	1.343180	4.679354	4.262227	1.286736	-2.137081	-3.331752	1.924469	-0.634196
H	-0.226090	1.282817	5.072478	4.933385	0.680700	-1.679956	-2.596994	2.033862	0.054774
H	1.133573	2.177525	5.063145	4.691976	2.194711	-2.262470	-4.180856	2.273036	-0.207437
C	-0.339102	2.495891	2.736434	3.309779	1.976410	0.010578	-4.024084	-0.354408	0.131754
O	-1.421033	2.705597	3.222360	3.236279	3.142438	0.292108	-4.299339	0.022486	1.240407
O	0.146882	3.180225	1.679836	3.768443	1.045410	0.901323	-4.207197	-1.632027	-0.277998
H	-0.543242	3.790573	1.378063	4.043119	1.528410	1.695097	-4.564456	-2.131118	0.471346
C	0.024357	-0.000612	1.192856	1.013367	-0.000165	-0.629453	-1.003579	-0.000274	-0.648386
C	-1.067525	0.000702	0.366491	-0.275305	0.000102	-1.094745	-0.873365	-0.000068	0.717662
C	0.622805	0.000129	-0.939731	-0.447457	-0.000115	1.034202	1.128087	-0.000600	-0.036598
N	-0.686052	-0.000516	-0.955473	-1.175079	0.000006	-0.054823	0.454700	0.000410	1.084203
H	-2.104577	0.003680	0.657558	-0.599086	-0.005954	-2.122198	-1.655774	0.004926	1.456853
H	1.268510	-0.004667	-1.802103	-0.819436	-0.000867	2.045245	2.200903	0.001764	-0.134636
H	2.074879	0.001271	0.604252	1.648994	0.006076	1.397699	0.570133	0.013140	-2.083293
N	1.106415	0.000297	0.335857	0.884474	0.000171	0.744820	0.294157	0.000532	-1.116881
atom	13			14			15		
	x	y	z	x	y	z	x	y	z
H	-0.283054	0.054507	-2.665460	2.502165	-0.008551	-0.962180	-2.676456	0.063586	0.000529
H	0.482308	-0.569888	-3.126698	3.005435	-0.945628	-0.703764	-3.068992	-0.611900	-0.764241
C	-1.256041	-0.331893	-2.967086	2.452707	0.030288	-2.048917	-3.079565	-0.260042	0.960797
H	-0.158143	1.501278	-3.201949	3.393069	1.196766	-0.530749	-3.188883	1.512144	-0.288846
N	0.829793	1.881685	-2.891261	2.736382	2.021792	-0.250297	-2.777598	1.828589	-1.247605
H	-1.269194	2.303349	-2.730749	4.221091	1.626969	-1.643554	-2.838725	2.506899	0.710607
H	-1.203867	2.472540	-1.736811	4.772912	2.436839	-1.387234	-1.839039	2.496777	0.875563
C	-1.336311	3.181144	-3.227205	4.886717	0.901044	-1.885736	-3.299473	2.316353	1.592010
O	-0.110697	1.553099	-4.719547	4.216598	0.826473	0.702497	-4.687120	1.428493	-0.503259
O	-0.740728	2.308529	-5.411121	5.406532	0.663399	0.723133	-5.210789	1.190565	-1.560668
H	0.786372	0.671938	-5.226295	3.451484	0.673612	1.821740	-5.384121	1.568824	0.647879
C	0.792995	0.791540	-6.187163	4.048953	0.428586	2.544396	-6.323098	1.465700	0.434525
C	-0.176198	-0.002111	-1.181307	1.101631	0.001216	-0.454631	-1.193165	-0.002576	0.048665
C	-1.114284	0.001382	-0.182840	-0.093968	0.000222	-1.125025	-0.300081	0.001861	1.088250
N	0.771622	-0.001353	0.823198	-0.608855	0.002535	0.946543	0.900208	-0.001334	-0.679083
H	-0.516053	-0.000031	1.056962	-1.151109	-0.001686	-0.245974	0.996033	-0.000345	0.626224
H	-2.185162	0.001856	-0.296019	-0.243949	-0.003088	-2.191796	-0.524770	-0.005455	2.141744
H	1.552385	-0.009902	1.565543	-1.140069	0.010545	1.883846	1.721556	-0.008549	-1.376174
N	1.943756	-0.024409	-0.944679	1.403059	0.057266	1.643581	-0.732047	-0.014397	-2.033963
H	1.034912	0.002113	-0.516013	0.752301	-0.002286	0.879086	-0.402995	0.002393	-1.084056
atom	16			17			18		
	x	y	z	x	y	z	x	y	z
H	-0.072540	0.027071	-2.679597	2.677089	0.012372	0.003669	-2.659593	0.007286	0.303134
H	0.933658	-0.105286	-3.077956	3.042502	-0.771362	0.671438	-2.914621	-0.131906	1.354216
C	-0.679962	-0.794764	-3.065422	3.064278	-0.213166	-0.991288	-3.076432	0.977164	0.011979
H	-0.660477	1.331826	-3.248885	3.273774	1.372187	0.458458	-3.353305	-1.068903	-0.567352
N	-1.690552	1.446471	-2.911694	2.812344	1.648212	1.408386	-2.854491	-2.026243	-0.394017
H	-0.669349	1.269789	-4.708072	2.990291	2.405838	-0.513657	-3.210080	-0.732952	-1.980546
H	0.275641	1.251374	-5.072974	3.254601	3.318074	-0.164568	-3.849787	0.024027	-2.202689
C	-1.137818	2.072777	-5.108709	3.527568	2.249577	-1.358967	-3.497616	-1.513829	-2.558451
O	0.078871	2.548558	-2.698578	4.763696	1.164787	0.721476	-4.820014	-1.190273	-0.132782
O	-0.422796	3.437850	-2.062180	5.652146	1.475209	-0.028035	-5.732696	-0.701828	-0.736442
H	1.389950	2.536441	-3.044262	4.981634	0.545875	1.908202	-5.046047	-1.862181	1.023676
C	1.798904	3.328890	-2.667220	5.936524	0.407707	1.996973	-4.226049	-2.217422	1.387375
C	0.010198	-0.000651	-1.193322	1.191827	-0.000947	-0.048144	-1.175253	-0.001736	0.180198
C	1.077282	0.001142	-0.335266	0.301880	0.001033	-1.087182	-0.174677	0.000140	1.117475
N	-0.649335	0.000817	0.921304	-0.901158	0.000110	0.678047	0.813752	-0.002826	-0.776766
H	0.658376	-0.001211	0.974804	-0.994558	-0.000712	-0.627265	1.059476	0.001639	0.511541
H	2.122258	0.002495	-0.596326	0.528685	0.010229	-2.139831	-0.278137	0.009406	2.189976
H	-1.319714	0.003028	1.764574	-1.723138	-0.002894	1.374474	1.552566	-0.012038	-1.560692
N	-2.057227	0.013904	-0.661893	0.402010	0.000515	1.084544	-1.005602	-0.078725	-1.915208
H	-1.096521	-0.000098	-0.367520	0.727701	-0.016225	2.035144	-0.523298	0.002783	-1.032447

atom	19			20			21		
	x	y	z	x	y	z	x	y	z
C	2.343798	-0.007459	1.304498	2.168463	-0.027731	1.588513	2.596767	-0.019889	-0.667463
H	2.132265	0.019188	2.373819	1.925841	0.546357	2.484718	2.903163	0.965071	-1.034407
H	2.889125	-0.938923	1.123713	2.381058	-1.052365	1.900320	3.213294	-0.218693	0.210112
C	3.292231	1.198786	0.988893	3.460252	0.572302	1.005043	2.928468	-1.037542	-1.770407
H	4.016824	1.249642	1.799707	4.200122	0.542984	1.809725	4.020991	-1.075114	-1.865794
N	2.660761	2.504259	0.866581	3.267980	1.958982	0.625545	2.282576	-0.645211	-3.036146
H	1.933335	2.494038	0.161933	2.549817	2.044441	-0.082672	2.387421	-1.397349	-3.708395
H	2.223658	2.764709	1.741743	4.116097	2.357095	0.245594	2.743741	0.170036	-3.422592
C	4.140754	0.891615	-0.233240	4.046137	-0.383370	-0.045355	2.544620	-2.507982	-1.510099
O	5.234418	0.396282	-0.201363	4.279273	-1.545716	0.164034	2.793745	-3.344855	-2.336293
O	3.517395	1.186701	-1.409640	4.314210	0.216055	-1.228584	1.971277	-2.842581	-0.344325
H	4.146647	0.987020	-2.118992	4.697422	-0.458937	-1.808588	1.636443	-2.063284	0.133006
C	1.029104	0.000709	0.604172	1.000989	-0.000348	0.652952	1.163780	-0.000609	-0.249137
C	-0.248845	0.000931	1.100226	0.875506	-0.000156	-0.713065	0.558658	-0.000725	0.984696
C	-0.472582	0.002864	-1.021838	-1.128127	-0.000881	0.031635	-1.035430	-0.002351	-0.437935
N	-1.174453	-0.002329	0.083902	-0.450033	0.000636	-1.086681	-0.809860	0.001888	0.853953
H	-0.545884	-0.007291	2.135894	1.661207	0.001022	-1.448678	1.035698	0.011355	1.951058
H	-0.868228	0.012067	-2.023901	-2.201289	0.000638	0.125189	-2.004443	-0.013968	-0.908285
H	1.608691	0.087429	-1.439830	-0.577934	0.009719	2.080578	0.264702	-0.087192	-2.148625
N	0.866776	-0.002174	-0.766462	-0.298334	0.000749	1.115158	0.122852	0.001798	-1.151577
atom	22			23			24		
	x	y	z	x	y	z	x	y	z
H	-2.637392	-0.006309	0.459981	-2.617139	0.055677	-0.576204	-0.023664	0.025872	-2.680558
H	-2.899124	0.831587	1.111940	-2.833276	-0.595802	-1.427286	1.000303	0.019580	-3.054591
C	-3.185928	0.136254	-0.472437	-3.203071	-0.310555	0.266450	-0.513562	-0.869997	-3.075461
H	-3.130358	-1.314900	1.128529	-3.130933	1.483624	-0.943014	-0.757434	1.251841	-3.287100
N	-2.555400	-1.477885	2.040831	-4.208667	1.402201	-1.091889	-1.806805	1.226919	-2.994427
H	-2.924910	-2.449936	0.248885	-2.553995	2.087690	-2.132522	-0.701231	1.334259	-4.743158
H	-3.461603	-2.355842	-0.604281	-2.674314	1.494420	-2.942567	-1.131668	0.522019	-5.166782
C	-3.197357	-3.318409	0.689993	-1.572035	2.298050	-2.013950	0.257589	1.385328	-5.065511
O	-4.561814	-1.076713	1.602297	-2.960936	2.359686	0.288462	-0.208161	2.535032	-2.684638
O	-4.865070	-0.691921	2.702635	-3.612899	2.222303	1.291670	-0.783856	3.234040	-1.895540
H	-5.473690	-1.306173	0.626791	-1.965515	3.260150	0.171973	1.046425	2.789055	-3.126292
C	-6.346683	-1.096763	0.990043	-1.885553	3.715830	1.022939	1.350002	3.601480	-2.695623
C	-1.181767	0.000218	0.158397	-1.174430	-0.001571	-0.217666	0.027691	0.000638	-1.192829
C	-0.484577	-0.000621	-1.018867	-0.534618	0.000576	0.994314	1.082203	0.000899	-0.318972
N	1.004089	-0.000687	0.512511	1.029509	-0.001773	-0.461937	-0.662701	0.002677	0.911223
H	0.871757	0.000808	-0.789459	0.830877	0.000727	0.831849	0.643942	-0.002193	0.984470
H	-0.889229	-0.005143	-2.016994	-0.990402	0.002577	1.970194	2.131142	0.000025	-0.564145
H	1.933581	0.000229	1.057148	1.985461	-0.009509	-0.958590	-1.345720	0.011229	1.744211
N	-0.366450	0.006756	2.130074	-0.261469	-0.021571	-2.144777	-2.046839	0.050614	-0.689275
H	-0.209502	0.000282	1.137420	-0.151338	0.002041	-1.146560	-1.091135	-0.002022	-0.383892
atom	25			26			27		
	x	y	z	x	y	z	x	y	z
H	2.154731	-0.015854	1.602320	2.465677	0.034412	-1.059153	2.251047	-0.017779	1.462459
H	1.962690	0.647196	2.450406	3.176782	-0.530613	-0.459435	2.215397	0.802914	2.185025
C	2.306227	-1.020727	2.009598	2.422975	-0.448080	-2.039559	2.284573	-0.945473	2.043664
H	3.480232	0.426105	0.955274	3.014430	1.454332	-1.253425	3.582875	0.075907	0.693484
N	3.685895	-0.185493	0.078376	2.212670	2.100126	-1.636634	3.628166	-0.715107	-0.052878
H	4.640591	0.350445	1.840788	4.198646	1.430963	-2.121706	4.771679	-0.032519	1.538775
H	4.503475	0.938264	2.654896	4.564767	2.365129	-2.256457	4.755497	0.656288	2.280924
C	4.796699	-0.598541	2.155018	3.961732	1.060013	-3.033357	4.834777	-0.950927	1.958319
O	3.375526	1.883173	0.520673	3.456343	2.054105	0.077801	3.700274	1.379450	-0.086178
O	3.123333	2.784349	1.279323	3.562230	1.474871	1.123603	3.817546	1.476914	-1.276817
H	3.614574	2.059873	-0.791498	3.753333	3.368916	-0.071659	3.671203	2.450944	0.745061
C	3.532788	3.007006	-0.981146	4.062447	3.698096	0.785317	3.773581	3.244505	0.199789
C	0.990705	-0.000014	0.666363	1.117074	-0.002262	-0.422185	1.033036	-0.000288	0.598123
C	0.886244	0.000039	-0.700569	0.703888	0.001423	0.883949	0.838526	0.000141	-0.758840
N	-1.128071	0.000042	0.015478	-1.095660	-0.001530	-0.272272	-1.124745	-0.000265	0.090568
H	-0.433801	-0.000050	-1.092977	-0.669870	0.000048	0.965525	-0.505846	0.000074	-1.060898
H	1.683892	0.013994	-1.422722	1.328551	0.016612	1.759673	1.587853	0.017628	-1.530937
H	-2.202570	0.003304	0.093033	-2.124659	-0.009645	-0.591423	-2.191810	0.000453	0.239538
N	-0.609403	0.020436	2.072461	-0.126309	-0.024379	-2.157047	-0.472355	0.003669	2.109016
H	-0.315077	-0.000017	1.111705	-0.055433	0.002321	-1.155017	-0.240971	0.000338	1.131048

atom	28			29		
	x	y	z	x	y	z
C	-2.533835	-0.023648	-0.890856	-2.334604	0.034741	1.319206
H	-3.130232	0.771022	-0.440170	-2.398709	-0.836246	1.979584
H	-2.489549	0.180753	-1.962994	-2.308249	0.912313	1.976305
C	-3.275727	-1.368365	-0.706459	-3.634073	0.128367	0.500663
H	-2.540521	-2.176760	-0.733227	-3.513557	0.883677	-0.278241
N	-4.291494	-1.686818	-1.718542	-4.744580	0.405379	1.423811
H	-3.871870	-1.976427	-2.591414	-4.494749	1.104636	2.110709
H	-4.886884	-0.889231	-1.910516	-5.561462	0.740922	0.928305
C	-3.927911	-1.469812	0.686163	-3.920769	-1.205087	-0.212650
O	-3.565820	-0.840934	1.641563	-3.525794	-1.469057	-1.313187
O	-4.937039	-2.353626	0.746653	-4.641722	-2.069949	0.523610
H	-5.108700	-2.637444	-0.172781	-4.934288	-1.579557	1.317243
C	-1.145752	0.002120	-0.336632	-1.072779	0.000553	0.520799
C	-0.636001	-0.001075	0.935843	-0.782581	-0.000766	-0.817772
C	1.072570	0.001885	-0.354376	1.115497	-0.000347	0.171593
N	0.739952	-0.000483	0.911416	0.580004	0.000691	-1.020760
H	-1.193788	-0.014676	1.854528	-1.477435	-0.038022	-1.636923
H	2.074661	0.008548	-0.750105	2.169029	-0.002311	0.397133
H	-0.034727	0.027392	-2.160689	0.322535	-0.003545	2.137860
N	-0.030770	-0.002446	-1.156250	0.159859	-0.000131	1.146141

Table S24. Tables with the distance of atoms in the rings and HOMA index for: Phe, Tyr, Trp and His.

Phe

molecule	R1	R2	R3	R4	R5	R6	HOMA
1	1.3963	1.3916	1.3892	1.3915	1.3891	1.3980	0.997
2	1.3969	1.3882	1.3923	1.3888	1.3922	1.3948	0.997
3	1.3968	1.3889	1.3917	1.3894	1.3916	1.3944	0.998
4	1.3963	1.3904	1.3903	1.3905	1.3895	1.3964	0.998
5	1.3963	1.3906	1.3902	1.3908	1.3897	1.3972	0.997
6	1.3968	1.3886	1.3918	1.3893	1.3916	1.3951	0.998
7	1.3950	1.3906	1.3900	1.3911	1.3892	1.3956	0.998
8	1.3964	1.3896	1.3911	1.3902	1.3909	1.3953	0.998
9	1.3955	1.3900	1.3904	1.3907	1.3898	1.3944	0.999
10	1.3968	1.3892	1.3914	1.3897	1.3914	1.3954	0.998
11	1.3945	1.3908	1.3896	1.3912	1.3888	1.3960	0.998
12	1.3968	1.3888	1.3917	1.3894	1.3915	1.3951	0.998
13	1.3953	1.3903	1.3902	1.3909	1.3893	1.3956	0.998
14	1.3954	1.3897	1.3907	1.3904	1.3899	1.3939	0.999
15	1.3932	1.3894	1.3899	1.3907	1.3891	1.3948	0.999
16	1.3944	1.3900	1.3906	1.3902	1.3900	1.3963	0.998
17	1.3947	1.3903	1.3897	1.3910	1.3889	1.3957	0.998
18	1.3947	1.3906	1.3902	1.3907	1.3896	1.3964	0.998
19	1.3951	1.3905	1.3898	1.3912	1.3899	1.3987	0.997
20	1.3944	1.3907	1.3898	1.3910	1.3891	1.3963	0.998
21	1.3992	1.3896	1.3911	1.3897	1.3911	1.3981	0.995

Tyr

molecule	R1	R2	R3	R4	R5	R6	HOMA
1	1.3967	1.3886	1.3906	1.3932	1.3878	1.3969	0.996
2	1.3997	1.3852	1.3932	1.3908	1.3915	1.3940	0.995
3	1.3982	1.3846	1.3941	1.3904	1.3919	1.3928	0.996
4	1.3973	1.3862	1.3918	1.3916	1.3899	1.3944	0.997
5	1.3971	1.3871	1.3915	1.3920	1.3889	1.3946	0.997
6	1.3953	1.3883	1.3909	1.3931	1.3886	1.3949	0.998
7	1.3979	1.3855	1.3933	1.3908	1.3914	1.3923	0.996
8	1.3971	1.3875	1.3913	1.3922	1.3892	1.3955	0.997
9	1.3982	1.3864	1.3921	1.3915	1.3903	1.3943	0.996
10	1.3979	1.3852	1.3933	1.3907	1.3913	1.3931	0.996
11	1.3965	1.3860	1.3923	1.3912	1.3902	1.3932	0.997
12	1.3960	1.3873	1.3911	1.3924	1.3889	1.3937	0.998
13	1.3959	1.3873	1.3912	1.3920	1.3890	1.3932	0.998
14	1.3971	1.3865	1.3922	1.3915	1.3904	1.3935	0.997
15	1.3960	1.3863	1.3918	1.3914	1.3901	1.3932	0.998
16	1.3964	1.3876	1.3914	1.3923	1.3894	1.3943	0.998
17	1.3953	1.3875	1.3908	1.3926	1.3884	1.3942	0.998

18	1.3969	1.3856	1.3924	1.3910	1.3903	1.3926	0.997
19	1.3959	1.3883	1.3908	1.3932	1.3882	1.3951	0.998
20	1.3979	1.3854	1.3932	1.3909	1.3912	1.3931	0.996
21	1.3962	1.3870	1.3914	1.3922	1.3889	1.3937	0.998
22	1.3963	1.3882	1.3909	1.3927	1.3889	1.3948	0.998
23	1.3966	1.3876	1.3911	1.3924	1.3887	1.3953	0.997
24	1.3954	1.3861	1.3919	1.3916	1.3881	1.3922	0.998
25	1.3960	1.3869	1.3915	1.3918	1.3893	1.3927	0.998
26	1.3947	1.3856	1.3913	1.3922	1.3887	1.3927	0.998
27	1.3965	1.3873	1.3912	1.3921	1.3889	1.3935	0.998
28	1.3963	1.3863	1.3919	1.3914	1.3901	1.3939	0.998
29	1.3966	1.3857	1.3923	1.3912	1.3897	1.3929	0.997
30	1.3956	1.3870	1.3910	1.3925	1.3884	1.3939	0.998
31	1.3968	1.3868	1.3916	1.3915	1.3897	1.3933	0.998
32	1.3961	1.3872	1.3913	1.3919	1.3896	1.3941	0.998
33	1.3995	1.3863	1.3932	1.3918	1.3896	1.3939	0.995
34	1.3970	1.3869	1.3918	1.3933	1.3889	1.3969	0.996
35	1.3970	1.3859	1.3922	1.3912	1.3902	1.3927	0.997
36	1.3954	1.3874	1.3911	1.3924	1.3888	1.3943	0.998
37	1.4007	1.3856	1.3933	1.3916	1.3903	1.3964	0.993
38	1.3992	1.3876	1.3915	1.3934	1.3883	1.3979	0.994

Trp (6-membered ring)

molecule	R1	R2	R3	R4	R5	R6	HOMA
1	1.3967	1.3886	1.3906	1.3932	1.3878	1.3969	0.996
2	1.3997	1.3852	1.3932	1.3908	1.3915	1.3940	0.995
3	1.3982	1.3846	1.3941	1.3904	1.3919	1.3928	0.996
4	1.3973	1.3862	1.3918	1.3916	1.3899	1.3944	0.997
5	1.3971	1.3871	1.3915	1.3920	1.3889	1.3946	0.997
6	1.3953	1.3883	1.3909	1.3931	1.3886	1.3949	0.998
7	1.3979	1.3855	1.3933	1.3908	1.3914	1.3923	0.996
8	1.3971	1.3875	1.3913	1.3922	1.3892	1.3955	0.997
9	1.3982	1.3864	1.3921	1.3915	1.3903	1.3943	0.996
10	1.3979	1.3852	1.3933	1.3907	1.3913	1.3931	0.996
11	1.3965	1.3860	1.3923	1.3912	1.3902	1.3932	0.997
12	1.3960	1.3873	1.3911	1.3924	1.3889	1.3937	0.998
13	1.3959	1.3873	1.3912	1.3920	1.3890	1.3932	0.998
14	1.3971	1.3865	1.3922	1.3915	1.3904	1.3935	0.997
15	1.3960	1.3863	1.3918	1.3914	1.3901	1.3932	0.998
16	1.3964	1.3876	1.3914	1.3923	1.3894	1.3943	0.998
17	1.3953	1.3875	1.3908	1.3926	1.3884	1.3942	0.998
18	1.3969	1.3856	1.3924	1.3910	1.3903	1.3926	0.997
19	1.3959	1.3883	1.3908	1.3932	1.3882	1.3951	0.998
20	1.3979	1.3854	1.3932	1.3909	1.3912	1.3931	0.996
21	1.3962	1.3870	1.3914	1.3922	1.3889	1.3937	0.998
22	1.3963	1.3882	1.3909	1.3927	1.3889	1.3948	0.998
23	1.3966	1.3876	1.3911	1.3924	1.3887	1.3953	0.997
24	1.3954	1.3861	1.3919	1.3916	1.3881	1.3922	0.998
25	1.3960	1.3869	1.3915	1.3918	1.3893	1.3927	0.998
26	1.3947	1.3856	1.3913	1.3922	1.3887	1.3927	0.998
27	1.3965	1.3873	1.3912	1.3921	1.3889	1.3935	0.998
28	1.3963	1.3863	1.3919	1.3914	1.3901	1.3939	0.998
29	1.3966	1.3857	1.3923	1.3912	1.3897	1.3929	0.997
30	1.3956	1.3870	1.3910	1.3925	1.3884	1.3939	0.998
31	1.3968	1.3868	1.3916	1.3915	1.3897	1.3933	0.998
32	1.3961	1.3872	1.3913	1.3919	1.3896	1.3941	0.998
33	1.3995	1.3863	1.3932	1.3918	1.3896	1.3939	0.995
34	1.3970	1.3869	1.3918	1.3933	1.3889	1.3969	0.996
35	1.3970	1.3859	1.3922	1.3912	1.3902	1.3927	0.997
36	1.3954	1.3874	1.3911	1.3924	1.3888	1.3943	0.998
37	1.4007	1.3856	1.3933	1.3916	1.3903	1.3964	0.993

Trp (5-membered)

molecule	R1	R2	R3	R4	R5	HOMA
1	1.3694	1.4383	1.4186	1.3750	1.3777	0.539
2	1.3686	1.4389	1.4163	1.3768	1.3799	0.525
3	1.3703	1.4421	1.4171	1.3770	1.3784	0.507
4	1.3683	1.4390	1.4179	1.3752	1.3789	0.526
5	1.3672	1.4388	1.4169	1.3761	1.3810	0.514
6	1.3689	1.4390	1.4170	1.3757	1.3794	0.529
7	1.3684	1.4395	1.4174	1.3756	1.3788	0.523
8	1.3683	1.4384	1.4179	1.3750	1.3798	0.528
9	1.3686	1.4401	1.4171	1.3761	1.3795	0.515
10	1.3687	1.4395	1.4178	1.3758	1.3792	0.521
11	1.3681	1.4386	1.4164	1.3765	1.3805	0.523
12	1.3669	1.4377	1.4167	1.3763	1.3802	0.527
13	1.3674	1.4390	1.4171	1.3757	1.3795	0.522
14	1.3669	1.4385	1.4172	1.3762	1.3810	0.515
15	1.3686	1.4389	1.4175	1.3753	1.3792	0.528
16	1.3654	1.4372	1.4170	1.3762	1.3802	0.523
17	1.3674	1.4389	1.4171	1.3759	1.3813	0.514
18	1.3683	1.4401	1.4176	1.3757	1.3797	0.513
19	1.3679	1.4388	1.4164	1.3766	1.3806	0.520
20	1.3677	1.4390	1.4168	1.3758	1.3795	0.523
21	1.3669	1.4379	1.4164	1.3762	1.3799	0.528
22	1.3672	1.4376	1.4168	1.3762	1.3800	0.529
23	1.3681	1.4400	1.4177	1.3753	1.3801	0.512
24	1.3671	1.4377	1.4181	1.3755	1.3783	0.532
25	1.3680	1.4383	1.4172	1.3751	1.3788	0.534
26	1.3687	1.4388	1.4176	1.3753	1.3792	0.529
27	1.3678	1.4385	1.4165	1.3748	1.3796	0.533
28	1.3711	1.4411	1.4157	1.3774	1.3776	0.525
29	1.3667	1.4377	1.4176	1.3759	1.3796	0.525
30	1.3688	1.4387	1.4174	1.3745	1.3803	0.529
31	1.3671	1.4376	1.4165	1.3760	1.3795	0.533
32	1.3685	1.4384	1.4164	1.3750	1.3807	0.532
33	1.3651	1.4380	1.4173	1.3758	1.3803	0.515
34	1.3733	1.4433	1.4166	1.3765	1.3764	0.517
35	1.3727	1.4404	1.4161	1.3766	1.3774	0.539
36	1.3690	1.4398	1.4173	1.3759	1.3789	0.522
37	1.3684	1.4385	1.4171	1.3753	1.3786	0.535

His						
**molecule**	**R1**	**R2**	**R3**	**R4**	**R5**	**HOMA**
1	1.3677	1.3764	1.3590	1.3119	1.3833	0.732
2	1.3672	1.3794	1.3543	1.3123	1.3795	0.734
3	1.3688	1.3776	1.3586	1.3113	1.3826	0.745
4	1.3668	1.3790	1.3541	1.3131	1.3802	0.729
5	1.3718	1.3751	1.3117	1.3609	1.3788	0.788
6	1.3690	1.3773	1.3600	1.3101	1.3814	0.747
7	1.3711	1.3746	1.3113	1.3613	1.3790	0.780
8	1.3705	1.3752	1.3105	1.3625	1.3792	0.769
9	1.3699	1.3776	1.3612	1.3094	1.3801	0.757
10	1.3708	1.3751	1.3114	1.3614	1.3775	0.779
11	1.3706	1.3759	1.3095	1.3633	1.3809	0.764
12	1.3688	1.3771	1.3603	1.3103	1.3822	0.743
13	1.3698	1.3782	1.3613	1.3094	1.3796	0.756
14	1.3689	1.3775	1.3617	1.3094	1.3796	0.747
15	1.3680	1.3783	1.3609	1.3103	1.3804	0.735
16	1.3671	1.3777	1.3611	1.3099	1.3784	0.729
17	1.3722	1.3785	1.3596	1.3095	1.3794	0.783
18	1.3681	1.3757	1.3613	1.3109	1.3806	0.741
19	1.3706	1.3773	1.3605	1.3105	1.3800	0.767
20	1.3711	1.3747	1.3110	1.3617	1.3796	0.778
21	1.3676	1.3793	1.3542	1.3127	1.3808	0.737
22	1.3706	1.3750	1.3119	1.3607	1.3783	0.777
23	1.3695	1.3774	1.3593	1.3109	1.3818	0.753
24	1.3692	1.3775	1.3614	1.3097	1.3795	0.751
25	1.3692	1.3775	1.3614	1.3097	1.3795	0.751
26	1.3689	1.3775	1.3614	1.3093	1.3793	0.747
27	1.3696	1.3771	1.3612	1.3095	1.3791	0.757
28	1.3696	1.3769	1.3603	1.3108	1.3818	0.754
29	1.3706	1.3761	1.3086	1.3648	1.3811	0.759
30	1.3695	1.3770	1.3601	1.3097	1.3806	0.755
31	1.3693	1.3782	1.3611	1.3097	1.3796	0.751
32	1.3693	1.3759	1.3090	1.3642	1.3803	0.749
33	1.3694	1.3772	1.3608	1.3096	1.3793	0.755
34	1.3681	1.3768	1.3617	1.3103	1.3806	0.737
35	1.3701	1.3752	1.3097	1.3630	1.3803	0.762
36	1.3706	1.3788	1.3602	1.3093	1.3791	0.765
37	1.3722	1.3777	1.3075	1.3647	1.3797	0.775
38	1.3706	1.3774	1.3604	1.3103	1.3799	0.768
39	1.3707	1.3749	1.3100	1.3628	1.3787	0.773
40	1.3705	1.3760	1.3088	1.3647	1.3811	0.760
41	1.3700	1.3766	1.3087	1.3649	1.3818	0.751
42	1.3690	1.3772	1.3611	1.3095	1.3791	0.750
43	1.3699	1.3771	1.3612	1.3094	1.3791	0.761
44	1.3693	1.3754	1.3088	1.3642	1.3809	0.748

45	1.3704	1.3779	1.3603	1.3094	1.3802	0.763
46	1.3671	1.3781	1.3607	1.3103	1.3793	0.726
47	1.3681	1.3756	1.3087	1.3651	1.3809	0.732
48	1.3704	1.3787	1.3602	1.3092	1.3797	0.762
49	1.3718	1.3788	1.3595	1.3095	1.3790	0.779
50	1.3690	1.3771	1.3608	1.3099	1.3790	0.752
51	1.3742	1.3748	1.3114	1.3605	1.3777	0.814
52	1.3710	1.3749	1.3115	1.3613	1.3768	0.783
53	1.3718	1.3772	1.3078	1.3648	1.3791	0.773
54	1.3709	1.3747	1.3097	1.3635	1.3802	0.770
55	1.3682	1.3756	1.3087	1.3650	1.3798	0.737
56	1.3705	1.3751	1.3090	1.3650	1.3819	0.759
57	1.3709	1.3771	1.3079	1.3648	1.3796	0.763
58	1.3695	1.3752	1.3087	1.3642	1.3806	0.752
59	1.3664	1.3779	1.3604	1.3106	1.3795	0.718
60	1.3689	1.3760	1.3597	1.3103	1.3802	0.752
61	1.3699	1.3762	1.3090	1.3643	1.3827	0.750
62	1.3696	1.3777	1.3593	1.3104	1.3810	0.755
63	1.3708	1.3779	1.3073	1.3652	1.3810	0.757
64	1.3708	1.3762	1.3088	1.3640	1.3838	0.757
65	1.3697	1.3776	1.3071	1.3649	1.3822	0.742

Table S25. The HOMA index of some model rings calculated at the B3LYP/D3/aug-cc-pVTZ level.

molecule	R1	R2	R3	R4	R5	R6	HOMA
4Me-1H-imidazole	1.3604	1.3794	1.3692	1.3794	1.3101		0.749
5Me-1H-imidazole	1.3078	1.3772	1.3691	1.3799	1.3662		0.740
3-Me-indole(6)	1.4172	1.3933	1.3851	1.4059	1.3839	1.4003	0.954
3-Me-indole(5)	1.4172	1.3748	1.3829	1.3662	1.4374		0.517
p-cresol	1.3973	1.3865	1.3921	1.3902	1.3917	1.3923	0.997
Toluene	1.3956	1.3903	1.3904	1.3904	1.3903	1.3956	0.998
pyrrole	1.3737	1.4217	1.3737	1.3715	1.3715		0.738

Table S26. The population averaged $HOMA_{pav}$ index for: Phe, Tyr, Trp and His conformers and the HOMA index of some model rings calculated at the B3LYP/D3/aug-cc-pVTZ level.

molecule	$HOMA_{pav}$
Trp (6)	0.951
Phe	0.996
Trp (5)	0.531
His	0.734
Tyr	0.996
3-Me-indole (6)	0.954
benzene	1.000
toluene	0.998
p-cresol	0.997
pyrrole	0.738
3-Me-indole (5)	0.517
5Me-1H-imidazole	0.739
4Me-1H-imidazole	0.749
